# Inhibition of non-homologous end joining of gamma ray-induced DNA double-strand breaks by cAMP signaling in lung cancer cells

**DOI:** 10.1038/s41598-020-71522-9

**Published:** 2020-09-02

**Authors:** Sung-Eun Noh, Yong-Sung Juhnn

**Affiliations:** Department of Biochemistry and Molecular Biology, Department of Biomedical Sciences and Cancer Research Institute, Seoul National University College of Medicine, 103 Daehak-ro, Jongno-gu, Seoul, 03080 Korea

**Keywords:** Biochemistry, Cancer, Molecular biology, Medical research, Molecular medicine, Oncology

## Abstract

DNA double-strand breaks (DSB) are formed by various exogenous and endogenous factors and are repaired by homologous recombination and non-homologous end joining (NHEJ). DNA-dependent protein kinase (DNA-PK) is the principal enzyme for NHEJ. We explored the role and the underlying mechanism of cAMP signaling in the NHEJ repair of DSBs resulted from gamma ray irradiation to non-small cell lung cancer (NSLC) cells. Activated cAMP signaling by expression of an activated stimulatory GTP-binding protein or by pretreatment with isoproterenol and prostaglandin E2, delayed the repair of DSBs resulted from gamma ray irradiation, and the delaying effects depended on protein kinase A (PKA). Activated cAMP signaling suppressed XRCC4 and DNA ligase IV recruitment into DSB foci, and reduced phosphorylation at T2609 in DNA-PK catalytic subunit (DNA-PKcs) with a concomitant increase in phosphorylation at S2056 in PKA-dependent ways following gamma ray irradiation. cAMP signaling decreased phosphorylation of T2609 by protein phosphatase 2A-dependent inhibition of ATM. We conclude that cAMP signaling delays the repair of gamma ray-induced DNA DSBs in NSLC cells by inhibiting NHEJ via PKA-dependent pathways, and that cAMP signaling differentially modulates DNA-PKcs phosphorylation at S2056 and T2609, which might contribute to the inhibition of NHEJ in NSLC cells.

## Introduction

DNA double-strand breaks (DSBs) are localized chromosomal injuries bearing breaks at two DNA strands. They can be produced by various exogenous factors such as chemical mutagens, anticancer chemotherapeutic drugs and high-energy radiation, and by endogenous processes including DNA replication and recombination of V(D)J immunoglobulin genes^1–3^. DSBs are harmful lesions that, when they are not repaired or not properly repaired, can result in chromosomal mutations, apoptosis, aging, and diseases including neurodegeneration and cancer^4–6^.

Homologous recombination (HR) and non-homologous end-joining (NHEJ) are the major mechanisms for DSB repair in vertebrates. HR requires a homologous DNA molecule as a template to recover any lost sequence information at the break sites and is therefore free of errors. HR, thus, preferentially repairs DNA in G2 and late S phase of the cell cycle. NHEJ restores DNA integrity by linking the two broken ends without using extensive sequence homology, and is activated during the whole cell cycle^7^. NHEJ repair often results in small deletions and insertions at the repaired site. More than two NHEJ subtypes operate in many cells including classical NHEJ and alternative NHEJ. Classical NHEJ can be broken down into several stages, such as synapse, end-processing, ligation and complex dissociation, and it requires numerous enzymes, namely, DNA-dependent protein kinase (DNA-PK), nucleases, DNA polymerases, and ligases. DNA-PK is a nuclear serine/threonine kinase, and exists as complex of a catalytic subunit (DNA-PKcs) and a regulatory heterodimeric Ku complex (Ku70/Ku80)^1^. DNA-PK is one of the core molecules essential to NHEJ, and known to phosphorylate many proteins, including DNA-PK itself. DNA-PK is also suggested to have other functions other than DNA repair such as transcription and mitosis^8^.

Cyclic AMP (3′,5′-cyclic adenosine monophosphate, cAMP) acts as a second messenger molecule, produced by adenylyl cyclases, and hydrolyzed by cyclic nucleotide phosphodiesterases. Adenylyl cyclases are activated by stimulatory GTP-binding proteins, which turn into active conformation by seven transmembrane receptors bound by numerous signaling molecules including glucagon and epinephrine. The elevated cellular cAMP levels can activate effector molecules, including protein kinase A (PKA, cAMP-dependent protein kinase), cyclic nucleotide-gated ion channels and exchange protein directly activated by cAMP (Epac). A variety of physiological responses are regulated by these effector molecules, including energy metabolism, expression of genes, cellular proliferation and death^9^. Hence, various alterations in the activity of cAMP signaling was reported in many diseases, including cancer, and therefore cAMP signaling might be a potential target to develop novel treatments for many diseases^10,11^.

cAMP signaling was reported to affect some DNA damage repair mechanisms, including base excision repair (BER)^12,13^ and nucleotide excision repair (NER)^14^. Furthermore, cAMP signaling was reported to reduce DSB formation by reactive oxygen species (ROS) in melanoma cells. Nevertheless, it is not clearly known how cAMP signaling affects DNA DSB repair. Hence, we made a hypothesis that the cAMP signaling might affect DSB repair by regulating key DSB repair mechanisms, including the NHEJ. The present study investigated how cAMP signaling affects the repair of DSBs induced by gamma ray irradiation and its underlying mechanisms in non-small cell lung cancer (NSLC) cells. We found that cAMP signaling delays the repair of DNA DSBs induced by gamma ray through inhibiting NHEJ in PKA-dependent pathways in NSLC cells, which is suggested as a consequence of the differential modulation of DNA-PKcs phosphorylation at T2609 and S2056.

## Results

### cAMP signaling delays repair of DNA DSBs resulted from gamma ray irradiation in NSLC cells

**Figure 1:**
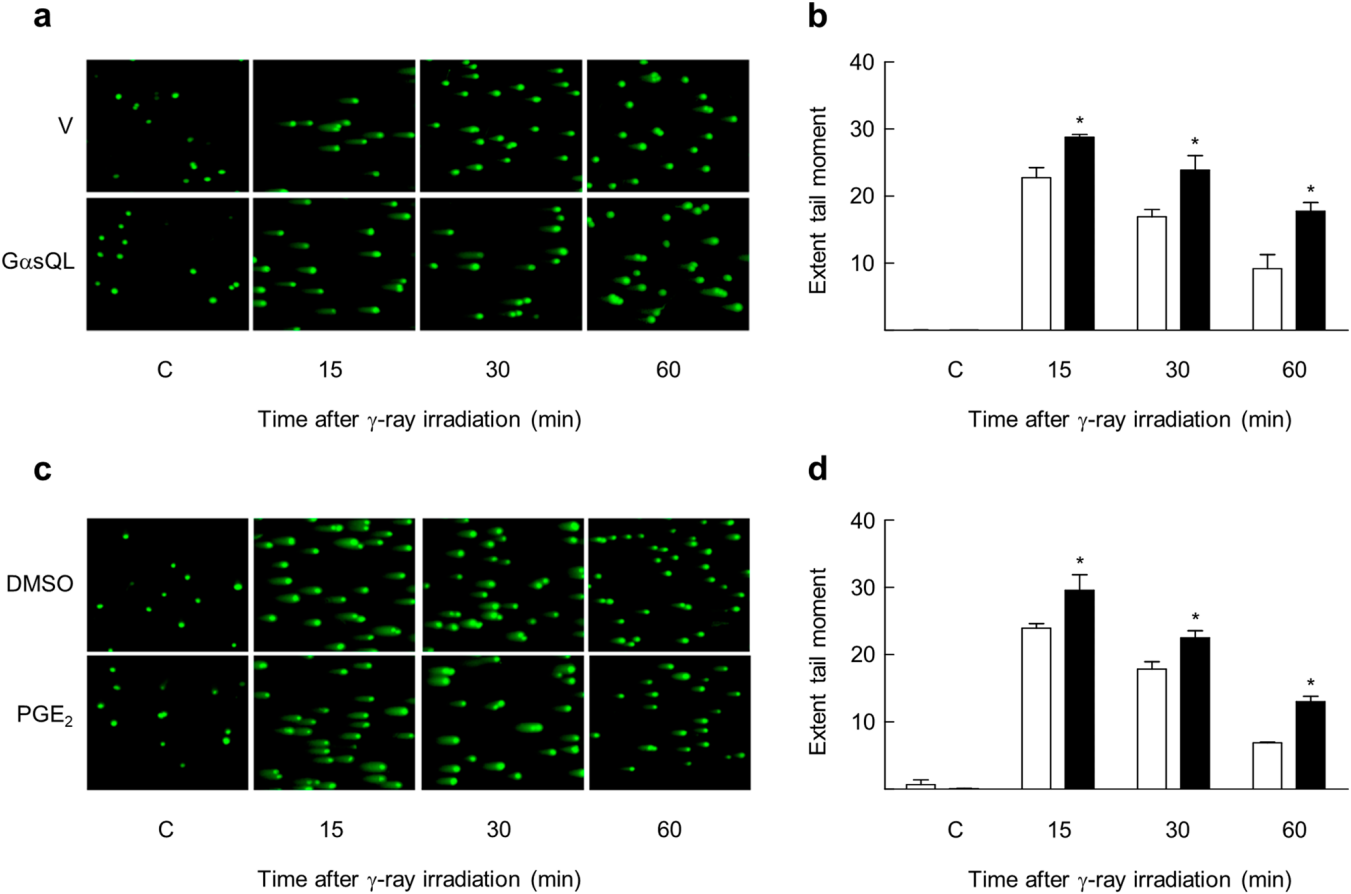
cAMP signaling delayed the repair of DNA double-strand breaks (DSBs) resulted from gamma ray irradiation in H1299 NSLC cells. (**a**) Effects of GαsQL on DNA DSBs following γ-ray irradiation. (**b**) A bar graph of extent tail moments as determined by analyzing the images shown in (**a**). The empty bar indicates the vector-transfected cells, and the filled bar represents the GαsQL-transfected cells. (**c**) Effect of PGE2 on DNA DSBs following γ-ray irradiation. (**d**) A bar graph of extent tail moments as determined by analyzing the images of (**c**). The empty bar represents the DMSO-treated control cells, and the filled bar represents the PGE2-treated cells. H1299 NSLC cells were transiently transfected with the GαsQL or pcDNA3.1 vector using Lipofectamine 3000 and maintained for 24 h. The transfected cells and the cells treated with 20 μM PGE2 for 30 min before irradiation with gamma rays (5 Gy), and the resulting DNA damage was assessed by neutral comet assay at the indicated time points. DNA was stained, and the stained DNA image was recorded using a confocal microscope. The extent tail moment was defined as Tail DNA% × Length of Tail and calculated using the OpenComet program. Each column represents the mean ± S.E. of three independent experiments. An asterisk (*) represents differences showing statistical significance from the respective control (*P* ≤ 0.05, Mann–Whitney U test). Full blots are shown in Supplementary Fig. S26.

**Figure 2:**
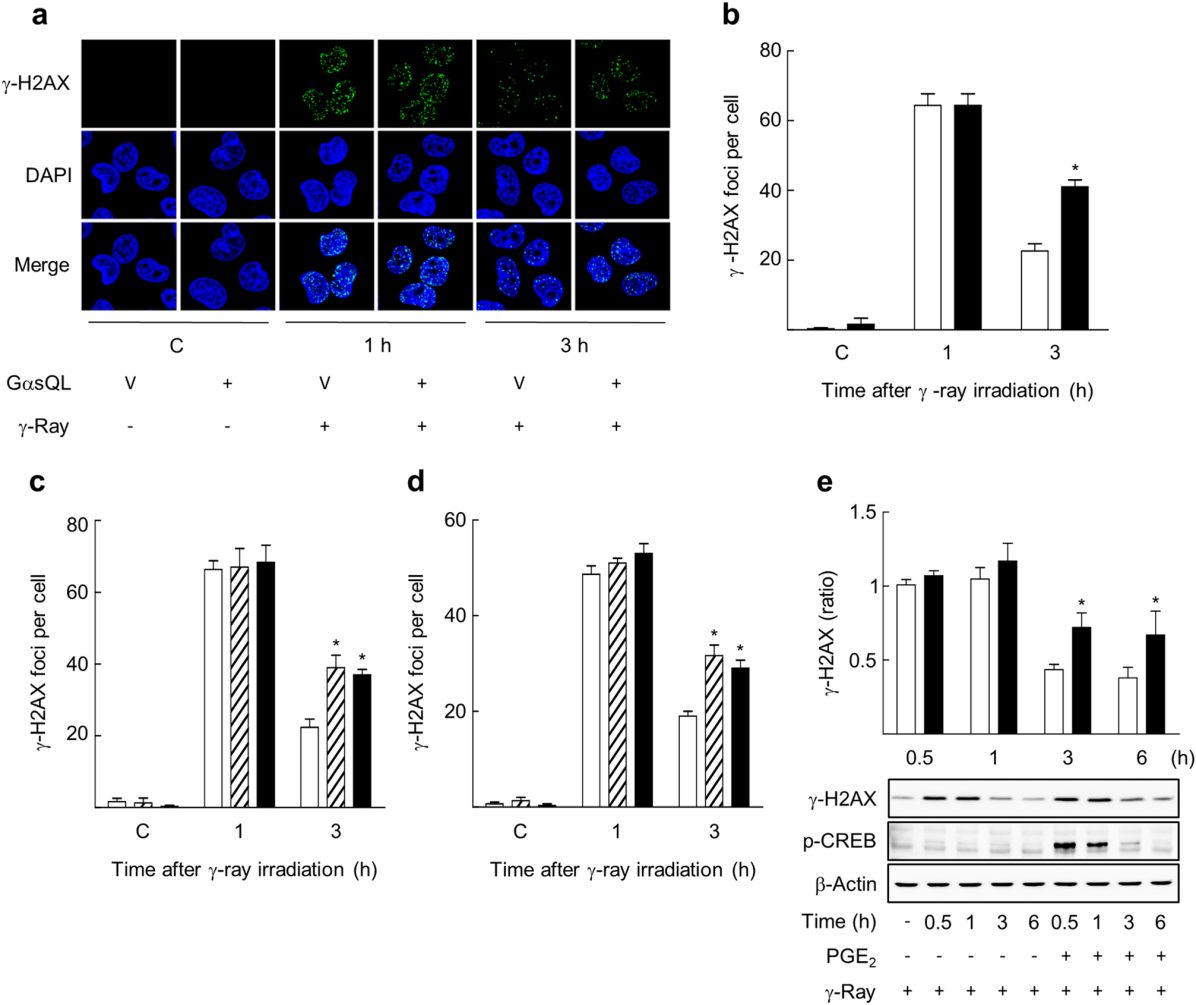
cAMP signaling delayed the DNA DSB repair following gamma ray irradiation in H1299 and A549 NSLC cells. (**a**) Effects of GαsQL on DNA DSBs resulting from gamma ray irradiation of H1299 cells. (**b**) A bar graph of γ-H2AX foci per cell, as obtained by analyzing the images shown in (**a**). Empty bars represent the controls, and filled bars represent the GαsQL-transfected cells. (**c**) Effects of isoproterenol and PGE2 on DNA DSBs resulting from gamma ray irradiation in H1299 cells. (**d**) Effects of isoproterenol and PGE2 on DNA DSBs resulting from gamma ray irradiation in A549 cells. GαsQL or pcDNA3.1 vector plasmid were transfected to NSLC cells (H1299 or A549), and the transfected cells were maintained for 24 h. The transfected cells and the cells pretreated with 20 μM PGE2 or 1 μM isoproterenol (ISO) for 30 min were exposed to gamma rays (5 Gy). The irradiated cells were fixed, permeabilized, and stained with an anti-γ-H2AX antibody and DAPI. γ-H2AX was visualized as green, and DAPI was visualized as blue. The images of stained cells were acquired and analyzed. (**e**) Effects of PGE2 treatment after irradiation on DNA DSBs resulting from gamma ray irradiation in H1299 cells. The cells were treated with 20 μM PGE2 at 10 min after irradiation (5 Gy), and harvested at the indicated times for western blot analysis. Empty bars represent the control, filled bars represent the PGE2-treated cells, and slant bars represent the isoproterenol-treated cells. Each column represents the mean ± S.E. of three independent experiments. An asterisk (*) represents differences showing statistical significance from the respective control (*P* ≤ 0.05, Mann–Whitney U test). Full blots are shown in Supplementary Fig. S26.

To investigate the effect of cAMP signaling on the repair of DNA DSBs resulted from gamma ray irradiation, we activated cAMP signaling by expression of a constitutively active Gαs (GαsQL) that stimulates adenylate cyclase constitutively or by treatment with isoproterenol that activates β-adrenergic receptors and PGE2 that activates EP receptors. The DSBs were analyzed by neutral comet assay following gamma ray irradiation of human NSLC cells (H1299 and A549 cells). DSBs caused by gamma ray irradiation increased in proportion to the radiation dose (2 ~ 20 Gy), and were repaired within 180 min after irradiation in H1299 cells (Supplementary Fig. S1). After irradiation of gamma ray (5 Gy) to vector-transfected or DMSO-pretreated H1299 cells, the extent tail moments of damaged DNA was reduced by more than half at 60 min after irradiation, but considerably slower reduction in damaged DNA tails was observed in cells expressing GαsQL (Fig. 1a,b) and in cells pre-treated with PGE2 (Fig. 1c,d), which showed more tails until 60 min after irradiation. Activation of cAMP signaling also enhanced radiation-induced γ-H2AX foci, a biomarker of DSB, at 3 h after irradiation in H1299 cells transfected with GαsQL (Fig. 2a,b), or pretreated with isoproterenol (Fig. 2c and Supplementary Fig. S2) or PGE2 (Fig. 2c and Supplementary Fig. S3). The delays in disappearance of gamma ray-induced γ-H2AX foci following treatment with isoproterenol or PGE2 were also observed in A549 lung cancer cells (Fig. 2d and Supplementary Fig. S4, S5). Moreover, activation of cAMP signaling by PGE2 treatment after irradiation also delayed the disappearance of gamma ray-induced γ-H2AX with substantial decrease in the slope of disappearance (Fig. 2e). In addition, activation of cAMP signaling by PGE2 pretreatment also delays the disappearance of γ-H2AX induced by treatment with neocarzinostatin, a radiomimetic, in H1299 and A549 NSLC cells (Supplementary Fig. S6, S7). The results shows that cAMP signaling delays the repair of DSBs resulted from gamma ray irradiation in NSLC cells.

### cAMP signaling delays NHEJ DNA repair by a PKA-dependent manner

**Figure 3:**
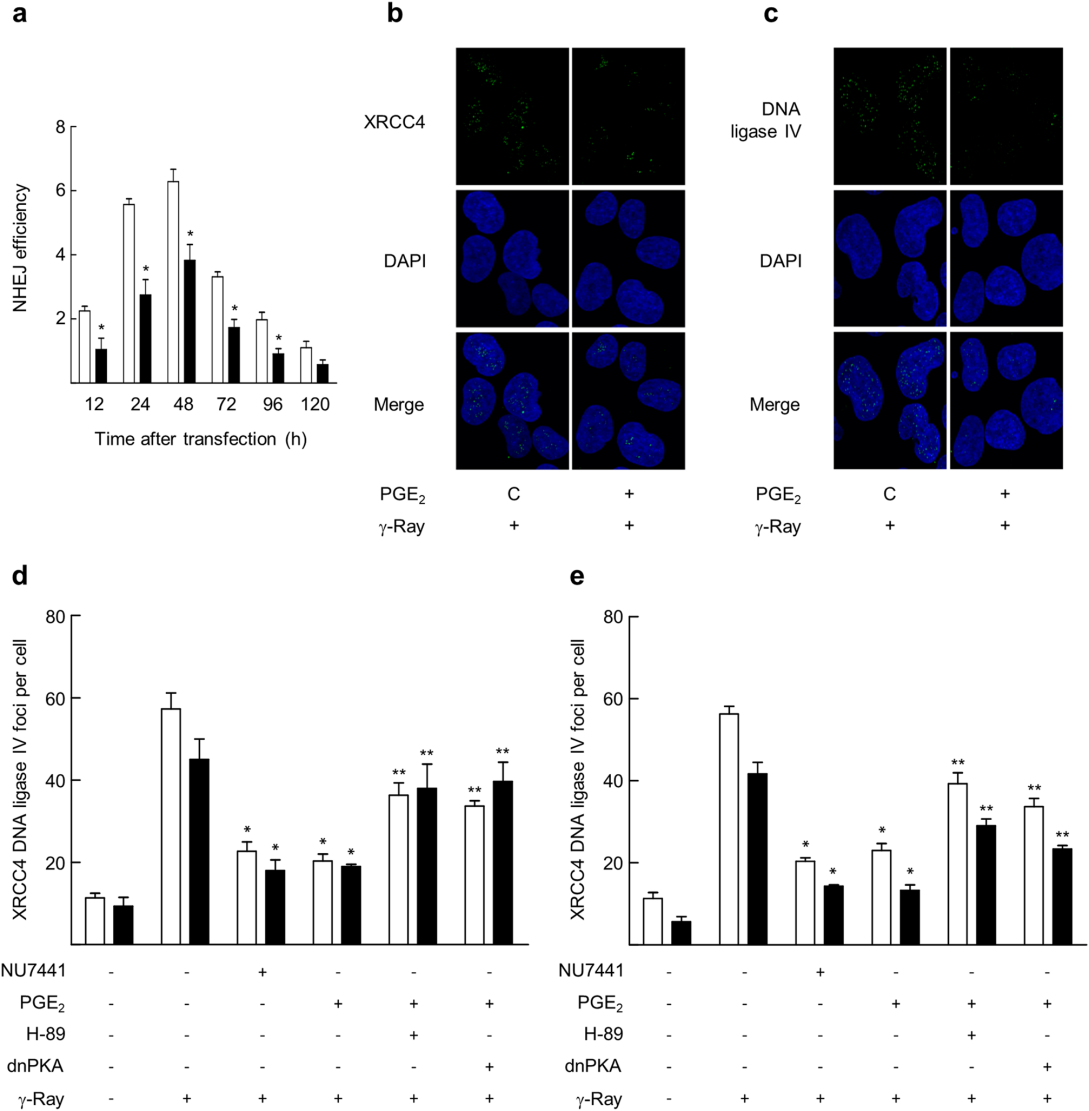
cAMP signaling delayed the repair of DNA DSBs by NHEJ. (**a**) Effects of GαsQL on NHEJ repair of the DSBs following I-SceI endonuclease digestion. H1299 cells were transfected with the linearized NHEJ reporter, pDsRed-N2, GαsQL, and control vectors using Lipofectamine 3000. The transfected cells were harvested after the times indicated, and the GFP fluorescence was measured using a flow cytometer. The repair efficiencies of NHEJ was defined as the ratio of green fluorescence from repaired reporter GFP to the red fluorescence from DsRed control. The empty bar shows vector-transfected cells, and the filled bar shows GαsQL-transfected cells. (**b**) Effects of PGE2 on the recruitment of XRCC4 following γ-ray irradiation. (**c**) Effects of PGE2 on the recruitment of DNA-ligase IV following γ-ray irradiation. (**d**, **e**) Effects of NU7441, H-89, and dominant negative PKA on the recruitments of XRCC4 and DNA-ligase IV following irradiation with gamma ray in H1299 cells (**d**) and in A549 cells (**e**). Empty bars represent XRCC4, filled bars represent DNA ligase IV. NSLC cells (H1299 and A549 cells) transfected with dominant negative PKA (dnPKA) and the cells pretreated with 10 μM H-89 for 30 min were incubated with 20 μM PGE2 or 5 μM NU7441 for 30 min before gamma ray irradiation (5 Gy). The irradiated cells were harvested after 1 h and were reacted with specific antibodies and DAPI. The images of the stained cells were acquired by confocal microscopy and analyzed. Each column represents the mean ± S.E. of three independent experiments. An asterisk (*) represents differences showing statistical significance from the respective control (*P* ≤ 0.05, Mann–Whitney U test), and double asterisks (**) represents differences showing statistical significance from the PGE2-treated cells (*P* ≤ 0.05, Mann–Whitney U test). Full blots are shown in Supplementary Fig. S26.

Next, to study the mechanism of cAMP signaling to delay DSB repair, the effect of cAMP signaling on non-homologous end joining (NHEJ) was examined. The activity of NHEJ was assessed by measuring fluorescence resulting from the repaired reporter plasmid that had been linearized with I-SceI endonuclease before transient transfection. cAMP signaling activated by GαsQL expression decreased for 120 h the NHEJ efficiency (Fig. 3a and Supplementary Fig. S8) compared with the control efficiency in H1299 cells. Expression of GαsQL in A549 cells also reduced NHEJ efficiency (Supplementary Fig. S9). This results indicate that cAMP signaling delays the repair of I-SceI-induced DSBs in the reporter plasmid by inhibiting NHEJ.

Then, to examine how cAMP signaling inhibits NHEJ, we evaluated the radiation-induced recruitment of key components of the NHEJ after activation of cAMP signaling with PGE2. Pretreatment with PGE2 did not significantly change the recruitment of DNA-PKcs, Ku70 or Ku80 (Supplementary Fig. S10–S12). However, pretreatment with PGE2 significantly reduced the recruitments of XRCC4 and DNA ligase IV to DSB foci without increasing protein levels of XRCC4 and DNA ligase IV (Fig. 3b,c and Supplementary Fig. S13, S14). The recruitments of XRCC4 and DNA ligase IV was prevented by pretreatment with NU7441, a DNA-PK inhibitor (Fig. 3d and Supplementary Fig. S15, S16). The inhibitory effect of PGE2 on the recruitments of XRCC4 and DNA ligase IV was decreased by pretreatment with H-89 (a PKA inhibitor) or by expression of dominant negative PKA (Fig. 3d). Similar effects of cAMP on XRCC4 and DNA ligase IV recruitment were observed with A549 cells (Fig. 3e and Supplementary Fig. S17, S18). The result suggests that activation of cAMP signaling delays NHEJ repair of DSBs by decreasing the recruitment of DNA ligase IV and XRCC4, the major components of NHEJ, to DSB foci in PKA-dependent pathways.

### cAMP signaling modulated phosphorylation at S2056 and T2609 of DNA-PKcs induced by gamma ray irradiation

**Figure 4:**
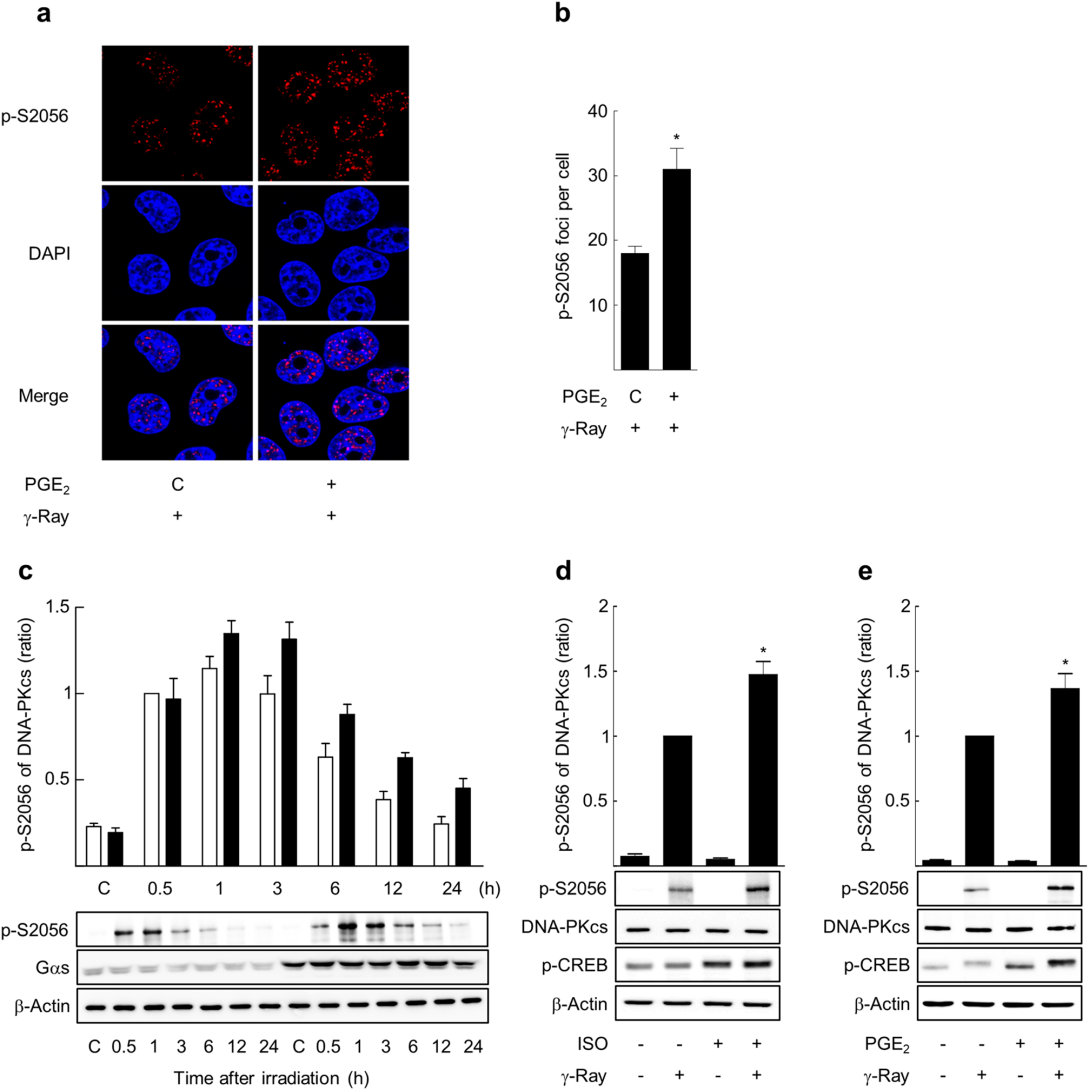
cAMP signaling increased phosphorylation of DNA-PKcs at S2056 resulting from gamma ray irradiation in H1299 cells. (**a**) Effects of PGE2 upon S2056 phosphorylation following γ-ray irradiation, as determined by confocal microscopy. H1299 cells on microscope cover glass were treated with 20 μM PGE2 (30 min) before gamma ray irradiation (5 Gy), harvested 1 h after irradiation, and stained with an antibody against p-S2056 and with DAPI. The stained cells were analyzed by confocal microscopy (n = 3). (**b**) A bar graph of S2056 phosphorylation obtained by analyzing the images shown in Fig. 4a. (**c**) Effect of GαsQL expression on S2056 phosphorylation following γ-ray irradiation (n = 4). Empty bars represent vector-transfected cells, and filled bars represent GαsQL-transfected cells. (**d**) Effects of isoproterenol (ISO) upon S2056 phosphorylation following γ-ray irradiation (n = 5). (**e**) Effect of PGE2 on S2056 phosphorylation following gamma ray irradiation (n = 5). GαsQL and pcDNA3.1 vector control were transfected into H1299 cells and maintained for 24 h. The transfected cells and the cells pretreated with 20 μM PGE2 or 1 μM isoproterenol for 30 min were exposed to gamma rays (5 Gy) and harvested at 1 h or at the indicated times for western blot analysis. Each column represents the mean ± S.E. of 3–5 independent experiments. An asterisk (*) represents differences showing statistical significance from the respective control (*P* ≤ 0.05, Mann–Whitney U test). Full blots are shown in Supplementary Fig. S26.

Subsequently, to explore the mechanisms through which cAMP signaling modulate the recruitments of DNA ligase IV and XRCC4, we analyzed phosphorylation of DNA-PKcs, the main enzyme in the NHEJ, induced by gamma ray irradiation, because phosphorylation of DNA-PKcs is suggested to regulates its functions, including activation and accessibility of DNA for processing and ligation. The phosphorylation of DNA-PKcs at S2056 in PQR region and at T2609 in ABCDE region induced by gamma ray irradiation was analyzed after cAMP signaling was stimulated by expression of GαsQL or by pretreatment with isoproterenol or PGE2. The intensity of the fluorescence from phosphorylated S2056 was increased in proportion to the radiation dose from 2 to 10 Gy 1 h after irradiation (Supplementary Fig. S19), and the fluorescence from phosphorylated S2056 was weak at 30 min and became strong at 60 min after gamma ray irradiation (Supplementary Fig. S20). Pretreatment with PGE2 increased the fluorescence from phosphorylated S2056 at 60 min after irradiation with gamma ray (Fig. 4a,b). The effects of cAMP signaling on DNA-PKcs phosphorylation were attested by western blotting. The DNA-PKcs phosphorylation at S2056 was increased at 30 min, reaching an apex at 1 h, and returned to basal levels at 12 h after irradiation in H1299 NSLC cells. The exogenous expression of GαsQL increased phosphorylation of DNA-PKcs at S2056 compared with the control from 0.5 to 24 h after irradiation (Fig. 4c). Activation of cAMP signaling by pretreatment of H1299 cells with isoproterenol or PGE2 similarly increased the phosphorylation of S2056 at 1 h after gamma ray irradiation (Fig. 4d,e). Similarly, pretreatment of A549 cells with isoproterenol or PGE2 also increased the phosphorylation of S2056 1 h after gamma ray irradiation (Supplementary Fig. S21).

**Figure 5:**
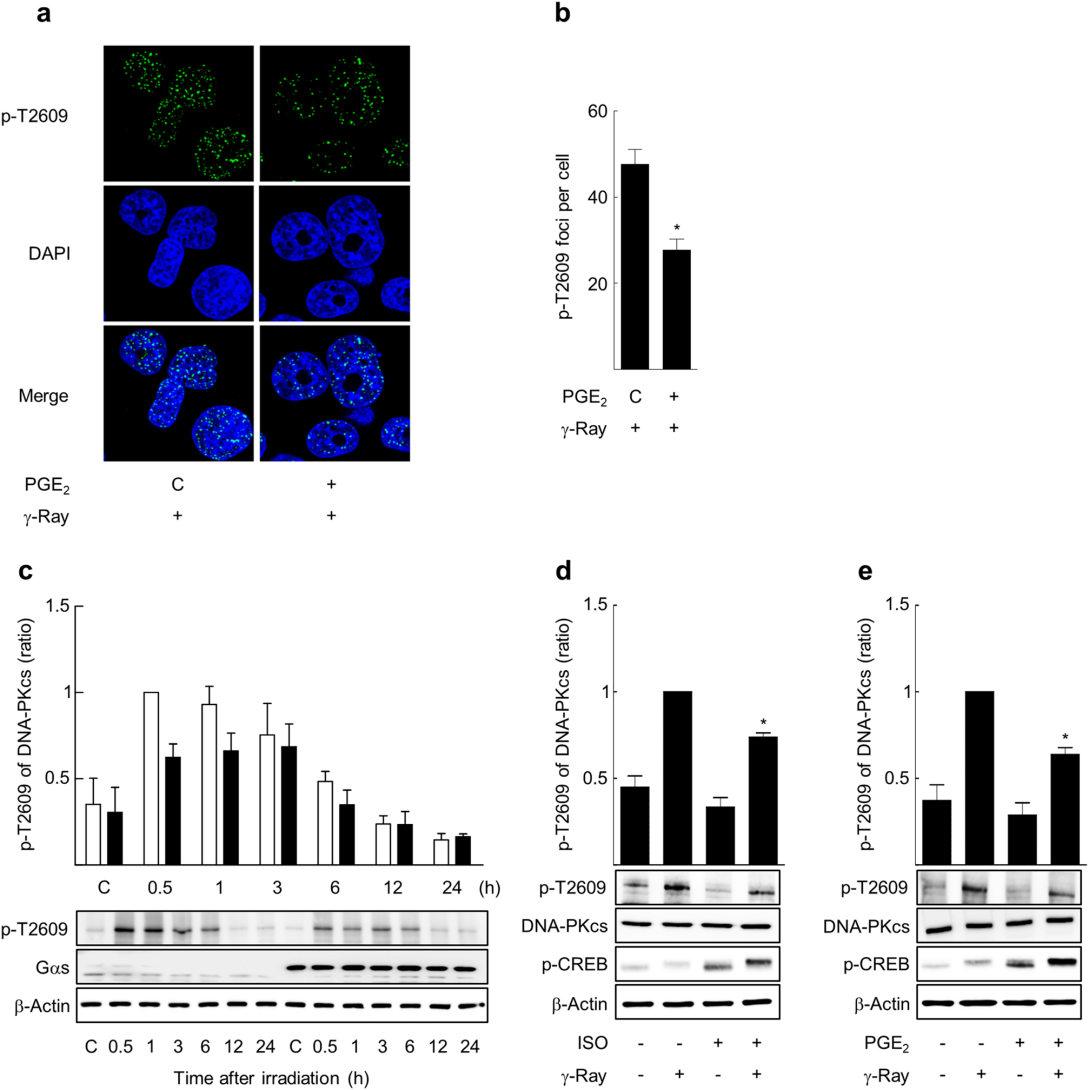
cAMP signaling decreased phosphorylation of radiation-induced DNA-PKcs at T2609 in H1299 NSLC cells. (**a**) Effects of PGE2 on T2609 phosphorylation following γ-ray irradiation, as determined by confocal microscopy. H1299 cells on microscope cover glass were treated with 20 μM PGE2 (30 min) before irradiation with gamma rays (5 Gy) and harvested after 30 min to stain with an antibody against p-T2609 and with DAPI. The stained cells were analyzed by confocal microscopy (n = 3). (**b**) A bar graph of T2609 phosphorylation obtained by analyzing the images shown in Fig. 5a. (**c**) Effect of GαsQL expression on T2609 phosphorylation following γ-ray irradiation (n = 4). Empty bars represent vector-transfected cells, and filled bars represents GαsQL-transfected cells. (**d**) Effects of PGE2 upon T2609 phosphorylation following gamma ray irradiation (n = 5). (**e**) Effects of isoproterenol on T2609 phosphorylation following γ-ray irradiation (n = 5). GαsQL plasmid and pcDNA3.1 vector control were transfected into H1299 cells and maintained for 24 h. The transfected cells and the cells pretreated with 20 μM PGE2 or 1 μM isoproterenol for 30 min were exposed to gamma rays (5 Gy) and harvested at 1 h or at the indicated times for western blot analysis. Each column represents the mean ± S.E. of 3–5 independent experiments. An asterisk (*) represents differences showing statistical significance from the respective control (*P* ≤ 0.05, Mann–Whitney U test). Full blots are shown in Supplementary Fig. S26.

The fluorescence was also increased from the phosphorylated T2609 in the ABCDE cluster in proportion to the radiation dose (Supplementary Fig. S22), and the fluorescence intensity at 30 min was comparable to that at 60 min after irradiation of H1299 cells, reaching a peak intensity earlier than does fluorescence from phosphorylated S2056 (Supplementary Fig. S23). Pretreatment with PGE2 decreased the fluorescence from phosphorylated T2609 at 30 min (Fig. 5a,b). Western blot analysis showed that the phosphorylation of DNA-PKcs at T2609 increased to reach a peak levels between 30 min and 1 h, and returned to ground state 12 h after gamma ray irradiation (Fig. 5c). The phosphorylation at T2609 was decreased by the activation of cAMP signaling through GαsQL expression compared to that of the vector-transfected control from 0.5 to 24 h after gamma ray irradiation (Fig. 5c). Activation of cAMP signaling by pretreatment with isoproterenol or PGE2 also decreased the phosphorylation of T2609 30 min after irradiation in H1299 cells (Fig. 5d,e) and A549 cells (Supplementary Fig. S24). These results indicate that cAMP signaling increases the DNA-PKcs phosphorylation at S2056 in PQR region and decreases the phosphorylation at T2609 in ABCDE region following gamma ray irradiation in H1299 cells and A549 cells.

### PKA mediated the cAMP effect on the DNA-PKcs phosphorylation at S2056 and T2609 induced by gamma ray irradiation

**Figure 6:**
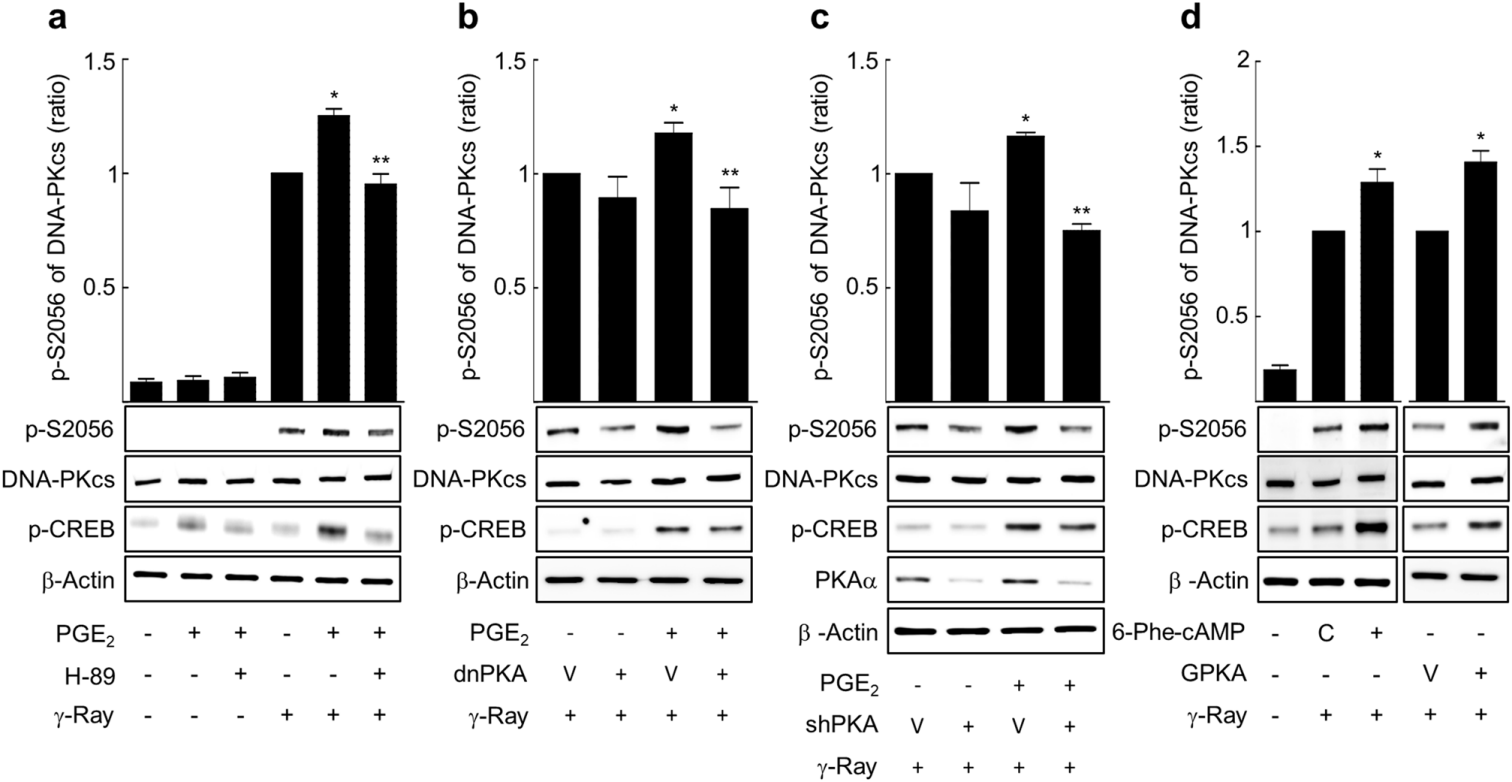
PKA mediated the cAMP effect on phosphorylation at S2056 of DNA-PKcs following gamma ray irradiation. (**a**) Effects of H-89 on phosphorylation at S2056 following gamma ray irradiation (n = 5). (**b**) Effects of dominant negative PKA (dnPKA) on phosphorylation at S2056 following gamma ray irradiation (n = 4). (**c**) Effects of PKA knockdown on phosphorylation at S2056 following gamma ray irradiation (n = 4). (**d**) Effect of 6-phe-cAMP and PKA catalytic subunit (GPKA) on phosphorylation at S2056 following gamma ray irradiation (n = 4). H1299 cells and H1299 cells treated with 10 μM H-89 (30 min) were reacted with 20 μM PGE2 or 50 μM 6-phe-cAMP (30 min), and then the cells were exposed to gamma irradiation (5 Gy). H1299 NSLC cells were transfected with dnPKA, shRNA targeting the PKA catalytic subunit (shPKA), GPKA or the control vectors and maintained for 24 h (GPKA and dnPKA) or 48 h (shPKA). The cells were treated with 20 μM PGE2 or DMSO for 30 min, then exposed to gamma ray irradiation (5 Gy), and harvested after 1 h for western blot analysis. Each column represents the mean ± S.E. of 4–5 separate experiments. An asterisk (*) represents differences showing statistical significance from the irradiated control (*P* ≤ 0.05, Mann–Whitney U test), and double asterisks (**) represent differences showing statistical significance from the PGE2-treated cells (*P* ≤ 0.05, Mann–Whitney U test). Full blots are shown in Supplementary Fig. S26.

To explore the mechanisms that cAMP signaling modulates the DNA-PKcs phosphorylation induced by gamma ray irradiation, the role of PKA, the major effector molecule of cAMP, was examined. When PKA activity was interfered by H-89 treatment or by dominant negative PKA (dnPKA) expression, the PGE2 effect on the phosphorylation at S2056 was abolished following gamma ray irradiation (Fig. 6a,b). Knocking down the PKA catalytic subunit with shPKA also abolished the PGE2 effects on the phosphorylation of S2056 (Fig. 6c). In a similar vein, pretreatment with the PKA-specific agonist 6-phe-cAMP or expression of the exogenous PKA catalytic subunit increased the phosphorylation at S2056 to similar levels to that caused by PGE2 pretreatment (Fig. 6d).

**Figure 7:**
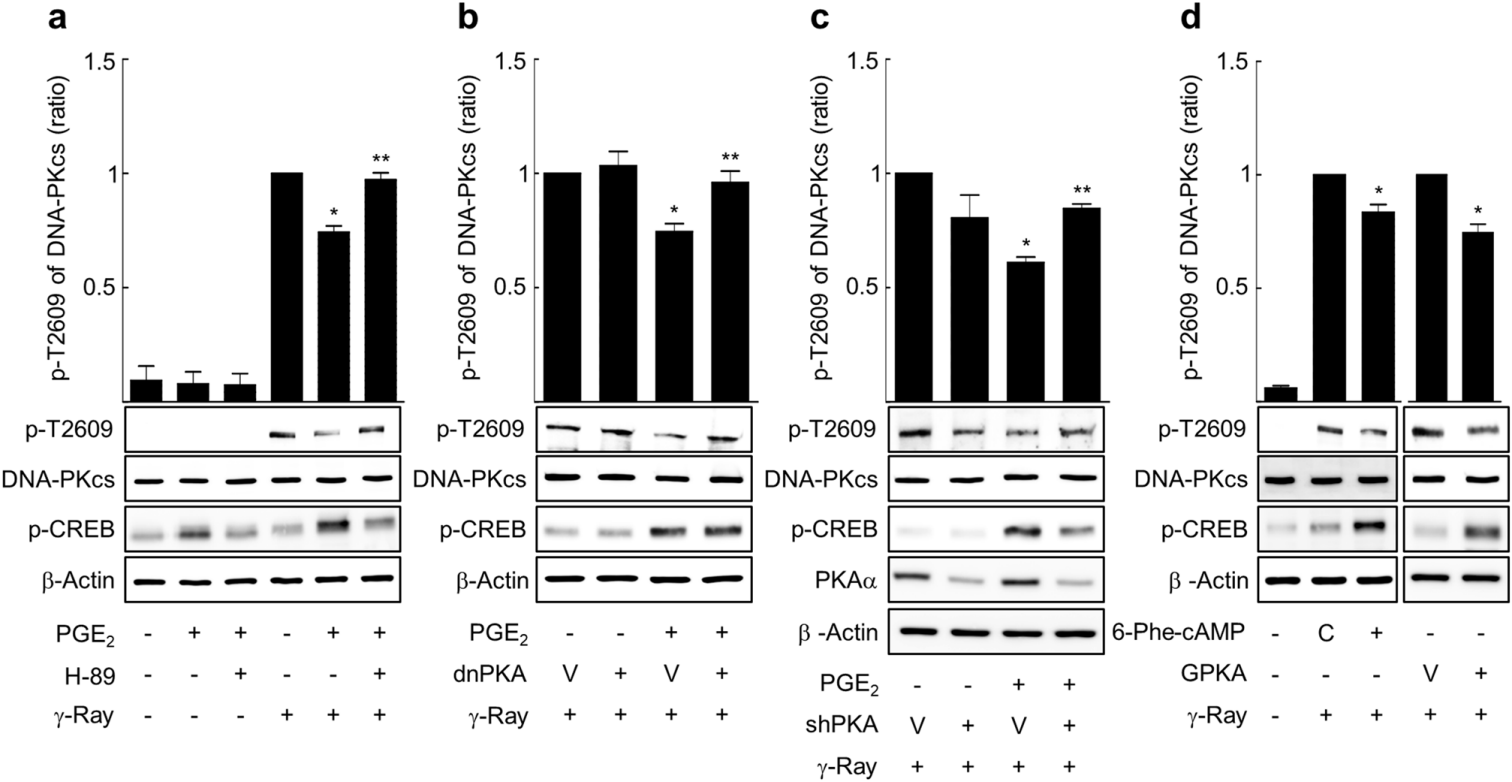
PKA mediated the cAMP effect on phosphorylation at T2609 of DNA-PKcs following gamma ray irradiation. (**a**) Effects of H-89 on phosphorylation at T2609 following gamma ray irradiation (n = 5). (**b**) Effects of dominant negative PKA (dnPKA) on phosphorylation at T2609 following gamma ray irradiation (n = 4). (**c**) Effects of PKA knockdown on the radiation-induced phosphorylation at T2609 following gamma ray irradiation (n = 4). (**d**) Effect of 6-phe-cAMP and PKA catalytic subunit (GPKA) on phosphorylation at T2609 following gamma ray irradiation (n = 4). H1299 cells and H1299 cells treated with 10 μM H-89 (30 min) were reacted with 20 μM PGE2 or 50 μM 6-phe-cAMP (30 min), and then the cells were exposed to gamma irradiation (5 Gy). H1299 NSLC cells were transfected with dnPKA, shRNA targeting the PKA catalytic subunit (shPKA), GPKA or the control vectors and maintained for 24 h (GPKA and dnPKA) or 48 h (shPKA). The cells were treated with 20 μM PGE2 or DMSO for 30 min, then exposed to gamma ray irradiation (5 Gy), and harvested after 1 h for western blot analysis. Each column represents the mean ± S.E. of 4–5 independent experiments. An asterisk (*) represents differences showing statistical significance from the respective control (*P* ≤ 0.05, Mann–Whitney U test), and double asterisks (**) represent differences showing statistical significance from the PGE2-treated cells (*P* ≤ 0.05, Mann–Whitney U test). Full blots are shown in Supplementary Fig. S26.

Comparably, pretreatment with H-89 or dominant negative PKA expression also abated the PGE2 effects on the phosphorylation at T2609 following gamma ray irradiation (Fig. 7a,b). Knocking down the PKA catalytic subunit inhibited the PGE2 effect on T2609 phosphorylation (Fig. 7c). Pretreatment with 6-phe-cAMP or the exogenous expression of PKA decreased the phosphorylation at T2609 (Fig. 7d). The result shows that PKA mediates the modulatory effect of cAMP signaling on the DNA-PKcs phosphorylation at S2056 and T2609 following gamma ray irradiation in H1299 NSLC cells.

### cAMP signaling decreased phosphorylation of T2609 of DNA-PKcs induced by gamma ray irradiation via protein phosphatase 2A (PP2A) and ATM

**Figure 8:**
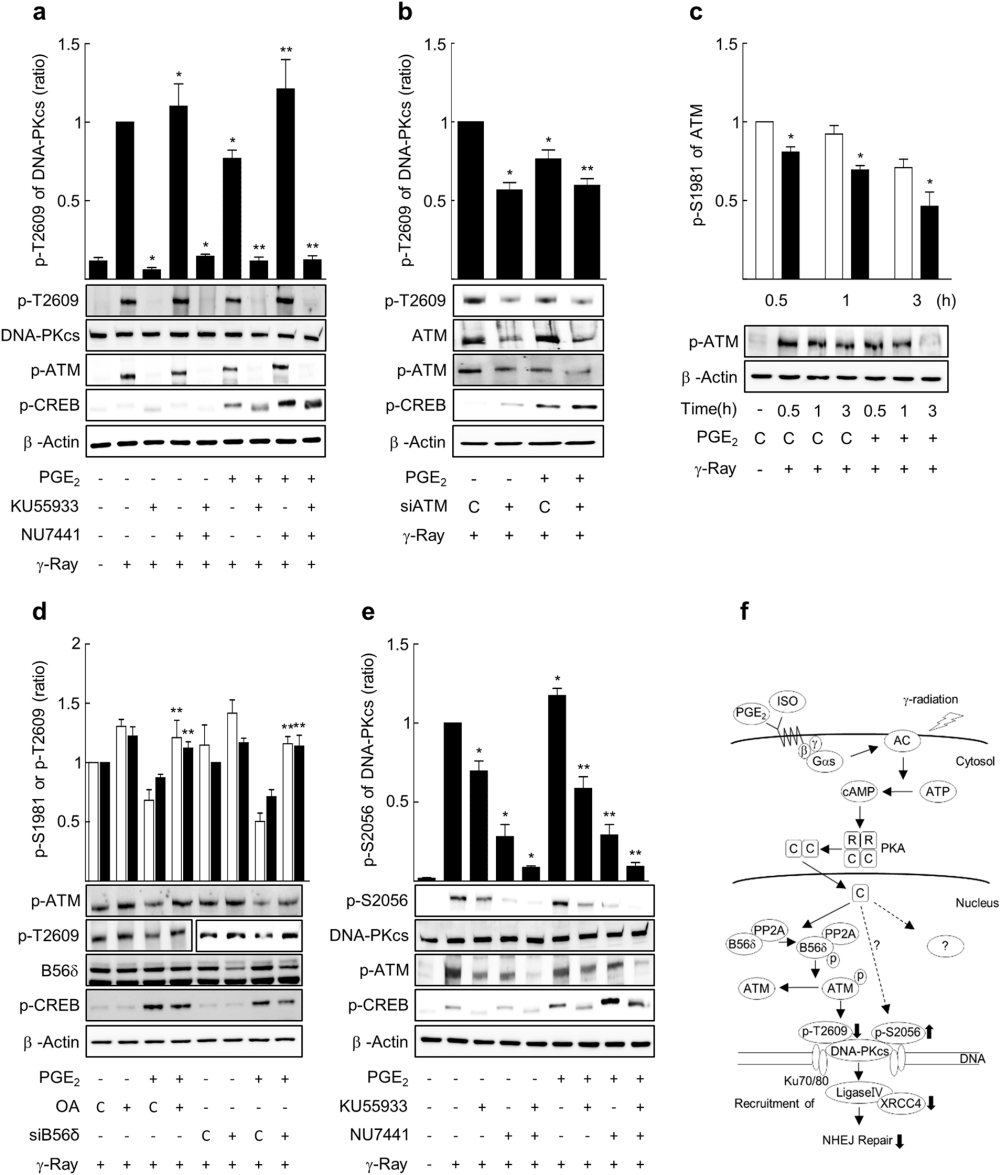
PP2A and ATM mediated the decrease in the phosphorylation of DNA-PKcs at T2609 by cAMP signaling following gamma ray irradiation in H1299 cells. (**a**) Effects of KU55933 and NU7441 on phosphorylation at T2609 following gamma ray irradiation. (**b**) Effects of ATM knockdown on phosphorylation at T2609 following gamma ray irradiation. (**c**) Effects of PGE2 on phosphorylation of ATM at 1981 following gamma ray irradiation. Empty bars represent control cells, and filled bars PGE2-treated cells. (**d**) Effects of okadaic acid (OA) and PP2A B56δ knockdown on the phosphorylation at 1981 of ATM and at T2609 of DNA-PKcs following gamma ray irradiation. The empty bar represents the phosphorylation at 1981, and the filled bar the phosphorylation at T2609. (**e**) Effects of KU55933 and NU7441 on phosphorylation at S2056 following γ-ray irradiation. H1299 cells were treated with 5 μM NU7441, 10 μM KU55933, or 100 nM okadaic acid (30 min) and treated with 20 μM PGE2 (30 min). siRNA (siATM, siB56δ) or a control siRNA were expressed in H1299 cells by transfection and the transfected cells were incubated for 24 h before treatment with 20 μM PGE2. The treated cells were exposed to gamma irradiation (5 Gy) and harvested after 30 min (1 h for S2056) for western blot analysis. Each column represents the mean ± S.E. of four separate experiments. An asterisk (*) represents differences showing statistical significance from the respective control (*P* ≤ 0.05, Mann–Whitney U test), and double asterisks (**) represent differences showing statistical significance from the PGE2-treated cells (*P* ≤ 0.05, Mann–Whitney U test). Full blots are shown in Supplementary Fig. S26. (**f**) A proposed model for the inhibition of NHEJ repair of radiation-induced DSBs by cAMP signaling in lung cancer cells.

Then, to explore the signaling molecules that mediate the decreasing effect of PKA on phosphorylation at T2609, the influences of an ATM inhibitor (KU55933) and a DNA-PK inhibitor (NU7441) were examined. Treatment with KU55933 reduced the radiation-induced phosphorylation of T2609 to near basal levels regardless of PGE2 treatment, but pretreatment with NU7441 slightly increased the phosphorylation of T2609 regardless of PGE2 treatment in H1299 cells (Fig. 8a) and A549 cells (Supplementary Fig. S25). Similarly, knocking down ATM with siATM decreased the phosphorylation at T2609 in H1299 NSLC cells following irradiation (Fig. 8b). Activated cAMP signaling by PGE2 treatment decreased the phosphorylation of ATM at S1981, a marker for ATM activation, suggesting that ATM activation was inhibited by PGE2 treatment (Fig. 8c). Treatment with a PP2A inhibitor (okadaic acid) and knockdown of the B56δ subunit of PP2A using siRNA increased the ATM phosphorylation at S1981 and DNA-PK phosphorylation at T2609 following gamma ray irradiation, and blocked the inhibitory PGE2 effect on phosphorylation at S1981 and T2609, resulting in comparable levels to the control phosphorylation level (Fig. 8d). The results indicate that gamma ray irradiation induces T2609 phosphorylation of DNA-PKcs via ATM and that cAMP signaling decreases the phosphorylation of T2609 following irradiation by the PP2A-dependent inhibition of ATM.

The functions of DNA-PK and ATM in DNA-PK phosphorylation at S2056 after gamma ray irradiation was also examined by inhibiting these enzymes. Blocking DNA-PK with NU7441 eliminated most of radiation-induced phosphorylation at S2056 regardless of whether the cells were pretreated with PGE2 (Fig. 8e). Inhibition of ATM with KU55933 slightly inhibited the phosphorylation of S2056, and the simultaneous inhibition of ATM and DNA-PK totally suppressed phosphorylation at 2056 (Fig. 8e). This result indicates that gamma ray-induced phosphorylation at S2056 is mediated mainly by DNA-PK autophosphorylation and to some extent by ATM in NSLC cells and suggests that cAMP signaling might increase phosphorylation at S2056 in an ATM-independent manner following gamma ray irradiation because ATM activation was inhibited by cAMP signaling in this study.

## Discussion

The present study was carried out to explore the role of cAMP signaling in the repair of DNA DSBs resulted from gamma ray irradiation and its underlying mechanisms in lung cancer cells. It was found that cAMP signaling delays the repair of DNA DSBs resulting from gamma ray irradiation by inhibiting non-homologous end joining (NHEJ) in a PKA-dependent pathway and that cAMP signaling differentially modulates DNA-PKcs phosphorylation at S2056 and at T2609 in PKA-dependent pathways, which might contribute to the inhibition of NHEJ in NSLC cells (Fig. 8f).

cAMP signaling was found to delay the DNA DSB repair following gamma ray irradiation in NSLC cells. This finding is supported by experiments showing that gamma ray-induced DSBs assessed by the neutral comet assay disappeared more slowly following activation of cAMP signaling by expression of GαsQL or by treatments with PGE2 and isoproterenol. Activated cAMP signaling also delayed the disassembly of γ-H2AX foci, known as DNA damage biomarker, following gamma ray irradiation in H1299 and A549 NSLC cells. Moreover, activation of cAMP signaling delayed the repair of I-SceI endonuclease-induced DNA DSBs when the repair was measured by the fluorescence emitted from the repaired reporter plasmid DNA.

The inactivation of PKA function was implicated in enhanced DNA repair and obtainment of resistance to DNA-injuring anticancer drugs in cancer^15^. cAMP signaling was recently reported to decrease Luperox (tert-butyl hydroperoxide, a stable form of hydrogen peroxide)-induced DNA damage measured by γ-H2AX analysis and alkaline comet assay in melanoma cells, implying a stimulatory effect of cAMP signaling in DNA damage repair, including DSB^16^. Our finding agree with this report in that cAMP signaling regulates the repair of DNA DSBs, but the effects on the repair are opposite. In melanoma cells cAMP signaling stimulated repair of Luperox-induced DSBs but in NSLC cells it inhibited the repair of gamma ray-induced DSBs in our study. This difference might result from the differences in the cell types used in the studies, which is similar to the cell type-specificity of cAMP signaling in the regulation of proliferation and apoptosis^17^. Our study shows the inhibitory function of cAMP signaling in the repair of DNA DSBs in H1299 and A549 NSLC cells. Because p53 protein is not produced in H1299 NSLC cells due to a homozygous partial deletion in p53 gene^18^, the inhibitory effect of cAMP seems to be independent to p53 pathway.

The role of cAMP signaling was reported in several DNA repair mechanisms. α-Melanocyte-stimulating hormone stimulates the repair of UVR-induced cyclobutane pyrimidine dimers through stimulating nucleotide excision repair (NER)^19^. cAMP signaling was reported to stimulate NER by phosphorylation of ataxia telangiectasia-mutated and Rad3-related (ATR) in PKA-dependent pathways^20^. Moreover, cAMP signaling was reported to increase the expression of the essential enzymes for BER^13^, and cAMP signaling inhibits the Luperox-induced increase in 7,8-dihydro-8-oxyguanine (8-oxodG), a main product formed in UVR-induced oxidative DNA damage, in melanoma cells^16^, suggesting that cAMP signaling also stimulates BER. Our findings added an important function of cAMP signaling in DNA repair, and suggests, together with previous reports, that cAMP signaling may act substantial roles in various physiological and pathological processes that involve DNA damage repair.

Next, we discovered that the repair of DSBs resulted from gamma ray irradiation was delayed by cAMP signaling through inhibition of NHEJ in NSLC cells. The discovery is grounded in the results that cAMP signaling inhibited the recruitments of XRCC4 and DNA ligase IV, the essential enzymes for NHEJ, to DNA foci in a PKA-dependent manner following gamma ray irradiation. XRCC4 and DNA ligase IV are the essential components for NHEJ together with Ku70, Ku80, and DNA-PKcs. Thus, the PGE2-induced reduction of recruitments of XRCC4 and DNA ligase IV can delay ligation between the damaged DNA strands and therefore delay NHEJ repair. The finding of delay in NHEJ by cAMP signaling is strengthened by the result showing that NHEJ repair of DSBs in plasmid DNA caused by I-SceI endonuclease was inhibited by cAMP signaling in NSLC cells.

Previous papers reported that cAMP signaling affects DNA DSB repair^16^ and that cAMP response element binding protein activated by PKA mediated DNA ligase IV induction in ischemic retinal neurocytes^21^, implying potential regulation of DSB repair, including NHEJ, by cAMP signaling. Nevertheless, there is no published paper that clearly describes the role of cAMP signaling in NHEJ; therefore, this study is, to the best of our knowledge, the first to report clearly that the cAMP signaling system can regulate NHEJ repair of DNA DSBs. NHEJ is the major mechanism of DSB repair acting throughout the cell cycle, and HR is activating during S phase and G2 phase^22^. Thus, inhibition of NHEJ by cAMP signaling may result in the delay of DSB repair. Furthermore, our findings suggest that the extracellular signals that activate cAMP signaling might also regulate DNA DSB repair. The repair of DNA DSBs induced by radiation was stimulated by radiation-induced activation of signaling pathways such as epidermal growth factor receptor and the phosphatidylinositol 3-kinase (PI3K)/protein kinase B (Akt) pathways^23^. Thus, it is speculated that other intracellular signaling pathways stimulated by various external signals might modulate DNA damage repair and the resulting cellular responses such as apoptosis.

To explore the mechanisms of cAMP signaling to delay NHEJ repair, the effects of cAMP signaling was examined on DNA-PK, which is the principal component of NHEJ repair^24^. We found that the phosphorylation at T2609 and S2056 was modulated with opposite directions by cAMP signaling in PKA-dependent pathways following gamma ray exposure. This finding is grounded on the observation that the gamma ray-induced phosphorylation at S2056 of DNA-PKcs was increased, but at T2609, it was decreased by the cAMP signaling activated by exogenous expression of GαsQL and treatment with isoproterenol or PGE2. Furthermore, the modulatory effect of cAMP signaling was abated when PKA activity was inhibited with chemical inhibitor H-89, by knockdown of PKA expression, and by expression of dnPKA. Moreover, stimulation with a PKA-specific activator or overexpression of the PKA catalytic subunit mimicked the modulatory effect of cAMP signaling.

DNA-PKcs is often phosphorylated at numerous sites by DNA-PK itself and other kinases, including ATR and ATM^24^. The proposed functions of the autophosphorylation of DNA-PKcs are regulation of enzyme inactivation, end processing, and complex dissociation^25^. Phosphorylation at the ABCDE and PQR clusters is suggested to have contrary effects upon DNA-PKcs function, and elimination of DNA-PKcs phosphorylation at both regions severely compromises DSB repair and radioresistance^26^. The phosphorylation at the ABCDE region containing T2609 may stimulate DNA-PKcs to dissociate from the DSB ends, thus it promotes the DSB ends to be accessed by subsequent processing and ligation reactions^27^. On the contrary, the phosphorylation in the PQR region containing S2056 protects DNA ends by inhibiting excessive DNA end processing^28,29^, and phosphorylation of S2056 following DNA injury is generally recognized as a trustable marker for DNA-PK activation in cells^8^. Thus, the simultaneous increase in S2056 phosphorylation and decrease in T2609 phosphorylation by cAMP signaling observed in this study might collaboratively inhibit the end access and the DNA-PKcs dissociation from DNA to inhibit NHEJ repair of gamma ray-induced DSBs. The results indicating that cAMP signaling modulates both phosphorylation of DNA-PKcs and recruitments of XRCC4/DNA ligase IV in PKA-dependent pathways supports the hypothesis that cAMP signaling might inhibit NHEJ repair of DSBs by differential modulation of DNA-PKcs phosphorylation in lung cancer cells. In addition, this is an example that shows molecular switch functions of DNA-PKcs which harmonize DNA end processing and DNA end ligation through differential phosphorylation^30^.

PKA is a major effector molecule activated by cAMP signaling together with Epac and cyclic nucleotide-gated ion channels^31^. cAMP signaling was reported to control DNA-PK subcellular localization in two ways: Epac promotes DNA-PK nuclear exit while PKA promotes nuclear entry^32^. A PKA inhibitor suppressed DNA-PK expression in human lymphoblastic leukemia cells^33^, and PKA was required to activate DNA-PK in primary human retinal endothelial cells^34^. In this study, we showed that PKA differentially modulates the phosphorylation at S2056 and T2609 of DNA-PK in NSLC cells.

In a subsequent study to find out the signaling molecules that transmit the cAMP effect downstream of PKA, we found that PP2A and ATM mediate the cAMP-induced decrease in the DNA-PKcs phosphorylation at T2609. The finding is supported by the observations that the phosphorylation at T2609 following irradiation was prevented through ATM inhibition by ATM inhibitor treatment or by ATM knockdown but was not prevented by DNA-PK inhibitor treatment; that treatment with PGE2 decreased the phosphorylation of ATM at Ser1981, a marker for ATM activation; and that PP2A inhibitor treatment or the knockdown of B56δ subunit of PP2A blocked the PGE2 effect on ATM phosphorylation at 1981 and DNA-PK phosphorylation at T2609 following irradiation. These results suggest that PKA stimulate PP2A activity by phosphorylation of the B56δ subunit and then PP2A dephosphorylates ATM to inhibit its activity, which causes a decrease in the DNA-PKcs phosphorylation at T2609. This suggestion is supported by previous paper indicating that ATM is needed for DNA-PKcs phosphorylation at T2609 induced by ionizing radiation^26^, that PP2A forms a complex with ATM and dephosphorylates the autophosphorylated Ser1981 to inhibit ATM activity^35^, and that cAMP signaling decreased ATM phosphorylation at Ser1981 following irradiation by activation of PP2A through the PKA-dependent phosphorylation of the PP2A B56δ subunit^36^. Our finding is consistent with the report that a PP2A-like enzyme is responsible for the reversible protein phosphorylation and activation of DNA-PK^37^ and that PP2A promotes DSB repair by activating the NHEJ pathway^38^. This study also showed that DNA-PK inhibitor treatment slightly increased the phosphorylation of DNA-PK at T2609 following irradiation, suggesting that DNA-PK inhibits the phosphorylation at T2609 following irradiation, which agrees with a report indicating that DNA-PKcs regulates ATM activity negatively by the ATM phosphorylation^39^.

In contrast to the phosphorylation at T2609 of DNA-PKcs, the phosphorylation at S2056 of DNA-PKcs was mostly decreased by DNA-PK inhibitor treatment, but it was also reduced slightly by ATM inhibitor treatment. Thus, the radiation-induced phosphorylation at S2056 of DNA-PKcs seems to be determined mainly by the autophosphorylation of DNA-PK and partially upon ATM. This finding is compatible with a paper reporting autophosphorylation of DNA-PK at S2056 induced by UV and DSBs^40^. However, cAMP signaling inhibited the activation of ATM following gamma ray irradiation in this study, and thus cAMP signaling seems to rely on other signaling molecules to increase the phosphorylation at S2056 of DNA-PKcs in NSLC cells. The signaling molecules that mediate the PKA-dependent increase in phosphorylation at S2056 of DNA-PKcs need to be elucidated in further studies.

In conclusion, this study disclosed that cAMP signaling delays the repair DNA double-strand breaks resulted from gamma ray irradiation by inhibiting NHEJ in NSLC cells and that cAMP signaling differentially modulates phosphorylation at S2056 and T2609 of DNA-PKcs via PKA-dependent pathways, suggesting that cAMP signaling delays DNA DSB repair by inhibiting NHEJ through differential modulation of DNA-PK phosphorylation. These findings imply that cAMP signaling may carry out important functions in DNA DSB repair and thus in the physiological process and the pathogenesis of diseases such as neurological diseases and cancer that involve DNA damage repair. Therefore, it might be possible to prevent the pathogenesis of such diseases or to improve the efficiency of anticancer treatments by modulating cAMP signaling activity.

## Materials and methods

### Cell culture and reagents

Human NSLC cell lines (H1299 and A549) were obtained from Korea Cell Line Bank (Seoul, Korea). H1299 cells were cultivated in Dulbecco’s modified Eagle’s medium, and A549 cells were cultivated in RPMI 1640. Both media were enriched with 10% fetal bovine serum (Welgene, Gyeogsan, Korea) and 100 unit/ml penicillin/streptomycin (Welgene), and the cells were maintained in a 5% CO_2_ incubator at 37 °C. Isoproterenol, H-89, dimethyl sulfoxide (DMSO) and 4′,6′-diamidino-2-phenylindole (DAPI), neocarzinostatin were obtained from Sigma-Aldrich (St. Louis, MO, USA). Prostaglandin E2 (PGE2) was obtained from Cayman Chemical (Ann Arbor, MI, USA), and N6-phenyladenosine-3′,5′-cyclic monophosphate (6-phe-cAMP) was obtained from the Biological Life Science Institute (Bremen, Germany). KU55933 was purchased from Selleckchem (Houston, TX, USA), and NU7441 and okadaic acid were obtained from Tocris Bioscience (Bristol, UK). Bovine serum albumin was obtained from Santa Cruz Biotechnology (Dallas, TX, USA).

### Expression constructs and transient transfection

A long-form of stimulatory GTP-binding protein α subunit (Gαs) that contains constitutively active mutation and a Glu-Glu tag in the pcDNA3.1 + vector (GαsQL) was obtained from Missouri S&T cDNA Resource Center (Rolla, MO, USA). A dominant negative mutant PKA catalytic subunit (dnPKA) was provided by Dr. G. Stanley McKnight (University of Washington, WA, USA). The PKA catalytic subunit (GPKA) with a green fluorescent protein (GFP)-tag was obtained from Dr. Steven H Green (University of Iowa, IA, USA). Fluorescent reporter plasmids for NHEJ were the gift from Dr. Vera Gorbunova (University of Rochester, USA), and the pDsRed-N1 vector was obtained from Dr. MiOk Lee (Seoul National University, Seoul, Korea). Small interfering RNAs (siRNAs) against ataxia telangiectasia mutated (ATM, siATM) and protein phosphatase A (PP2A) B56δ (siB56δ) and control siRNAs were obtained from Santa Cruz Biotechnology. A short hairpin RNA (shRNA) that targets PKA and the control shRNA were bought from Sigma-Aldrich. The expression plasmids and RNAs were transiently transfected using Lipofectamine 3000 (Invitrogen, CA, USA).

### Western blot analysis

Western blot analysis was carried out as described before^41^. Total cell lysates (20–60 μg protein) were separated by SDS–polyacrylamide gel electrophoresis (6–10%). Proteins in the gel were transferred onto nitrocellulose paper, and the transferred proteins were stained with Ponceasu S to trim the NC paper before incubation with specific antibodies. Antibodies against DNA-PKcs, p-CREB (Ser133), α subunit of PKA (PKAα), PP2A B56δ, ATM, and p-ATM (Ser1981) were obtained from Santa Cruz Biotechnology. Antibodies against DNA-PKcs phosphorylated at Ser2056 (S2056) and at Thr2609 (T2609) were acquired from Abcam (Cambridge, UK), and β-actin antibody was bought from Sigma-Aldrich. The proteins were detected by reacting with an enhanced chemiluminescence reagent (Thermo Fisher Scientific, Waltham, MA, USA). The resulting image was recorded using a luminescent image analyzer (LAS-3000, Fuji, Tokyo, Japan). The visualized band density was analyzed by Multi Gauge software v.2.3 (Olympus, Tokyo, Japan), and the analyzed band density was presented as a ratios to the control.

### Assessment of DNA DSBs by single-cell electrophoresis (neutral comet assay)

Cells were seeded in dishes (10 cm in diameter) and cultured to reach 60% confluence. Then, cells were irradiated with gamma rays emitted from a ^137^Cs source with a delivering dose rate of 157.67 cGy/min. The DNA damages in cells were analyzed by neutral comet assay utilizing Trevigen Comet Assay kit (Cat: 4250-050-K, Gaithersburg, MD, USA) following the user manual. In brief, γ-ray-irradiated cells were harvested, mixed with LMAgrose, and placed on slide glass. After 30 min, the attached cells were lysed by incubating with lysis solution for 30 min and then washed with 70% ethanol. The DNA was visualized by incubating with SYBR Safe DNA gel stain (Invitrogen), and the stained DNA images were observed and recorded using a Voice A1 microscope (Nikon, Japan). The extent tail moment was defined as tail DNA% × length of tail, which was calculated using the OpenComet program, a fully automated, free comet assay software^42^.

### Analysis of NHEJ using fluorescent reporters

The DSB repair by NHEJ was analyzed using the NHEJ reporter plasmids, which have a green fluorescence (GFP) gene with the recognition sites for I-SceI endonuclease^43^. The reporter plasmids were linearized by incubating with I-SceI enzyme and transfected into NSLC cells together with the pDsRed-N1 vector for expression control (#632406; Clontech, Mountain View, CA, USA) using Lipofectamine 3000. The cells positive for GFP and positive for pDsRed were counted at the indicated times by flow cytometry (BD LSRFortessa X-20, Becton, Dickinson and Company, Franklin Lakes, NJ, USA). The efficiency of DNA repair was defined as the ratios of green fluorescence to GFP visualized after DNA repair to red fluorescence from the DsRed control.

### Immunofluorescence microscopy

Cells were cultivated on microscope cover glass (Marienfeld, Germany), fixed with 4% paraformaldehyde (10 min), permeabilized in 0.25% Triton X-100 (10 min), and blocked in Dulbecco’s phosphate buffered saline with 1% bovine serum albumin (DPBS) (1 h). The cover glasses were reacted with specific antibodies overnight at 4 °C. The antibodies against γ-H2AX (Ser139, sc-517348), Ku70 (sc-9033), DNA-PKcs (sc-1552), XRCC4 (sc-271087), and DNA-ligase IV (sc-271299) were obtained from Santa Cruz Biotechnology, and the antibody against Ku80 (#2753S) was obtained from Cell Signaling Technology (Beverly, MA, USA). After DPBS wash, the cover glass was reacted with Alexa Fluor 488-conjugated goat anti-mouse secondary antibody, 594-conjugated goat anti-rabbit secondary antibody, or 594-conjugated donkey anti-goat secondary antibody (Invitrogen) for 1 h at room temperature. Next, the cell on the same slides was stained with DAPI (1 μg/ml) in DPBS (10 min). The image of the stained cells was recorded by a confocal microscope (FLUOVIEW FV3000, Olympus, Tokyo, Japan) and analyzed with ImageJ software using macros designed for automatic analysis.

### Statistical analysis

At least 3 independent experiments were performed in all experiments, and the data are expressed as the means and standard errors (S.E). Statistical significance was determined using the nonparametric Mann–Whitney U test, and a *P* value no greater than 0.05 was defined statistically significant.

## Supplementary information

Supplementary information.

## References

[1] Davis AJ, Chen BP, Chen DJ (2014). DNA-PK: a dynamic enzyme in a versatile DSB repair pathway. DNA Repair (Amst.).

[2] Dobbs TA, Tainer JA, Lees-Miller SP (2010). A structural model for regulation of NHEJ by DNA-PKcs autophosphorylation. DNA Repair (Amst.).

[3] Mehta A, Haber JE (2014). Sources of DNA double-strand breaks and models of recombinational DNA repair. Cold Spring Harb. Perspect. Biol..

[4] Jackson SP, Bartek J (2009). The DNA-damage response in human biology and disease. Nature.

[5] Mladenov E, Magin S, Soni A, Iliakis G (2016). DNA double-strand-break repair in higher eukaryotes and its role in genomic instability and cancer: cell cycle and proliferation-dependent regulation. Semin. Cancer Biol..

[6] Tubbs A, Nussenzweig A (2017). Endogenous DNA damage as a source of genomic instability in cancer. Cell.

[7] Pannunzio NR, Watanabe G, Lieber MR (2018). Nonhomologous DNA end-joining for repair of DNA double-strand breaks. J. Biol. Chem..

[8] Goodwin JF, Knudsen KE (2014). Beyond DNA repair: DNA-PK function in cancer. Cancer Discov..

[9] Lefkimmiatis K, Zaccolo M (2014). cAMP signaling in subcellular compartments. Pharmacol. Ther..

[10] Gold MG, Gonen T, Scott JD (2013). Local cAMP signaling in disease at a glance. J. Cell Sci..

[11] Hussain M, Tang F, Liu J, Zhang J, Javeed A (2015). Dichotomous role of protein kinase A type I (PKAI) in the tumor microenvironment: a potential target for 'two-in-one' cancer chemoimmunotherapeutics. Cancer Lett..

[12] Cho EA, Juhnn YS (2012). The cAMP signaling system inhibits the repair of gamma-ray-induced DNA damage by promoting Epac1-mediated proteasomal degradation of XRCC1 protein in human lung cancer cells. Biochem. Biophys. Res. Commun..

[13] Kadekaro AL (2012). Alpha-melanocyte-stimulating hormone suppresses oxidative stress through a p53-mediated signaling pathway in human melanocytes. Mol. Cancer Res..

[14] Jarrett SG, Wolf Horrell EM, D’Orazio JA (2016). AKAP12 mediates PKA-induced phosphorylation of ATR to enhance nucleotide excision repair. Nucleic Acids Res..

[15] Liu B, Cvijic ME, Jetzt A, Chin KV (1996). Cisplatin resistance and regulation of DNA repair in cAMP-dependent protein kinase mutants. Cell Growth Differ..

[16] Castejon-Grinan M, Herraiz C, Olivares C, Jimenez-Cervantes C, Garcia-Borron JC (2018). cAMP-independent non-pigmentary actions of variant melanocortin 1 receptor: AKT-mediated activation of protective responses to oxidative DNA damage. Oncogene.

[17] Ladilov Y, Appukuttan A (2014). Role of soluble adenylyl cyclase in cell death and growth. Biochim. Biophys. Acta.

[18] Mitsudomi T (1992). p53 gene mutations in non-small-cell lung cancer cell lines and their correlation with the presence of ras mutations and clinical features. Oncogene.

[19] Kadekaro AL (2005). alpha-Melanocortin and endothelin-1 activate antiapoptotic pathways and reduce DNA damage in human melanocytes. Cancer Res..

[20] Jarrett SG (2014). PKA-mediated phosphorylation of ATR promotes recruitment of XPA to UV-induced DNA damage. Mol. Cell.

[21] Yang Y (2016). Lithium promotes DNA stability and survival of ischemic retinal neurocytes by upregulating DNA ligase IV. Cell Death Dis..

[22] Iliakis G (2004). Mechanisms of DNA double strand break repair and chromosome aberration formation. Cytogenet. Genome Res..

[23] Toulany M (2019). Targeting DNA double-strand break repair pathways to improve radiotherapy response. Genes.

[24] Jette N, Lees-Miller SP (2015). The DNA-dependent protein kinase: a multifunctional protein kinase with roles in DNA double strand break repair and mitosis. Prog. Biophys. Mol. Biol..

[25] Neal JA, Meek K (2011). Choosing the right path: does DNA-PK help make the decision?. Mutat. Res..

[26] Chen BP (2007). Ataxia telangiectasia mutated (ATM) is essential for DNA-PKcs phosphorylations at the Thr-2609 cluster upon DNA double strand break. J. Biol. Chem..

[27] Ding Q (2003). Autophosphorylation of the catalytic subunit of the DNA-dependent protein kinase is required for efficient end processing during DNA double-strand break repair. Mol. Cell Biol..

[28] Chen BP (2005). Cell cycle dependence of DNA-dependent protein kinase phosphorylation in response to DNA double strand breaks. J. Biol. Chem..

[29] Cui X (2005). Autophosphorylation of DNA-dependent protein kinase regulates DNA end processing and may also alter double-strand break repair pathway choice. Mol. Cell Biol..

[30] Jiang W (2015). Differential phosphorylation of DNA-PKcs regulates the interplay between end-processing and end-ligation during nonhomologous end-joining. Mol. Cell.

[31] Rodriguez CI, Setaluri V (2014). Cyclic AMP (cAMP) signaling in melanocytes and melanoma. Arch. Biochem. Biophys..

[32] Huston E (2008). EPAC and PKA allow cAMP dual control over DNA-PK nuclear translocation. Proc. Natl. Acad. Sci. U. S. A..

[33] Ahnesorg P, Smith P, Jackson SP (2006). XLF interacts with the XRCC4-DNA ligase IV complex to promote DNA nonhomologous end-joining. Cell.

[34] Zhang Q, Steinle JJ (2013). DNA-PK phosphorylation of IGFBP-3 is required to prevent apoptosis in retinal endothelial cells cultured in high glucose. Invest. Ophthalmol. Vis. Sci..

[35] Goodarzi AA (2004). Autophosphorylation of ataxia-telangiectasia mutated is regulated by protein phosphatase 2A. EMBO J..

[36] Cho EA, Kim EJ, Kwak SJ, Juhnn YS (2014). cAMP signaling inhibits radiation-induced ATM phosphorylation leading to the augmentation of apoptosis in human lung cancer cells. Mol. Cancer.

[37] Douglas P, Moorhead GB, Ye R, Lees-Miller SP (2001). Protein phosphatases regulate DNA-dependent protein kinase activity. J. Biol. Chem..

[38] Wang Q, Gao F, Wang T, Flagg T, Deng X (2009). A nonhomologous end-joining pathway is required for protein phosphatase 2A promotion of DNA double-strand break repair. Neoplasia.

[39] Zhou Y (2017). Regulation of the DNA damage response by DNA-PKcs inhibitory phosphorylation of ATM. Mol. Cell.

[40] Meek K, Douglas P, Cui X, Ding Q, Lees-Miller SP (2007). trans Autophosphorylation at DNA-dependent protein kinase's two major autophosphorylation site clusters facilitates end processing but not end joining. Mol. Cell Biol..

[41] Seo M (2004). Cdc42-dependent mediation of UV-induced p38 activation by G protein betagamma subunits. J. Biol. Chem..

[42] Gyori BM, Venkatachalam G, Thiagarajan PS, Hsu D, Clement MV (2014). OpenComet: an automated tool for comet assay image analysis. Redox Biol..

[43] Seluanov A, Mittelman D, Pereira-Smith OM, Wilson JH, Gorbunova V (2004). DNA end joining becomes less efficient and more error-prone during cellular senescence. Proc. Natl. Acad. Sci. U. S. A..

