# Supplementary material for: Inhibition of non-homologous end joining of gamma ray-induced DNA double-strand breaks by cAMP signaling in lung cancer cells
Source: Sci Rep. 2020 Sep 2;10:14455. doi: 10.1038/s41598-020-71522-9 (PMC7468279; doi:10.1038/s41598-020-71522-9)

Supplementary Figures

Inhibition of non-homologous end joining of gamma ray-induced DNA double-strand breaks by cAMP signaling in lung cancer cells

Sung-Eun Noh[1] and Yong-Sung Juhnn[1]

[1]Department of Biochemistry and Molecular Biology, Department of Biomedical Sciences, and Cancer Research Institute, Seoul National University College of Medicine, Seoul, Korea.

Supplementary Figure S1

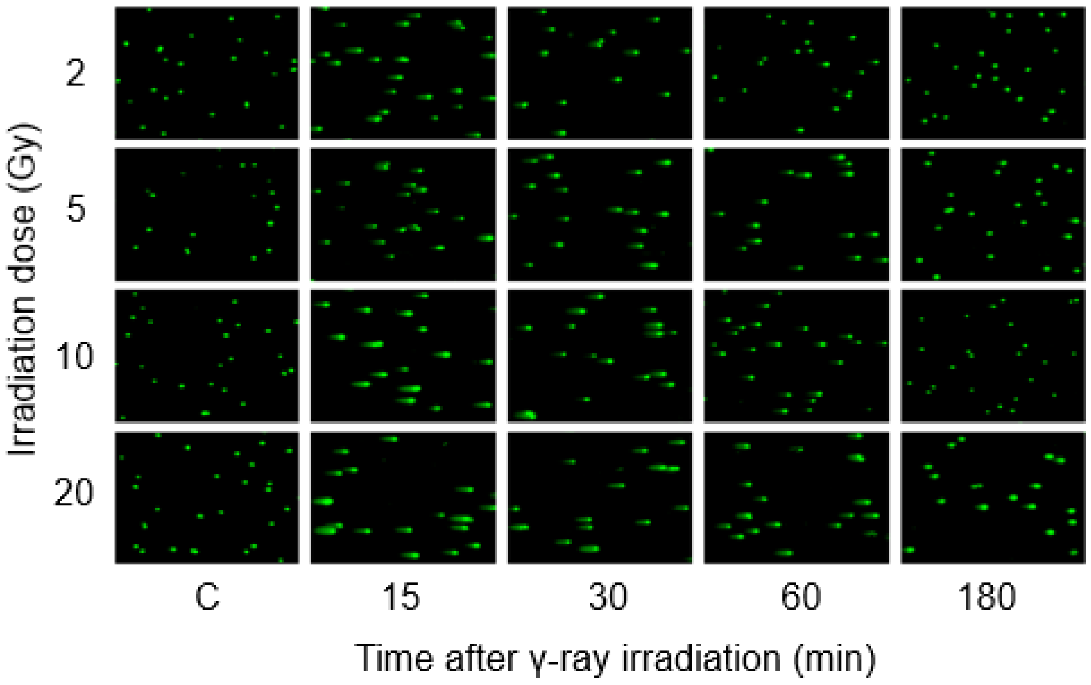

Supplementary Figure S1. **Temporal patterns of DNA DSBs induced by various irradiation doses of γ-ray.** H1299 non-small cell lung cancer cells were irradiated with various doses of γ-ray (2, 5, 10, 20 Gy), and the resulting DNA damage was assessed by neutral comet assay at the indicated times. DNA was stained with SYBR Safe DNA Gel Stain, and the stained DNA image was recorded using a confocal microscope. The extent tail moment was defined as Tail DNA% x Length of Tail and calculated using the OpenComet program.

Supplementary Figure S2

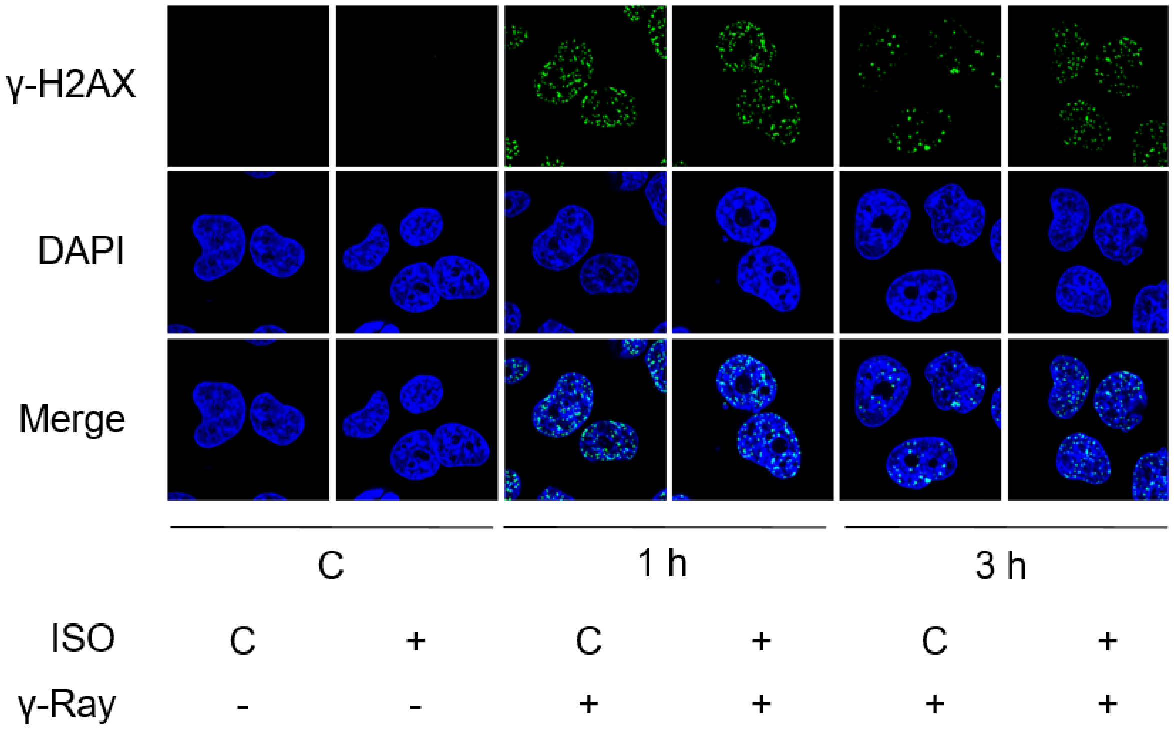

Supplementary Figure S2. Effects of isoproterenol on DNA DSBs following γ-ray irradiation in H1299 cells. H1299 cells pretreated with 1 μM isoproterenol (ISO) for 30 min were irradiated with γ-ray (5 Gy). The irradiated cells were stained with an anti-γ-H2AX antibody and DAPI. γ-H2AX is shown as green, and DAPI is shown as blue.

Supplementary Figure S3

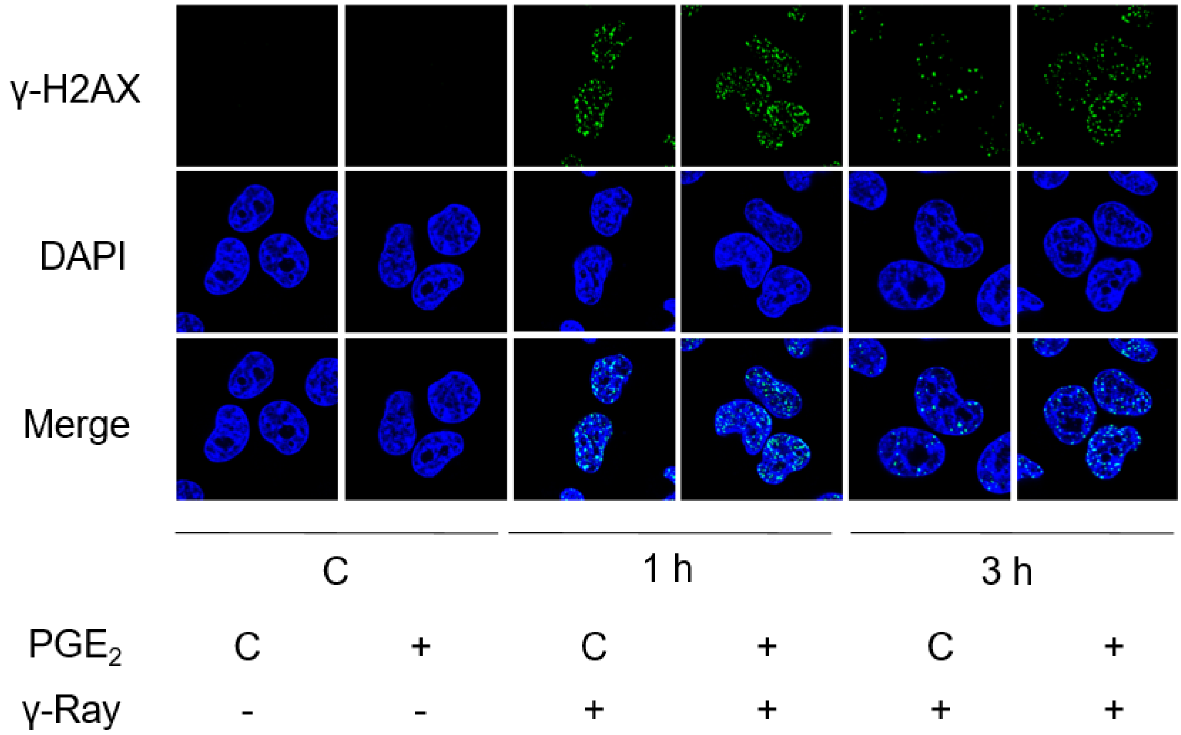

Supplementary Figure S3. Effects of PGE2 on DNA DSBs following γ-ray irradiation in H1299 cells. H1299 cells pretreated with 20 μM PGE2 for 30 min were irradiated with γ-ray (5 Gy). The irradiated cells were stained with an anti-γ-H2AX antibody and DAPI. γ-H2AX is shown as green, and DAPI is shown as blue.

Supplementary Figure S4

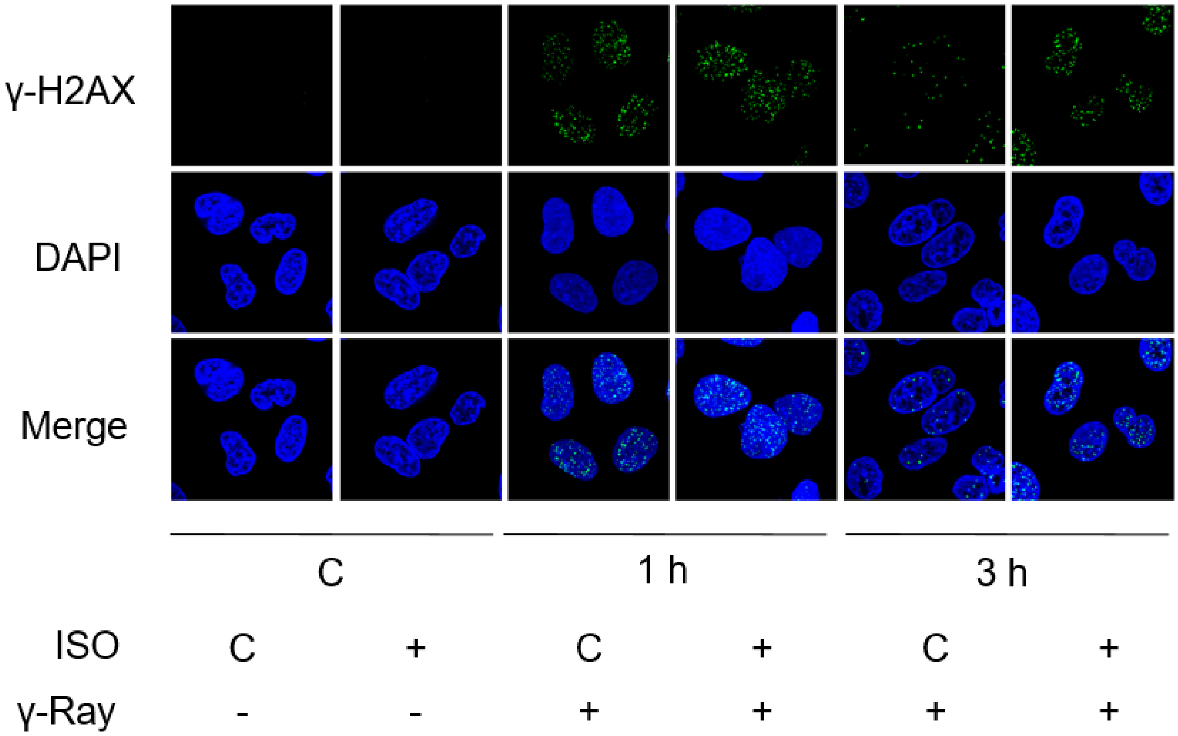

Supplementary Figure S4. **Effects of isoproterenol on DNA DSBs following γ-ray irradiation in A549 cells.** A549 non-small cell lung cancer cells pretreated with 1 μM isoproterenol (ISO) for 30 min were irradiated with γ-rays (5 Gy). The irradiated cells were stained with an anti-γ-H2AX antibody and DAPI. γ-H2AX is shown as green, and DAPI is shown as blue.

Supplementary Figure S5

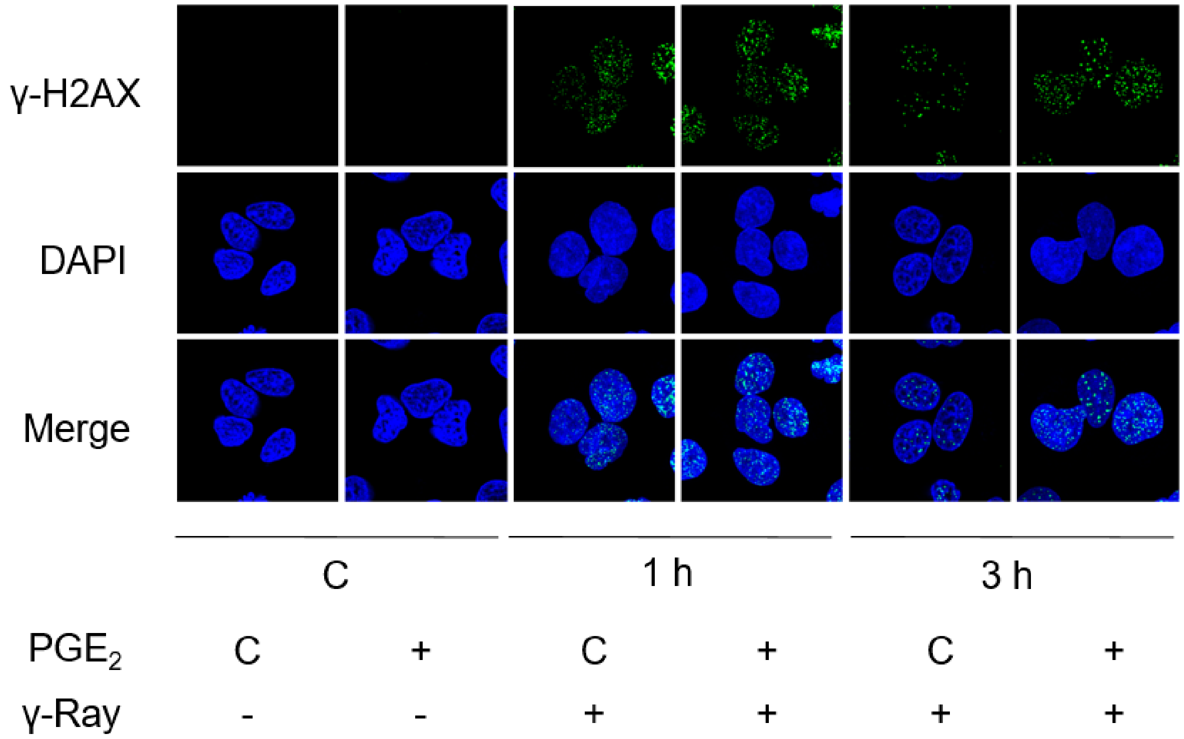

Supplementary Figure S5. Effects of PGE_2 on DNA DSBs following γ-ray irradiation in A549 cells. A549 non-small cell lung cancer cells pretreated with 20 μM PGE_2 for 30 min were irradiated with γ-rays (5 Gy). The irradiated cells were stained with an anti-γ-H2AX antibody and DAPI. γ-H2AX is shown as green, and DAPI is shown as blue.

Supplementary Figure S6

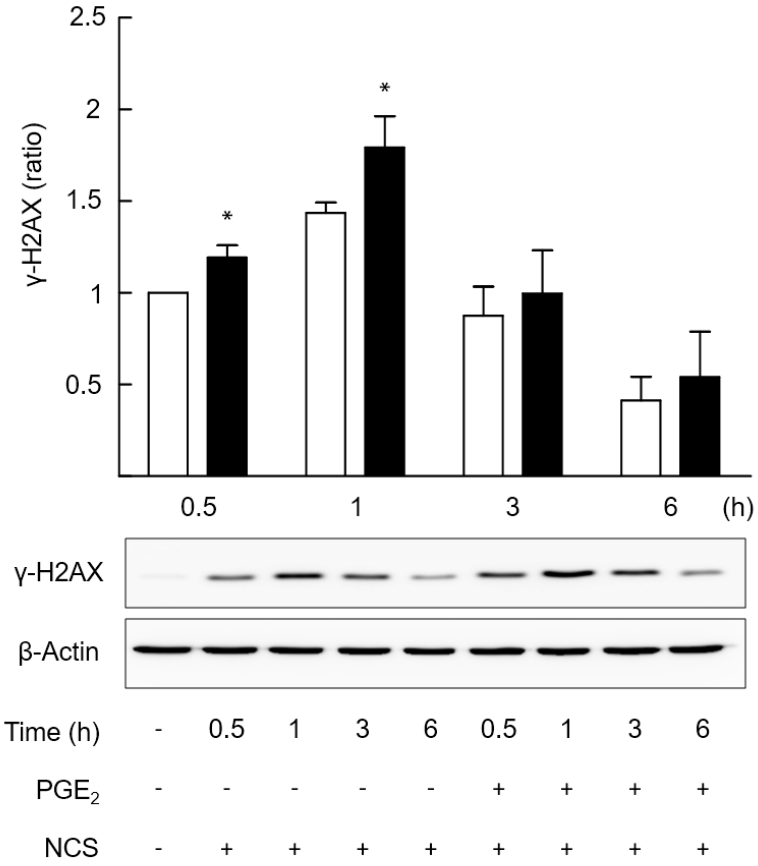

Supplementary Figure S6. Effects of PGE_2 on DNA DSBs resulting from neocarzinostatin (NCS) treatment in H1299 cells. The cells were pretreated with 20 μM PGE_2 for 30 min, then incubated with 500 nM NCS, and harvested at the indicated times for western blot analysis. Empty bars represent control cells, and filled bars represent PGE_2 treated (n=3).

Supplementary Figure S7

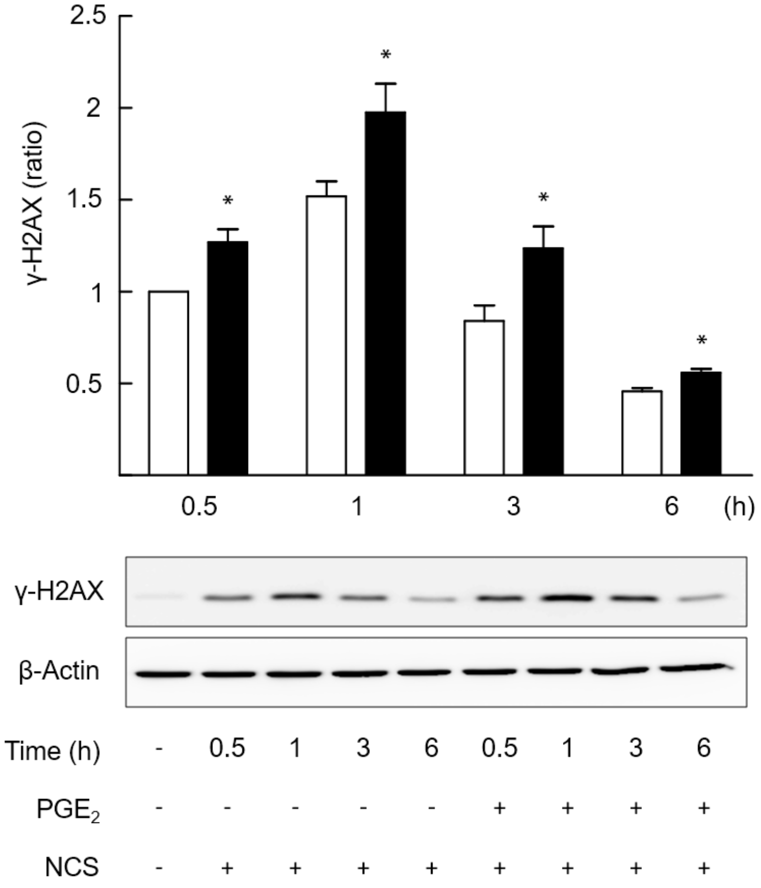

Supplementary Figure S7. Effects of PGE_2 on DNA DSBs resulting from neocarzinostatin (NCS) treatment in A549 cells. The cells were pretreated with 20 μM PGE_2 for 30 min, then incubated with 500 nM NCS, and harvested at the indicated times for western blot analysis. Empty bars represent control cells, and filled bars represent PGE_2 treated (n=3).

Supplementary Figure S8

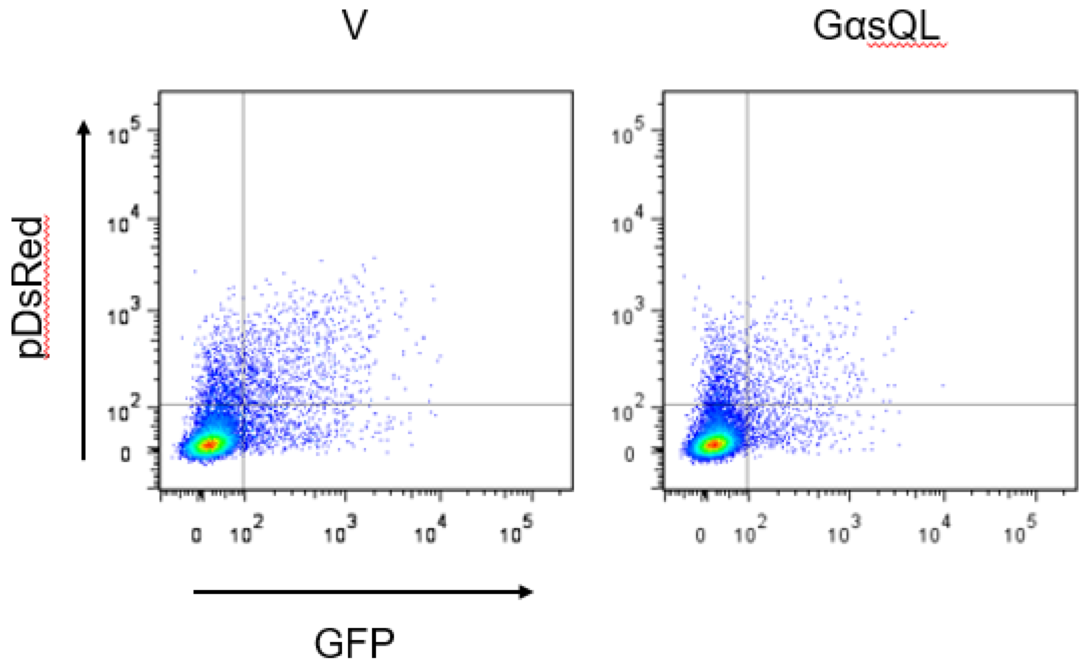

Supplementary Figure S8. **Effects of GαsQL on NHEJ repair of DSBs induced by I-SceI endonuclease.** H1299 cells were transfected with the linearized NHEJ reporter, pDsRed-N2, and GαsQL using Lipofectamine 3000. The transfected cells were harvested after 48 h, and the fluorescence was analyzed by flow cytometry.

Supplementary Figure S9

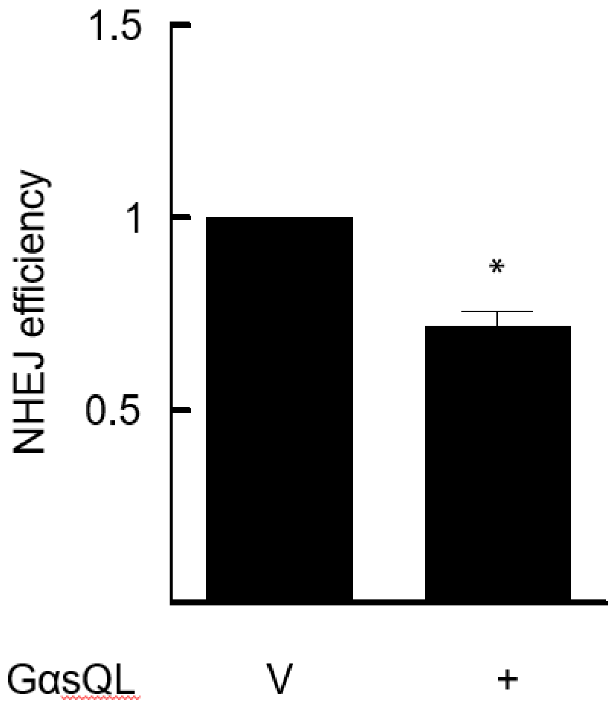

Supplementary Figure S9. Effects of GαsQL on NHEJ repair of DSBs induced by I-SceI endonuclease in A549 cells. A549 cells were transfected with the linearized NHEJ reporter, pDsRed-N2, and GαsQL using Lipofectamine 3000. The transfected cells were harvested after 48 h, and the fluorescence was analyzed by flow cytometry.

Supplementary Figure S10

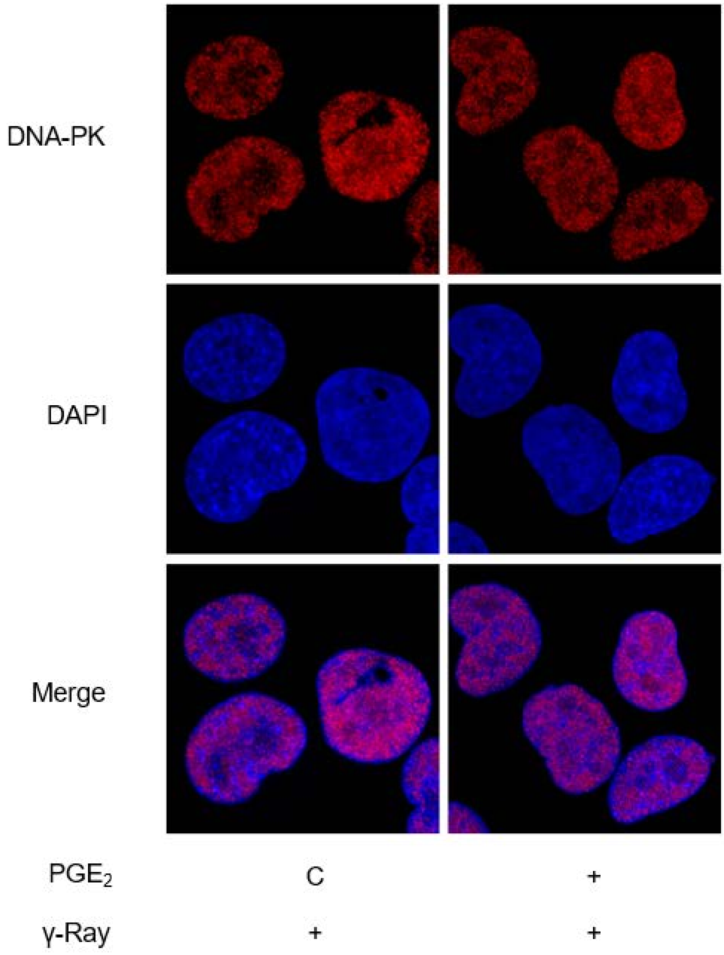

Supplementary Figure S10. Effects of PGE2 on the recruitment of DNA-PKcs following γ-ray irradiation. H1299 cells were treated with 20 μM PGE2 for 30 min, and then irradiated with γ-rays (5 Gy). The irradiated cells were harvested after 1 h and stained with DAPI (blue) and an antibody against DNA-PKcs (red). The images of the stained cells were acquired using a confocal microscope.

Supplementary Figure S11

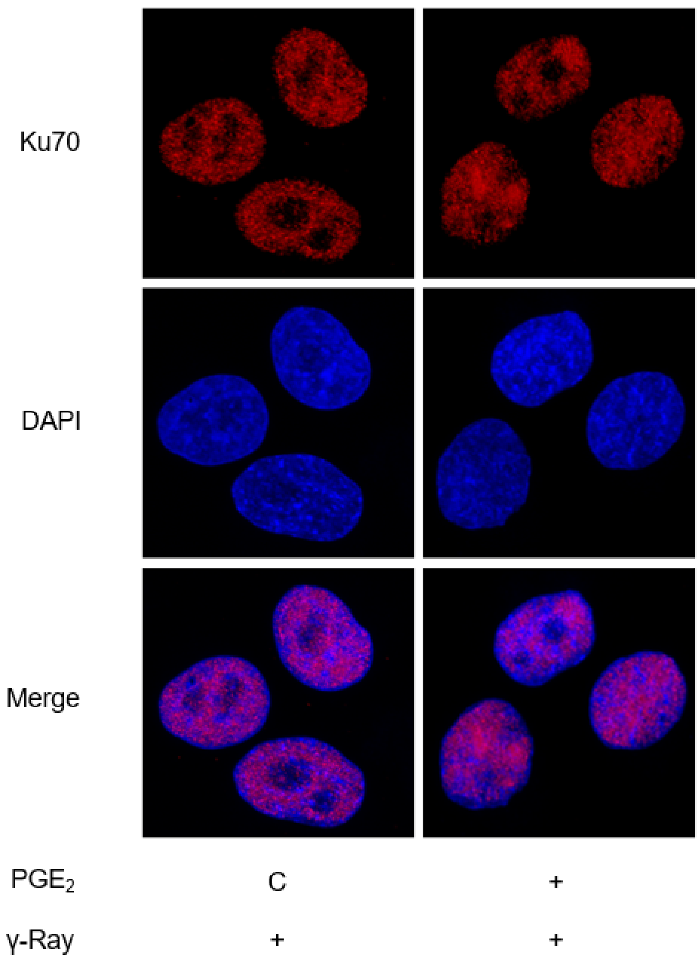

Supplementary Figure S11. Effects of PGE2 on the recruitment of Ku70 following γ-ray irradiation. H1299 cells were treated with 20 μM PGE2 for 30 min, and then irradiated with γ-rays (5 Gy). The irradiated cells were harvested after 1 h and stained with DAPI (blue) and an antibody against Ku70 (red). The images of the stained cells were acquired using a confocal microscope.

Supplementary Figure S12

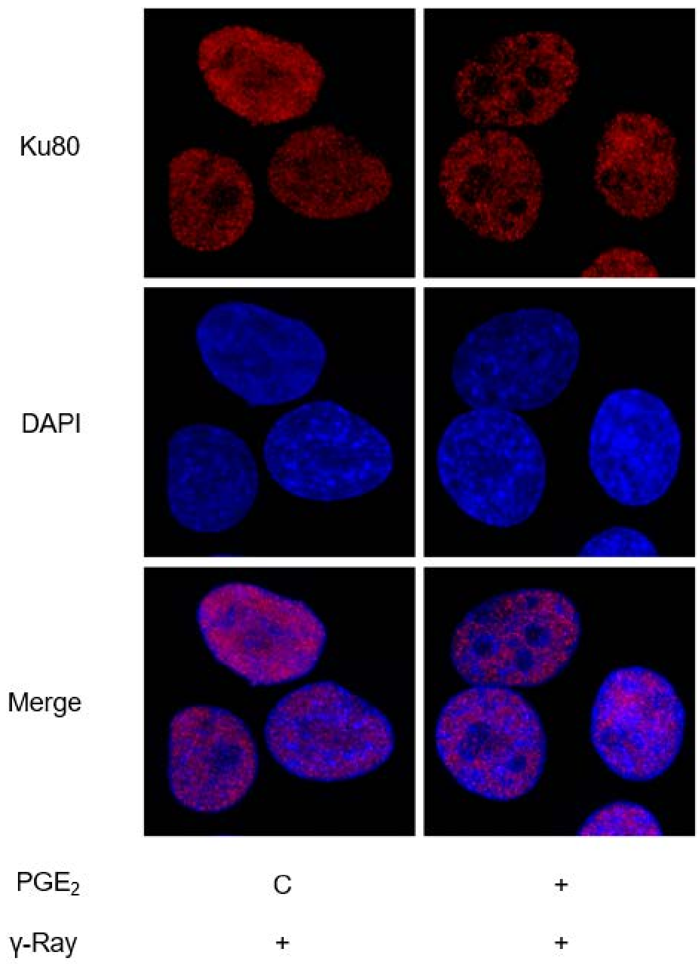

Supplementary Figure S12. Effects of PGE2 on the recruitment of Ku80 following γ-ray irradiation. H1299 cells were treated with 20 μM PGE2 for 30 min, and then irradiated with γ-rays (5 Gy). The irradiated cells were harvested after 1 h and stained with DAPI (blue) and an antibody against Ku80 (red). The images of the stained cells were acquired using a confocal microscope.

Supplementary Figure 13

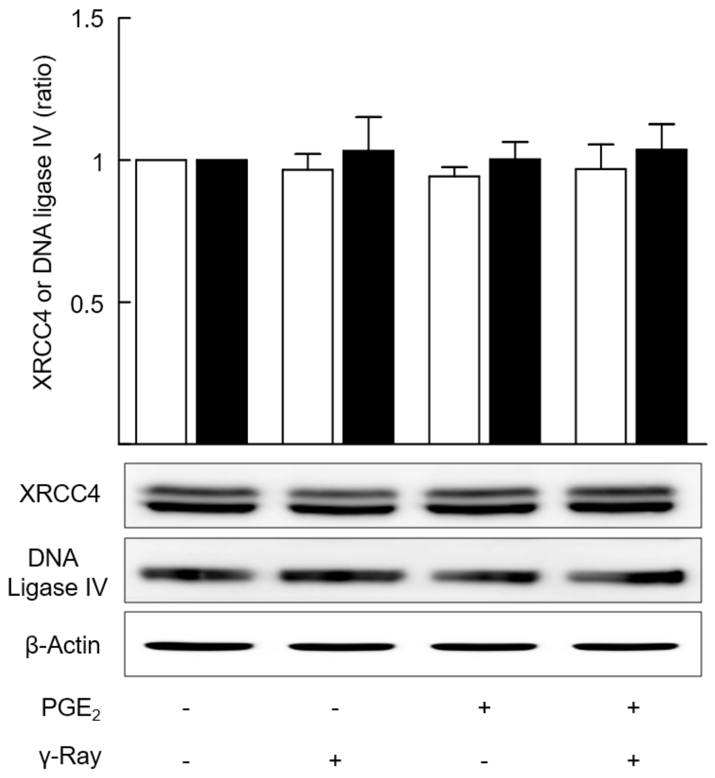

Supplementary Figure S13. Effects of PGE2 on the expression of XRCC4 and DNA-ligase IV following gamma ray irradiation in H1299 cells. The cells pretreated with 20 μM PGE2 for 30 min were exposed to gamma rays (5 Gy) and harvested after 1 h for western blot analysis. Empty bars represent XRCC4, filled bars represent DNA-ligase IV (n=3).

Supplementary Figure S14

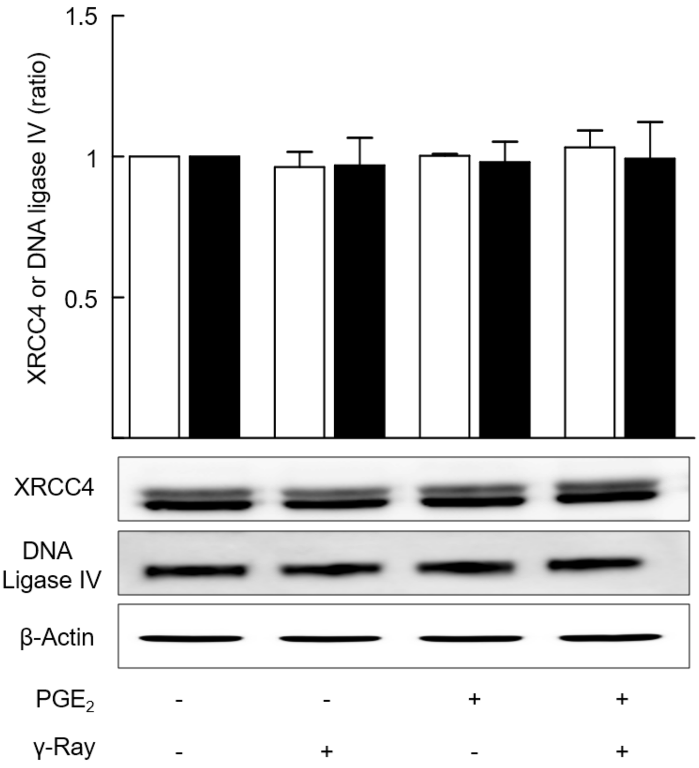

Supplementary Figure S14. Effects of PGE2 on the expression of XRCC4 and DNA-ligase IV following gamma ray irradiation in A549 cells. The cells pretreated with 20 μM PGE2 for 30 min were exposed to gamma rays (5 Gy) and harvested after 1 h for western blot analysis. Empty bars represent XRCC4, filled bars represent DNA-ligase IV (n=3).

Supplementary Figure S15

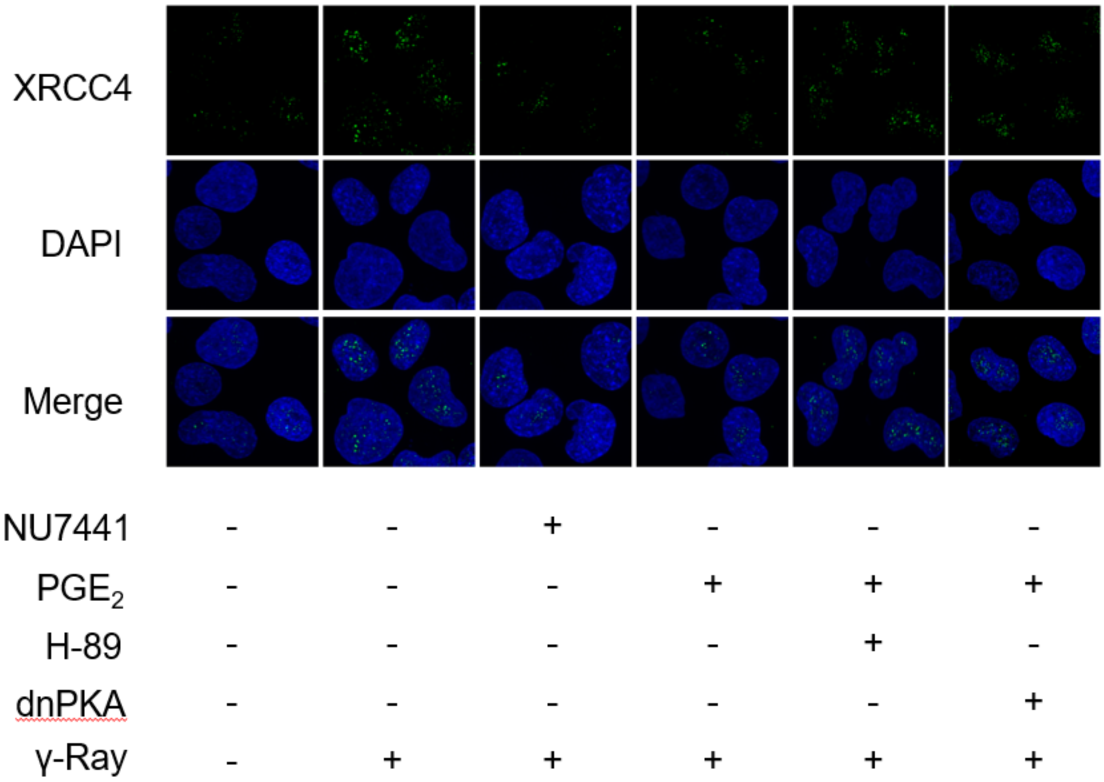

Supplementary Figure S15. Effects of NU7441, H-89, and dnPKA on recruitment of XRCC4 following γ-ray irradiation in H1299 cells. H1299 cells transfected with dominant negative PKA (dnPKA) and H1299 cells pretreated with 10 μM H-89 for 30 min were treated with 20 μM PGE2 or 5 μM NU7441 for 30 min, and then irradiated with γ-rays (5 Gy). The irradiated cells were harvested after 1h, and stained with DAPI (blue) and an antibody against XRCC4 (green). The images of stained cells were acquired using a confocal microscope.

Supplementary Figure S16

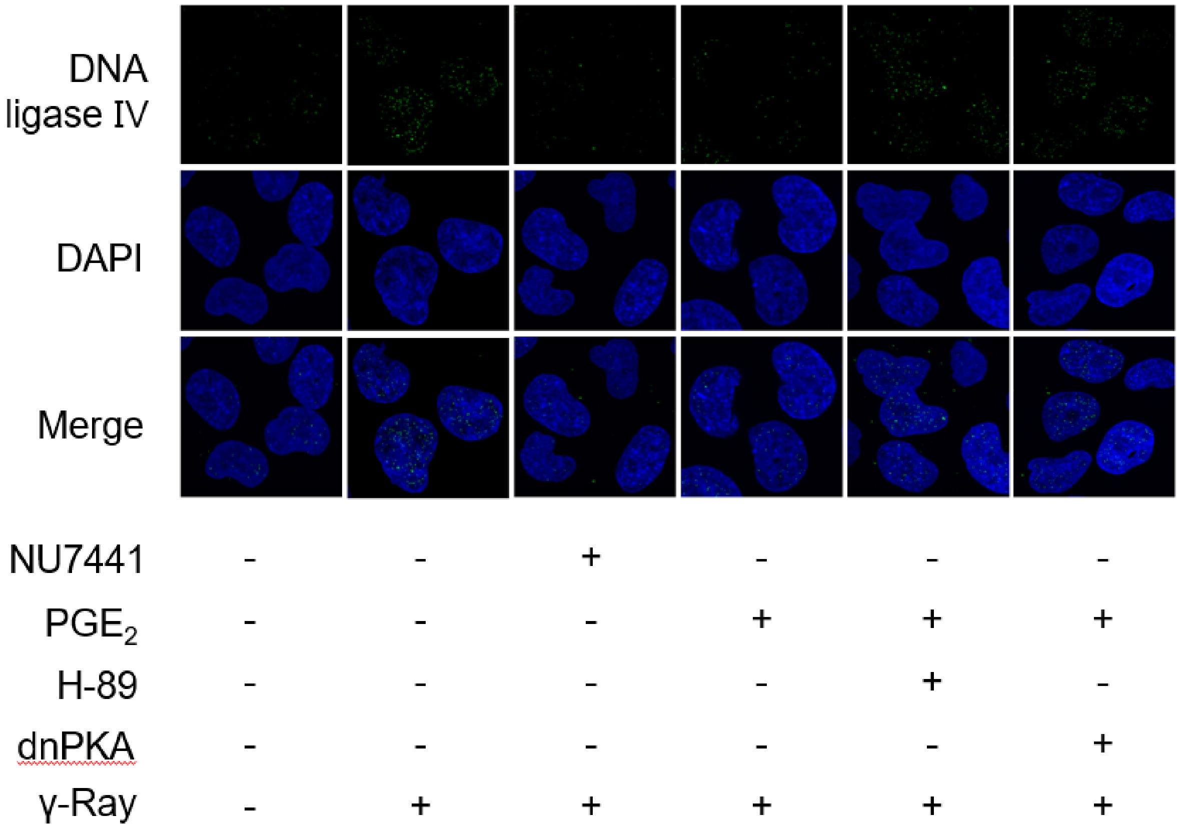

Supplementary Figure S16. Effects of NU7441, H-89, and dnPKA on recruitment of DNA ligase IV following γ-ray irradiation in H1299 cells. H1299 cells transfected with dominant negative PKA (dnPKA) and H1299 cells pretreated with 10 μM H-89 for 30 min were treated with 20 μM PGE2 or 5 μM NU7441 for 30 min, and then irradiated with γ-rays (5 Gy). The irradiated cells were harvested after 1h, and stained with DAPI (blue) and an antibody against DNA ligase IV (green). The images of stained cells were acquired using a confocal microscope.

Supplementary Figure S17

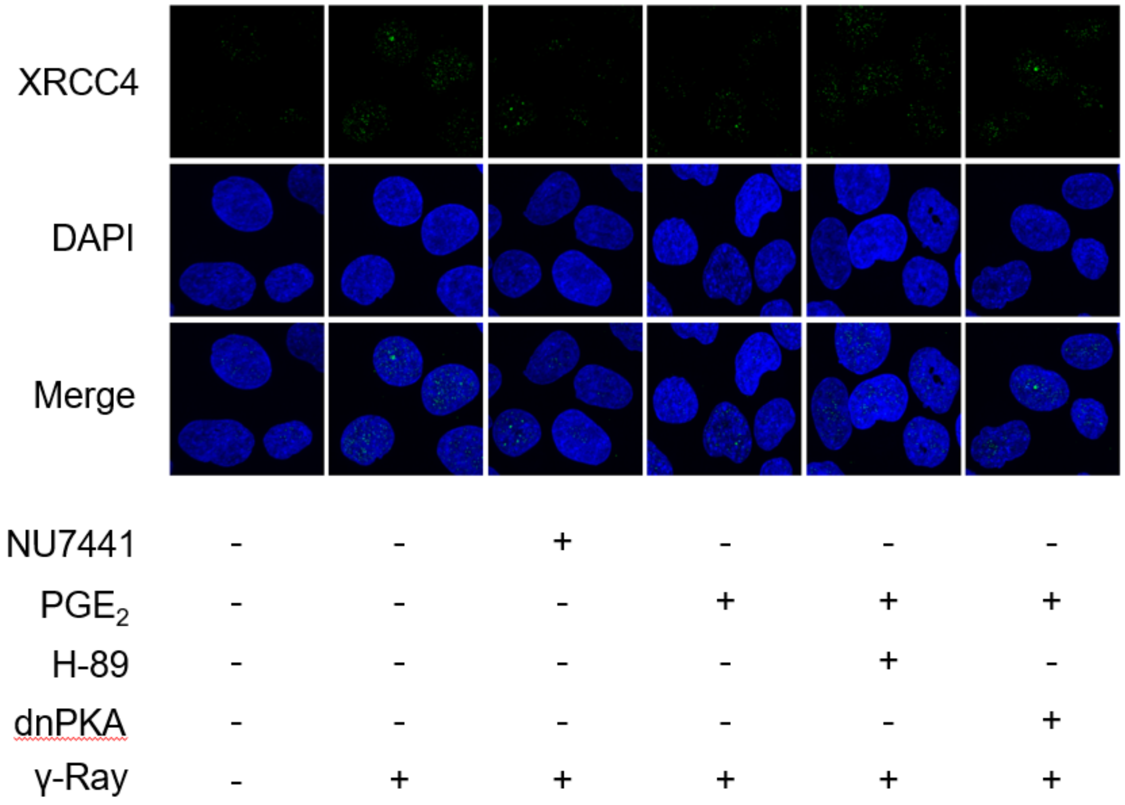

Supplementary Figure S17. Effects of NU7441, H-89, and dnPKA on recruitment of XRCC4 following γ-ray irradiation in A549 cells. A549 cells transfected with dominant negative PKA (dnPKA) and A549 cells pretreated with 10 μM H-89 for 30 min were treated with 20 μM PGE2 or 5 μM NU7441 for 30 min, and then irradiated with γ-rays (5 Gy). The irradiated cells were harvested after 1h, and stained with DAPI (blue) and an antibody against XRCC4 (green). The images of stained cells were acquired using a confocal microscope.

Supplementary Figure S18

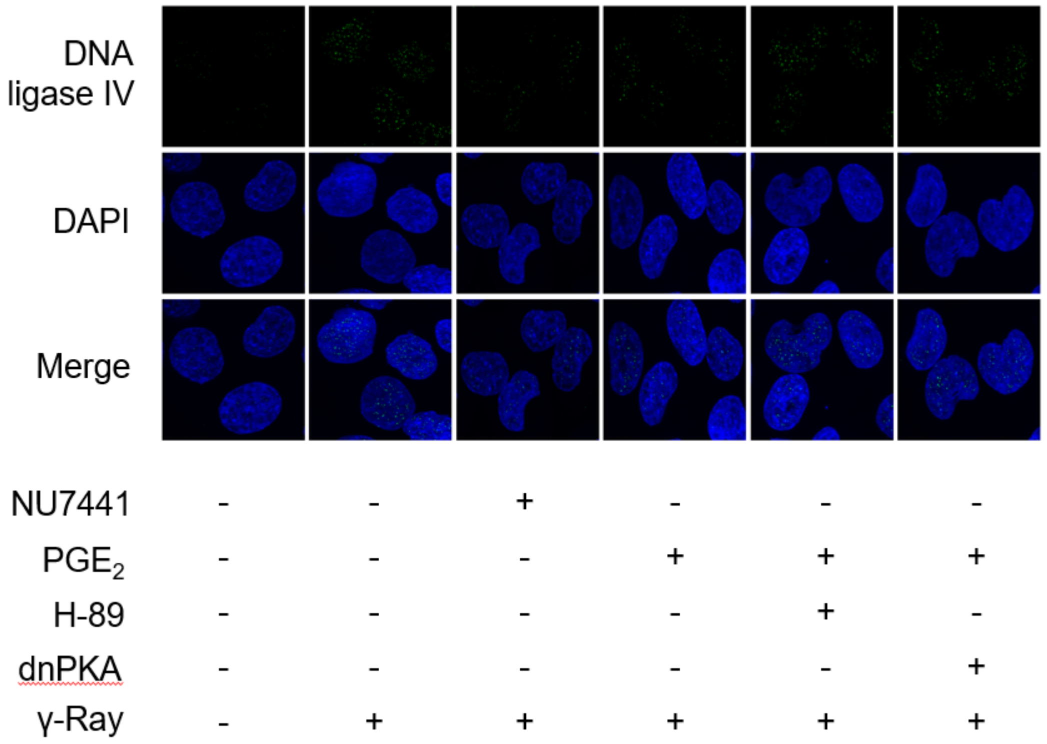

Supplementary Figure S18. Effects of NU7441, H-89, and dnPKA on recruitment of DNA ligase IV following γ-ray irradiation in A549 cells. A549 cells transfected with dominant negative PKA (dnPKA) and A549 cells pretreated with 10 μM H-89 for 30 min were treated with 20 μM PGE2 or 5 μM NU7441 for 30 min, and then irradiated with γ-rays (5 Gy). The irradiated cells were harvested after 1h, and stained with DAPI (blue) and an antibody against DNA ligase IV (green). The images of stained cells were acquired using a confocal microscope.

Supplementary Figure S19

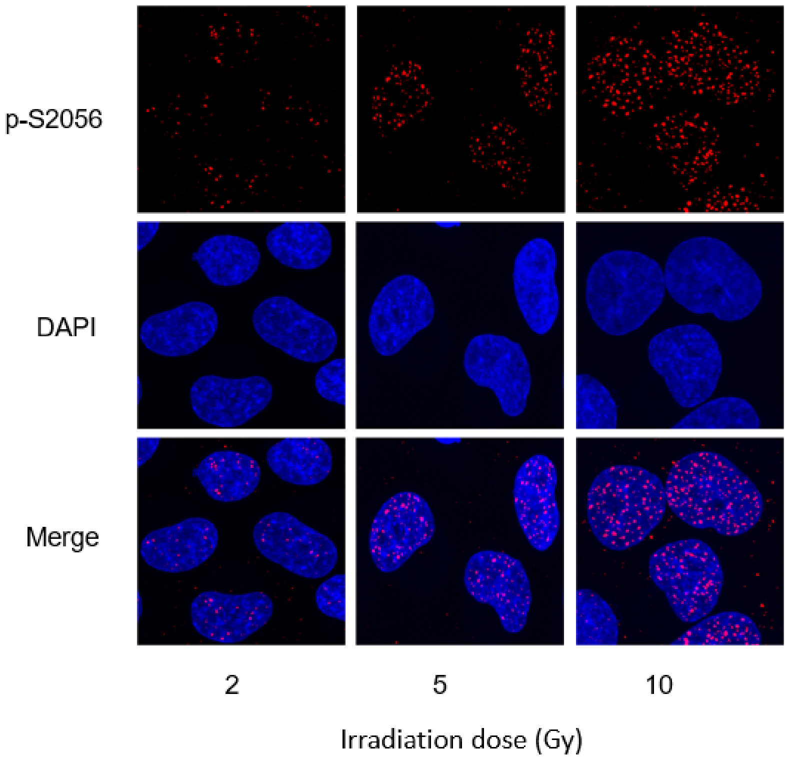

Supplementary Figure S19. Effects of the irradiation dose of γ-ray on the phosphorylation of DNA-PKcs at S2056. H1299 cells on microscope cover glasses were irradiated with different doses of γ-ray (2, 5, 10 Gy), and harvested after 1 h for staining with an antibody against p-S2056 and with DAPI. The stained cells were analyzed by confocal microscope.

Supplementary Figure S20

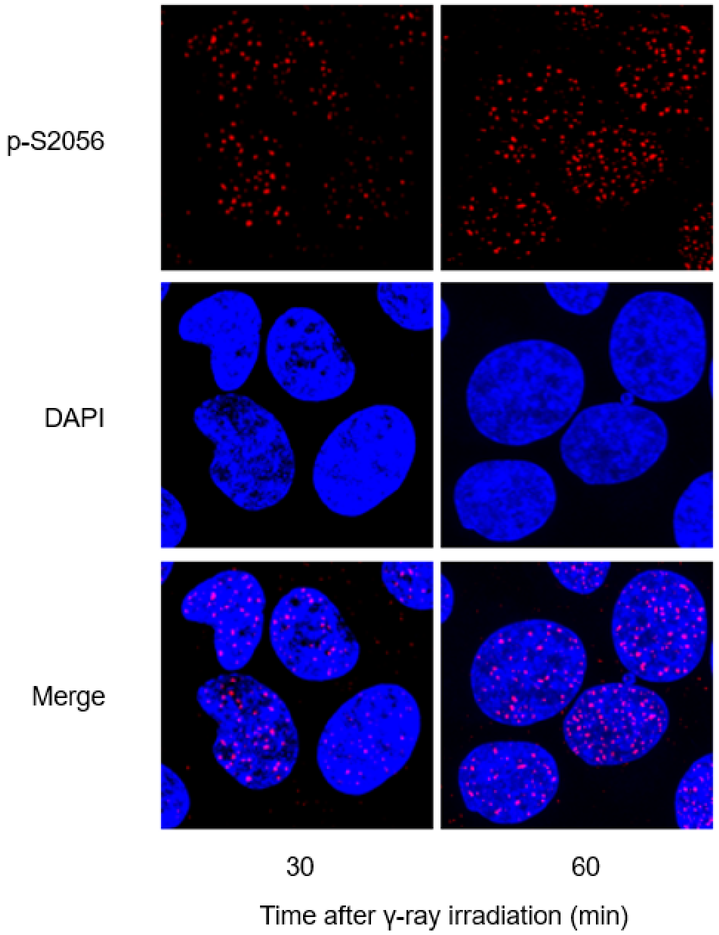

Supplementary Figure S20. Temporal changes in the phosphorylation of DNA-PKcs at S2056 following γ-ray irradiation. H1299 cells on microscope cover glasses were irradiated with γ-ray (5 Gy), and harvested after 30 min and 60 min for staining with an antibody against p-S2056 and with DAPI. The stained cells were analyzed by confocal microscope.

Supplementary Figure S21

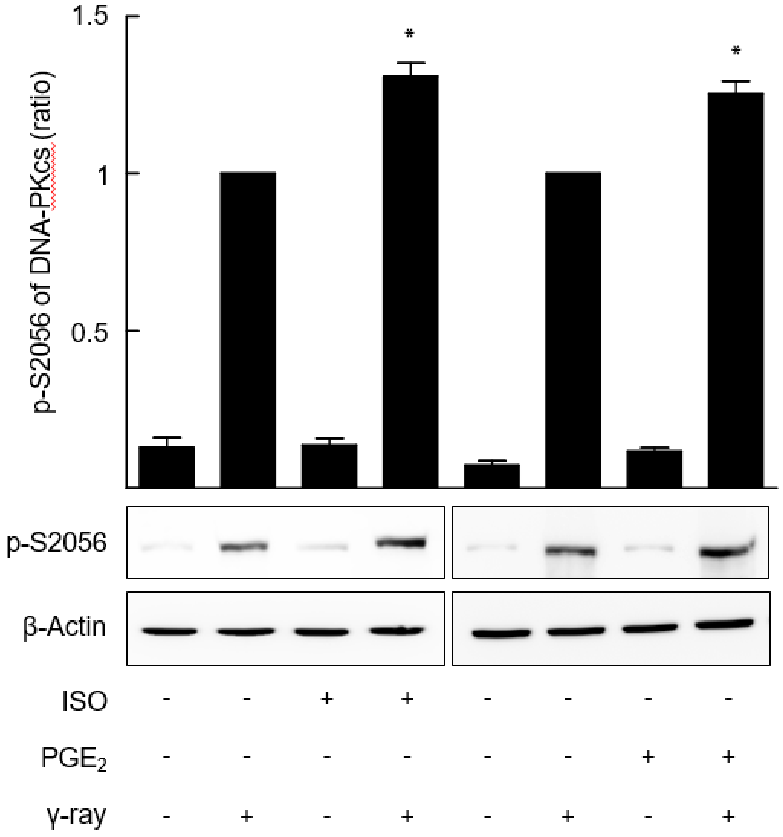

Supplementary Figure S21. Effects of the isoproterenol or PGE2 on S2056 phosphorylation following γ-ray irradiation in A549 cells. A549 cells pretreated with 1 μM isoproterenol (ISO) or 20 μM PGE2 for 30 min were irradiated with γ-rays (5 Gy) and harvested after 1 h for western blot analysis.

Supplementary Figure S22

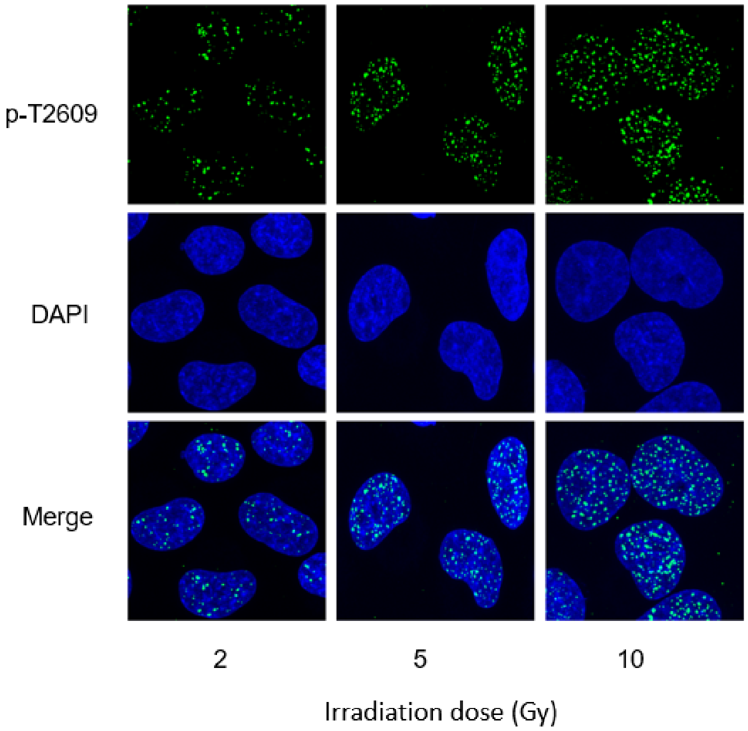

Supplementary Figure S22. Effects of the irradiation dose of γ-ray on the phosphorylation of DNA-PKcs at T2609. H1299 cells on microscope cover glasses were irradiated with different doses of γ-ray (2, 5, 10 Gy), and harvested after 1 h for staining with an antibody against p-T2609 and with DAPI. The stained cells were analyzed by confocal microscope.

Supplementary Figure S23

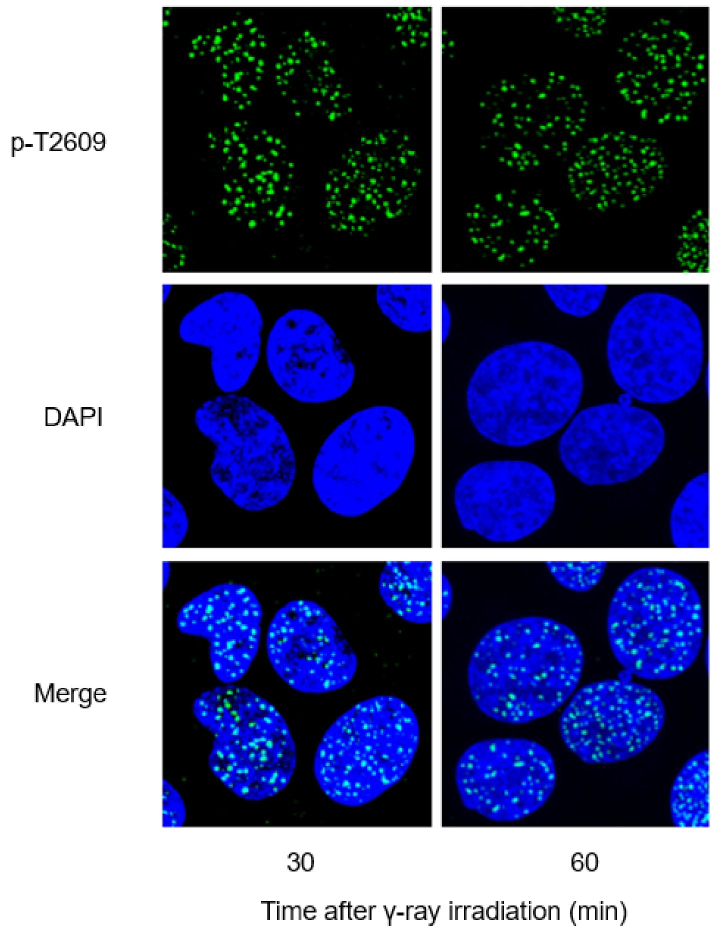

Supplementary Figure S23. Temporal changes in the phosphorylation of DNA-PKcs at T2609 following γ-ray irradiation. H1299 cells on microscope cover glasses were irradiated with γ-ray (5 Gy), and harvested after 30 min and 60 min for staining with an antibody against p-T2609 and with DAPI. The stained cells were analyzed by confocal microscope.

Supplementary Figure S24

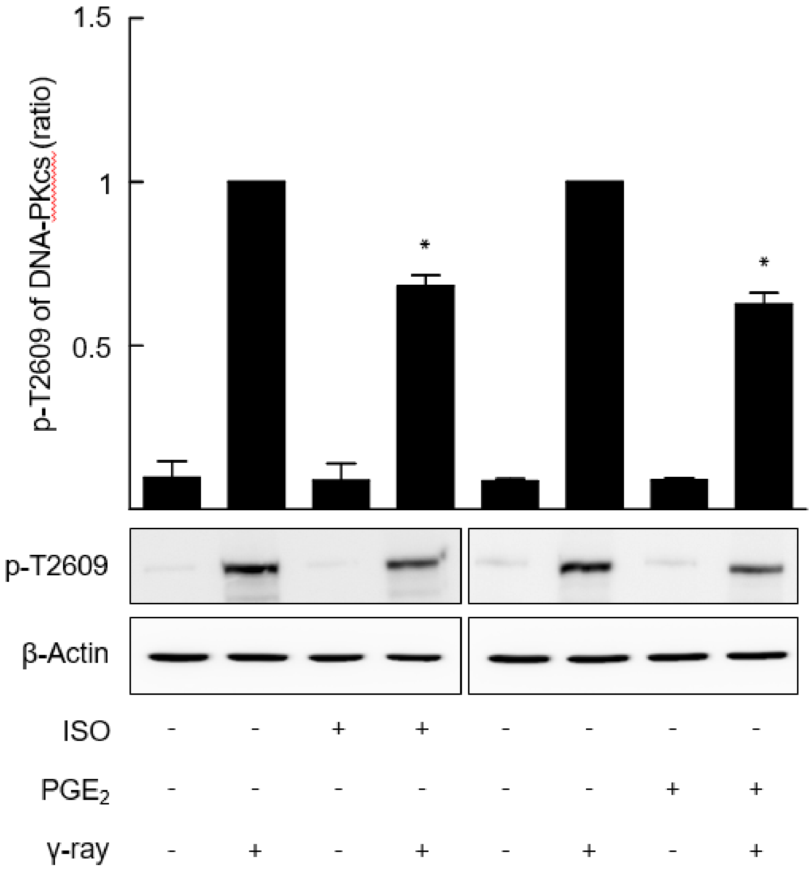

Supplementary Figure S24. Effects of the isoproterenol or PGE on the T2609 phosphorylation following γ-ray irradiation in A549 cells. A549 cells pretreated with 1 μM isoproterenol or 20 μM PGE2 for 30 min were irradiated with γ-rays (5 Gy) and harvested after 30min for western blot analysis.

Supplementary Figure S25

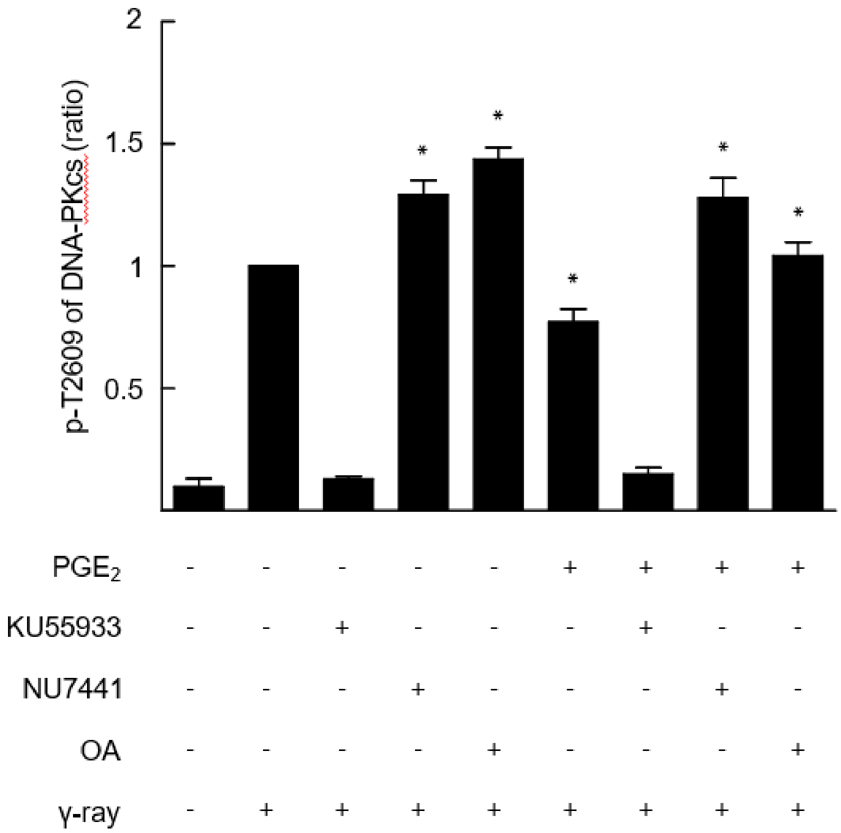

Supplementary Figure S25. Effects of KU55933 and NU7441, okadaic acid on the phosphorylation at T2609 following γ-irradiation in A549 cells. A549 cells were pretreated with 10 μM KU55933, 5 μM NU7441, or 100 nM okadaic acid (OA) for 30 min and then treated with 20 μM PGE2 for 30 min. The treated cells were irradiated with γ-rays (5 Gy) and harvested after 30 min for western blot analysis.

Supplementary Figure S26. The original unprocessed blots presented in Figures and Supplementary Figures and the images of replicate blots. The edges of some blot images with low background density were outlined with solid black-lined rectangles.

Figure 2e

Blots presented in Fig.

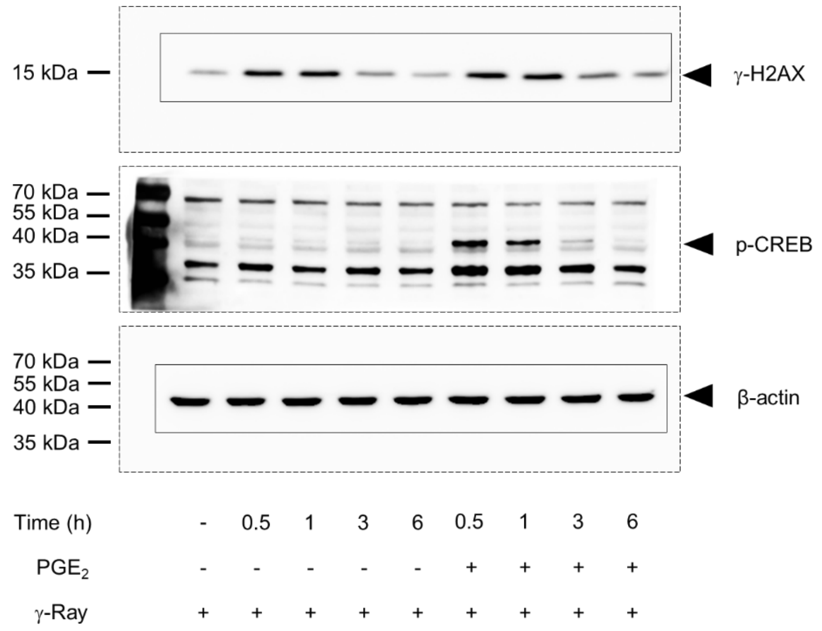

Figure 2e

Replicate blots

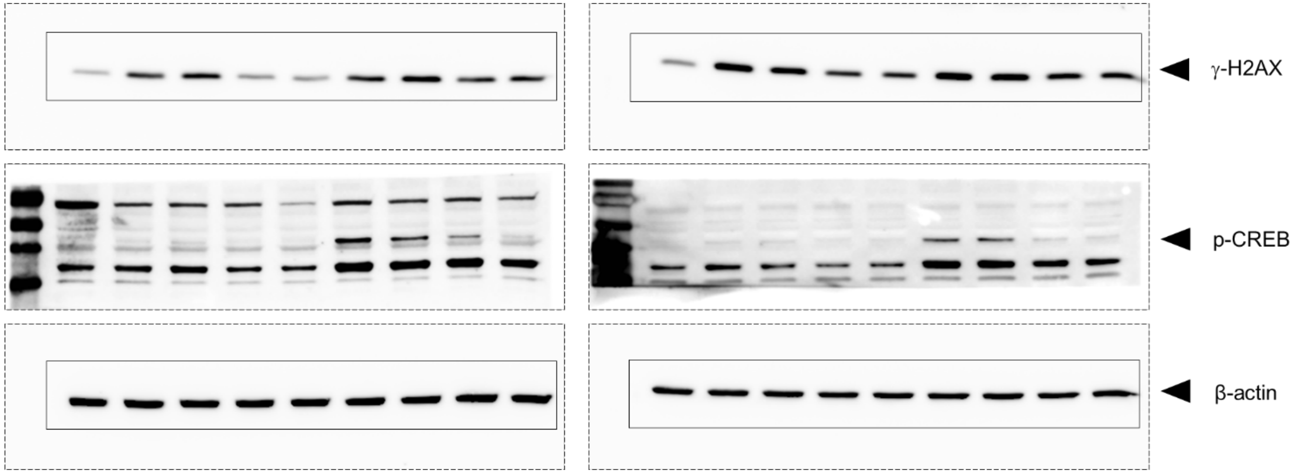

Figure 4c

Blots presented in Fig.

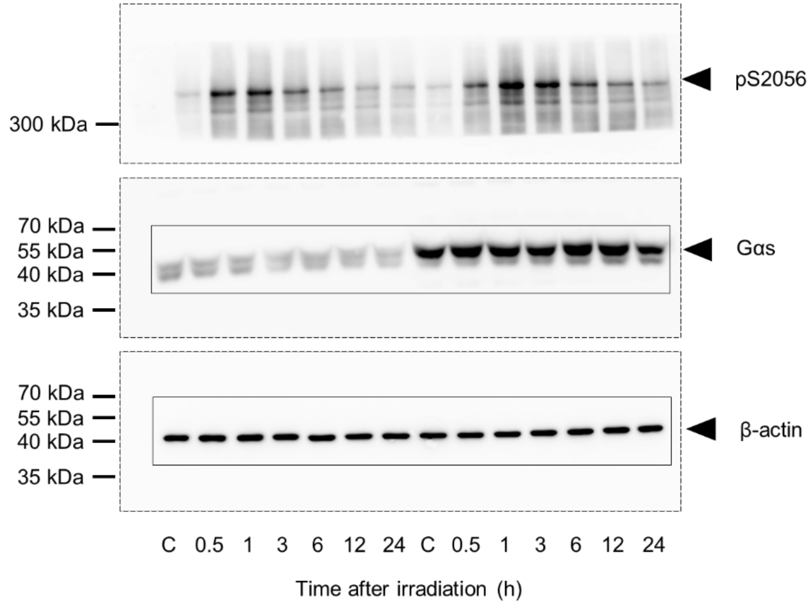

Figure 4c

Replicate blots

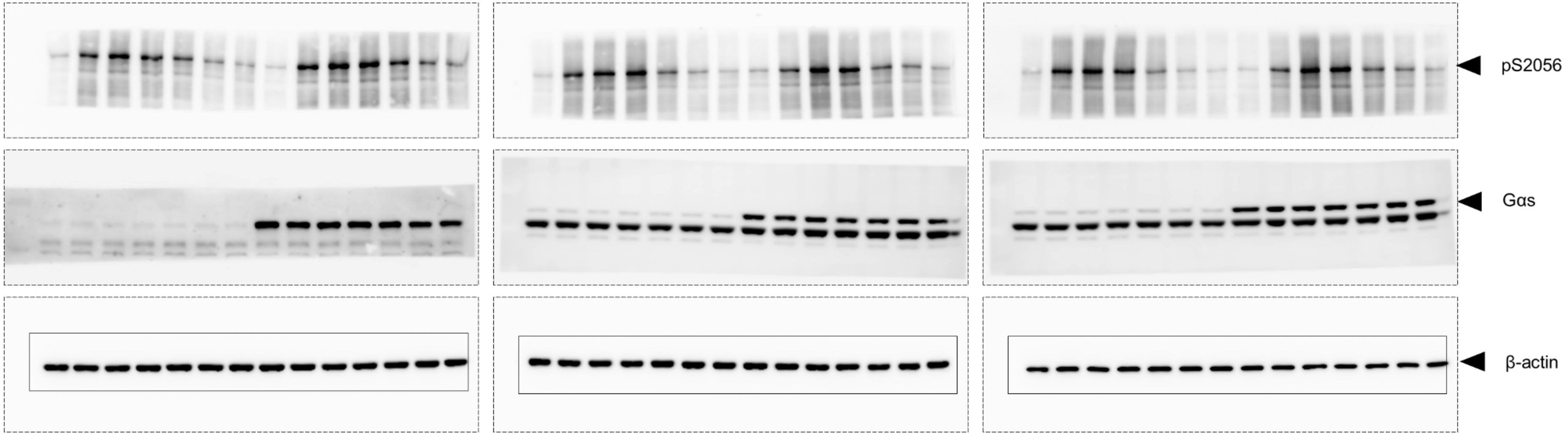

Figure 4d

Blots presented in Fig.

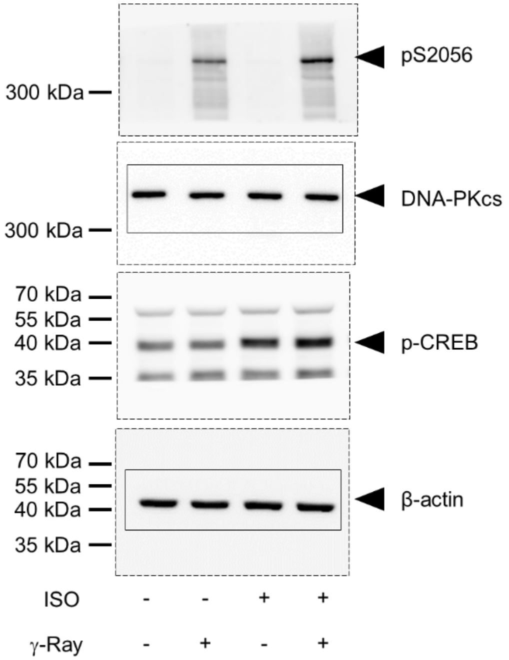

Figure 4d

Replicate blots

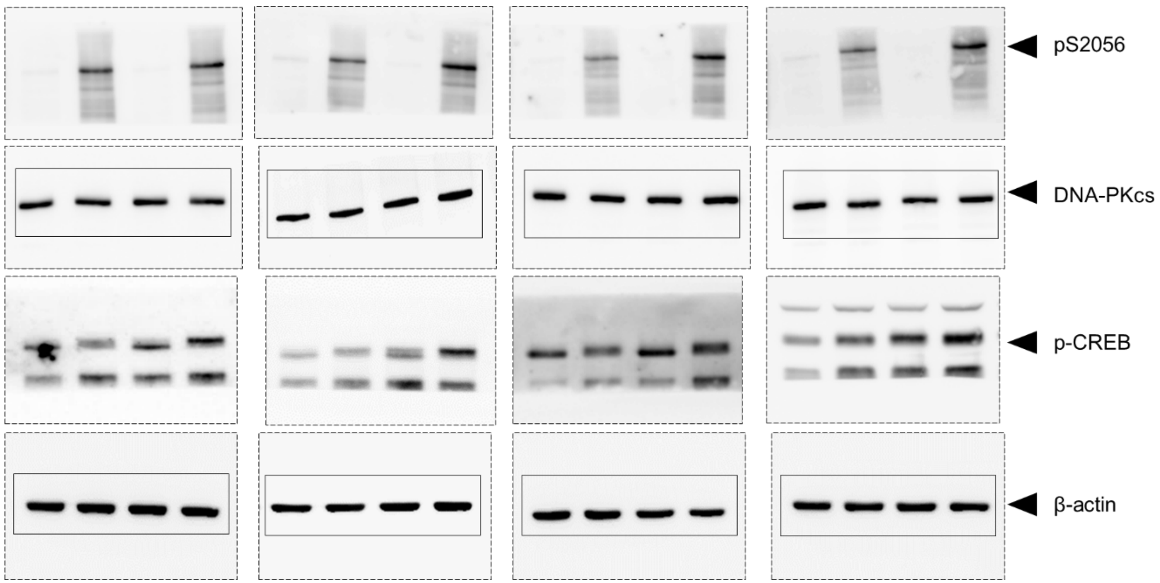

Figure 4e

Blots presented in Fig.

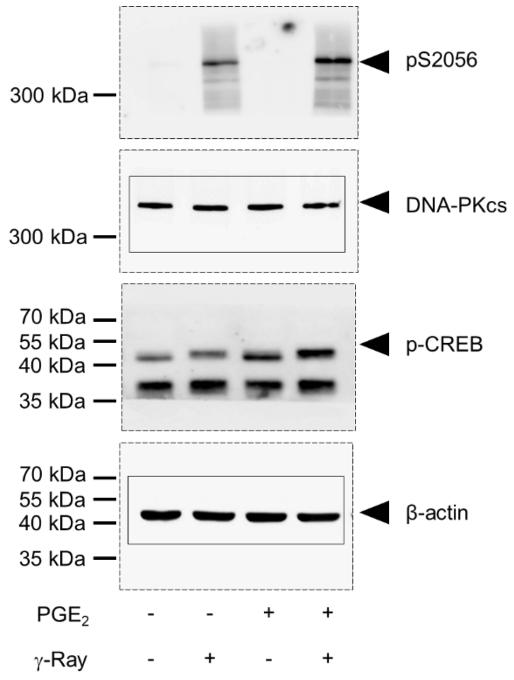

Figure 4e

Replicate blots

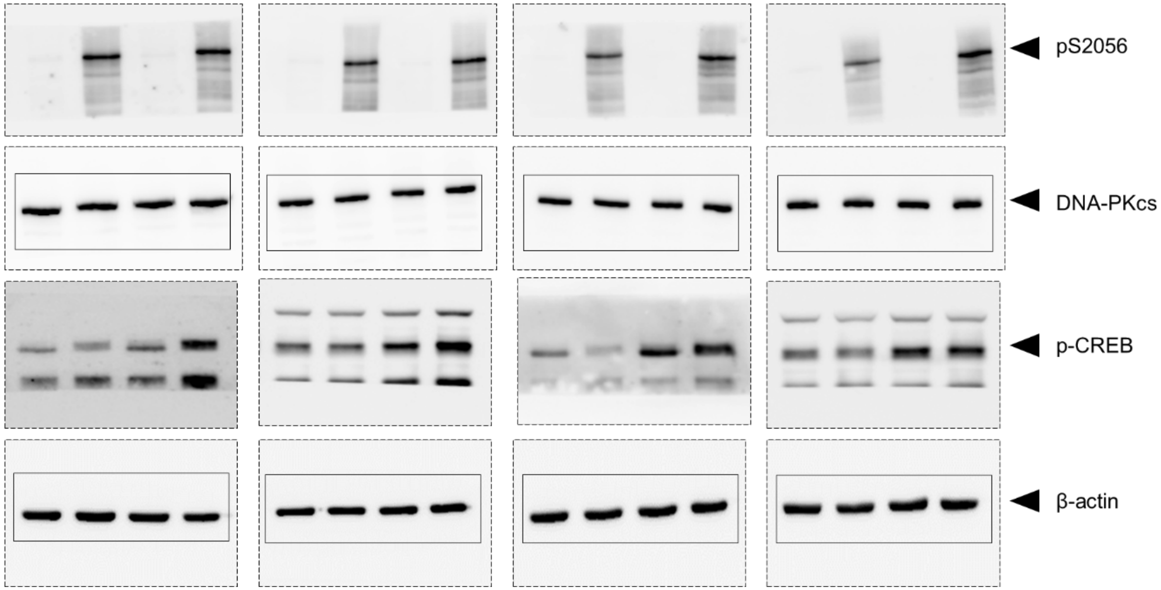

Figure 5c

Blots presented in Fig.

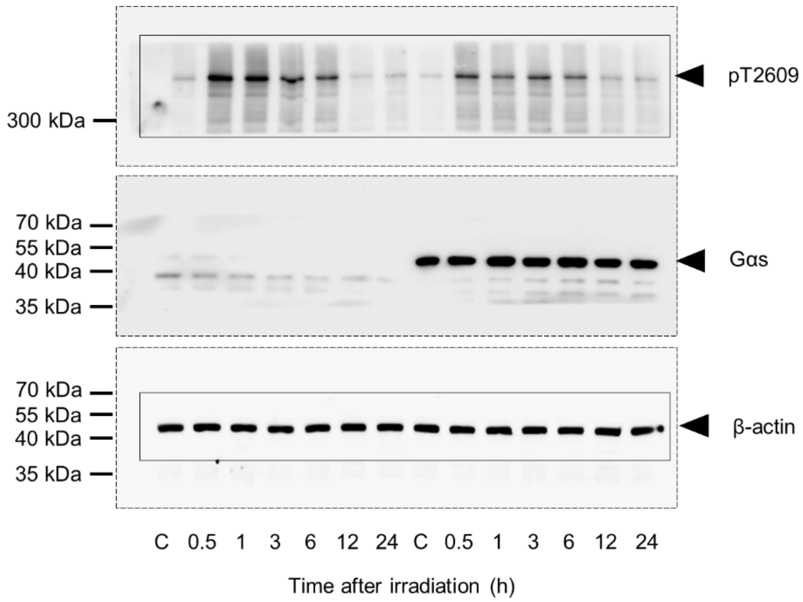

Figure 5c

Replicate blots

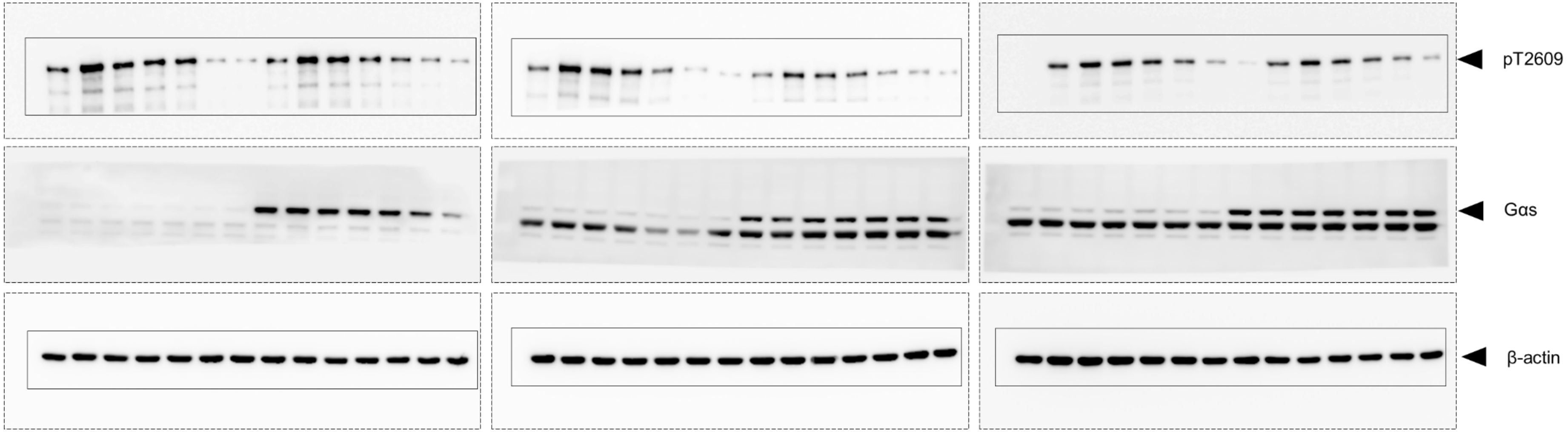

Figure 5d

Blots presented in Fig.

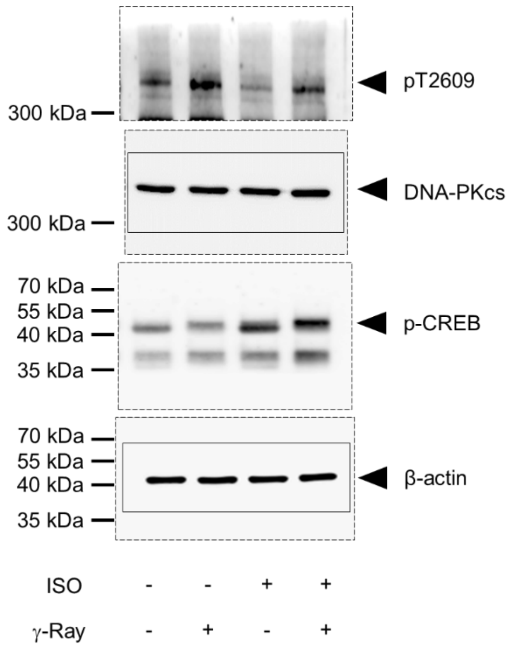

Figure 5d

Replicate blots

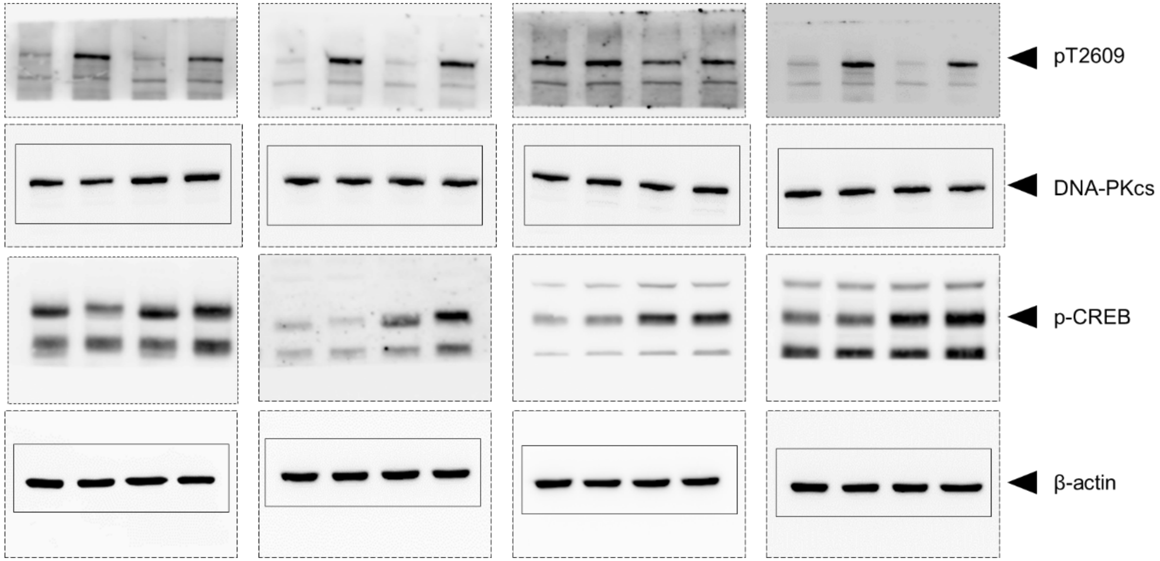

Figure 5e

Blots presented in Fig.

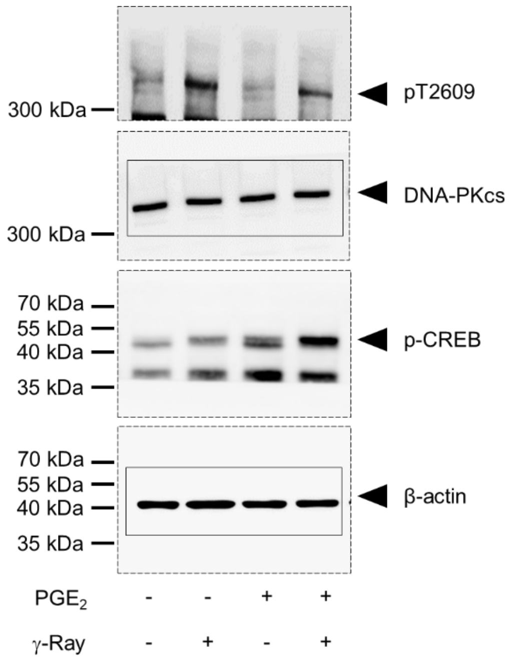

Figure 5e

Replicate blots

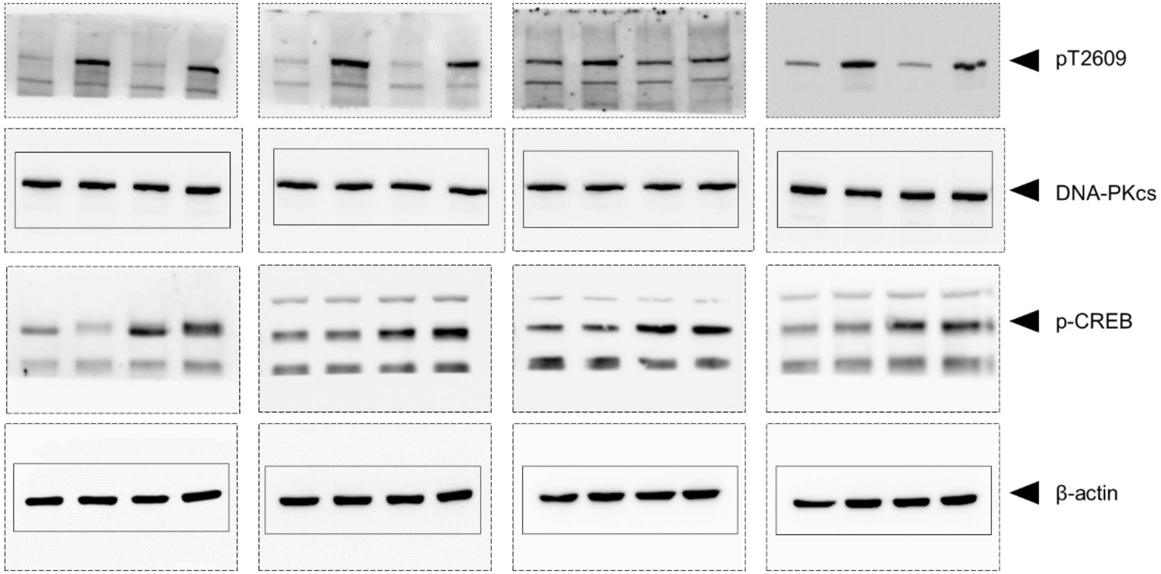

Figure 6a

Blots presented in Fig.

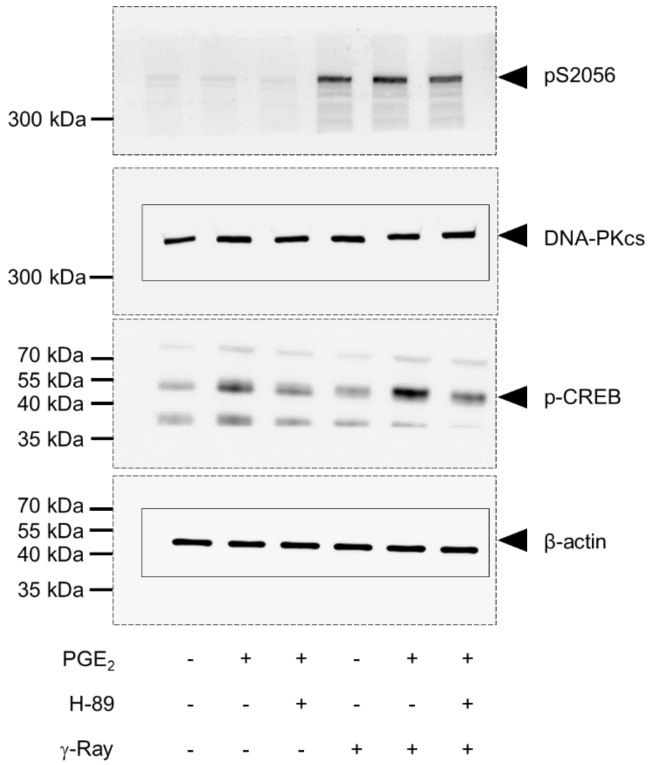

Figure 6a

Replicate blots

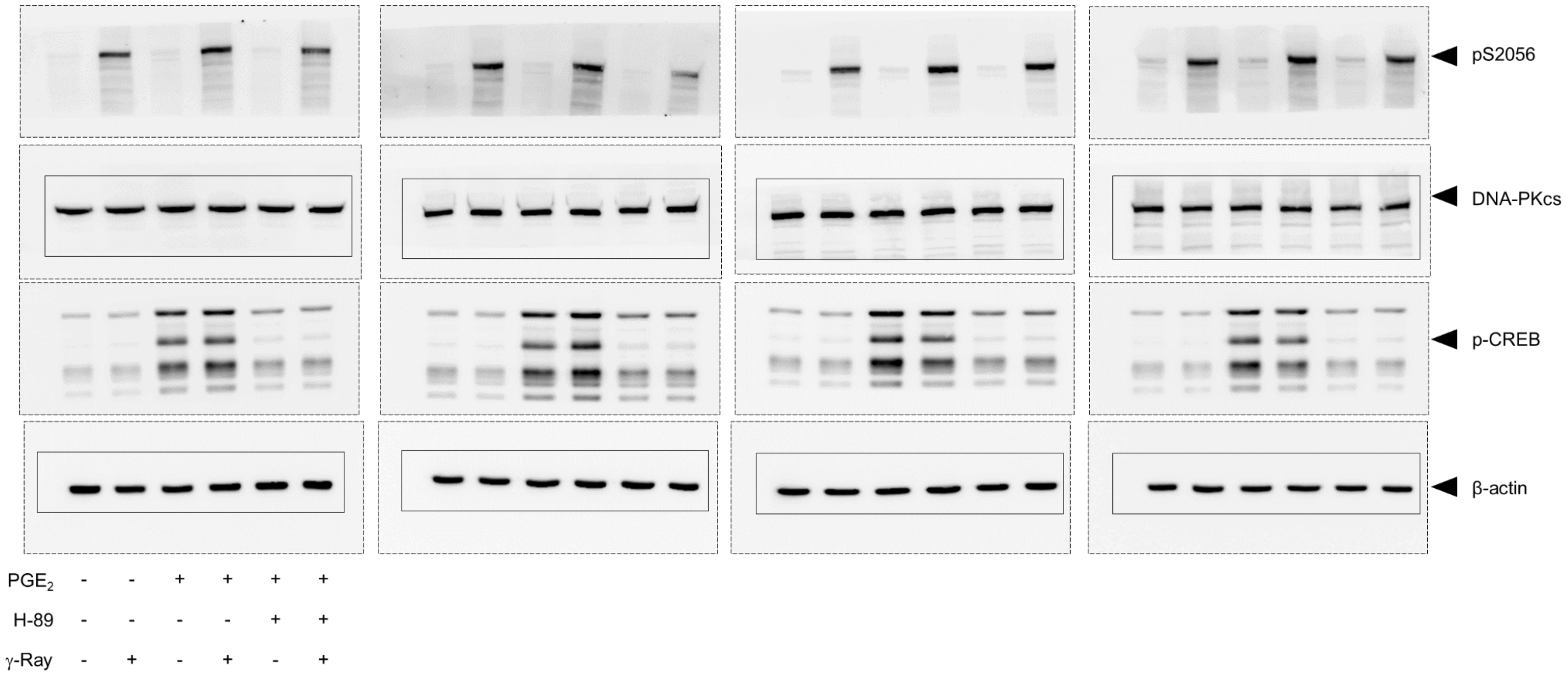

Figure 6b

Blots presented in Fig.

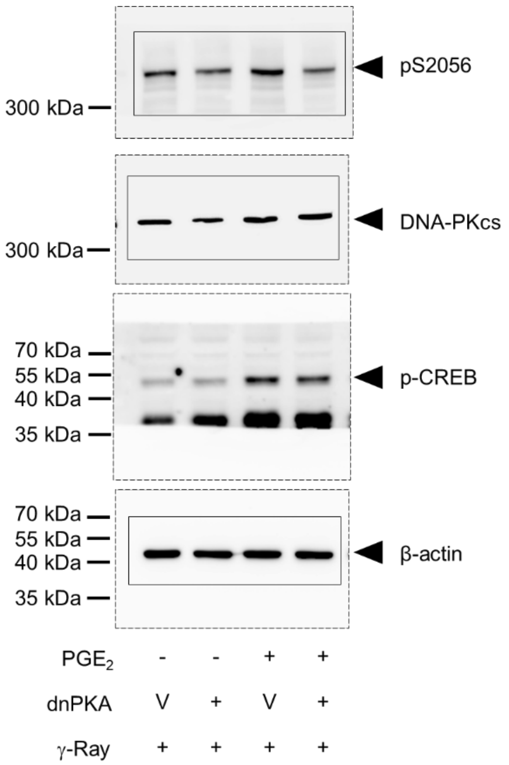

Figure 6b

Replicate blots

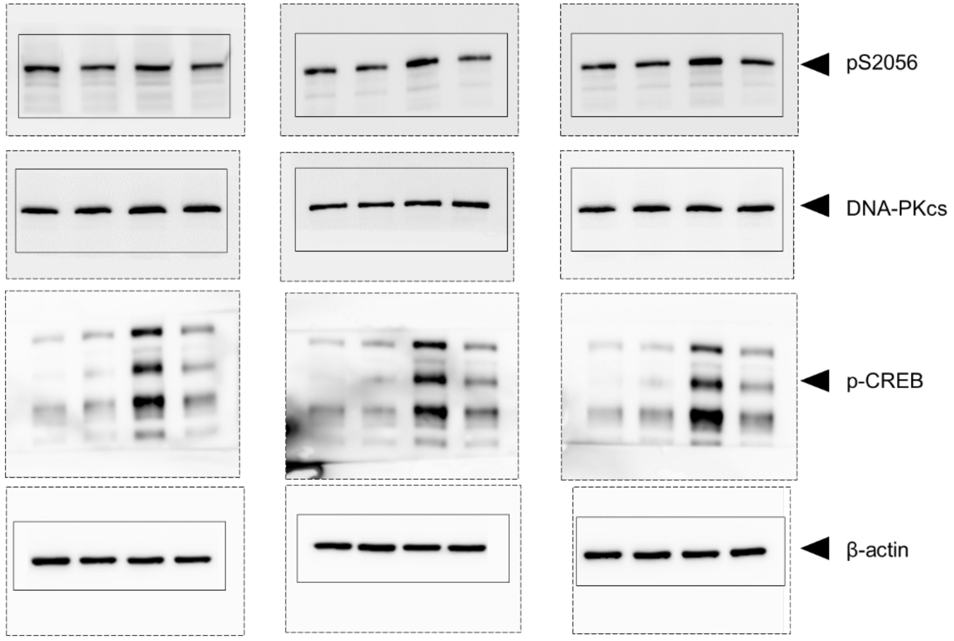

Figure 6c

Blots presented in Fig.

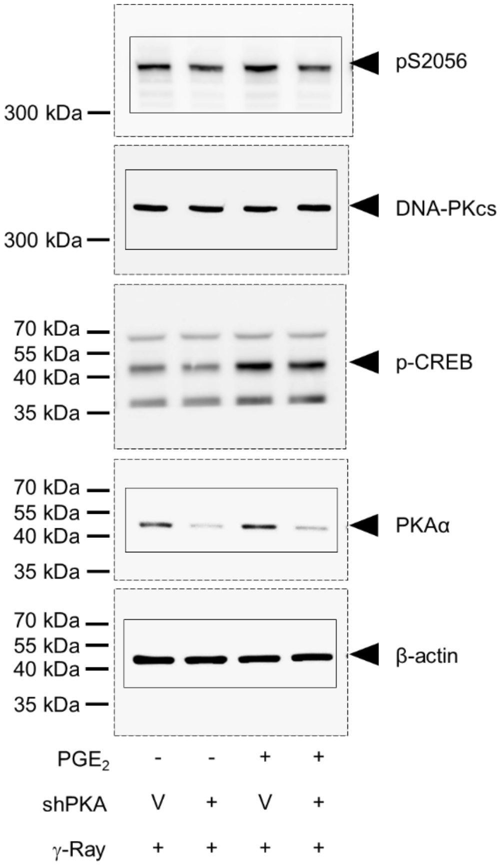

Figure 6c

Replicate blots

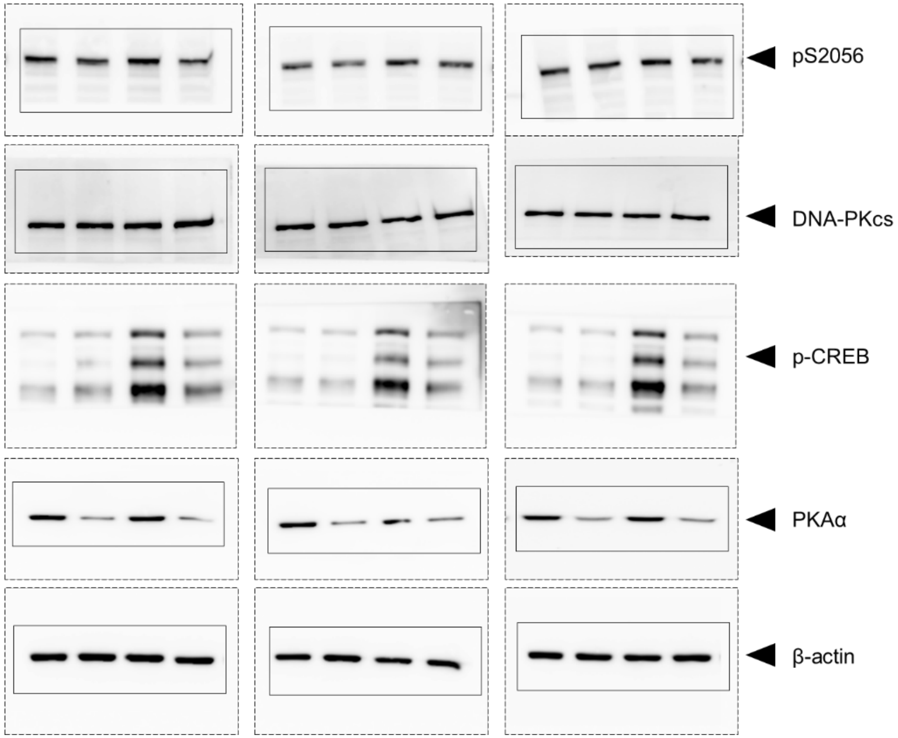

Figure 6d

Blots presented in Fig.

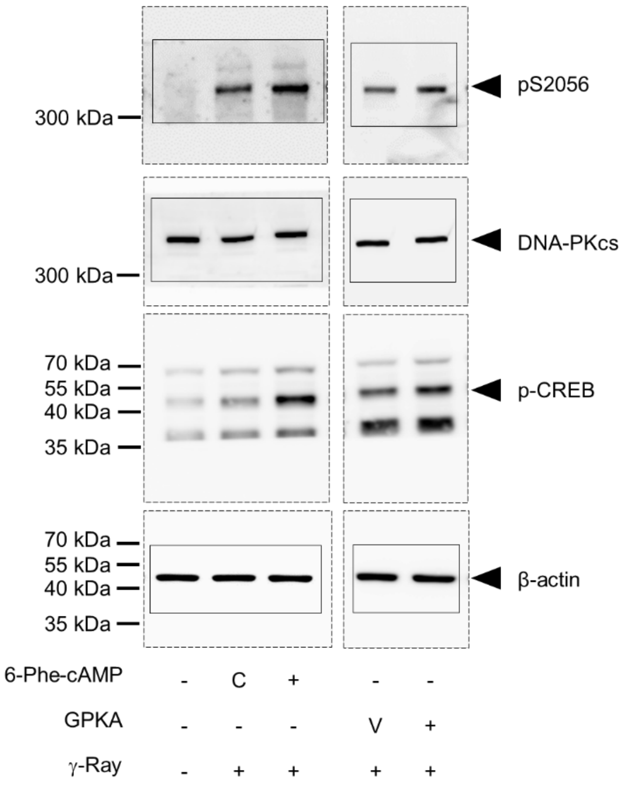

Figure 6d

Replicate blots

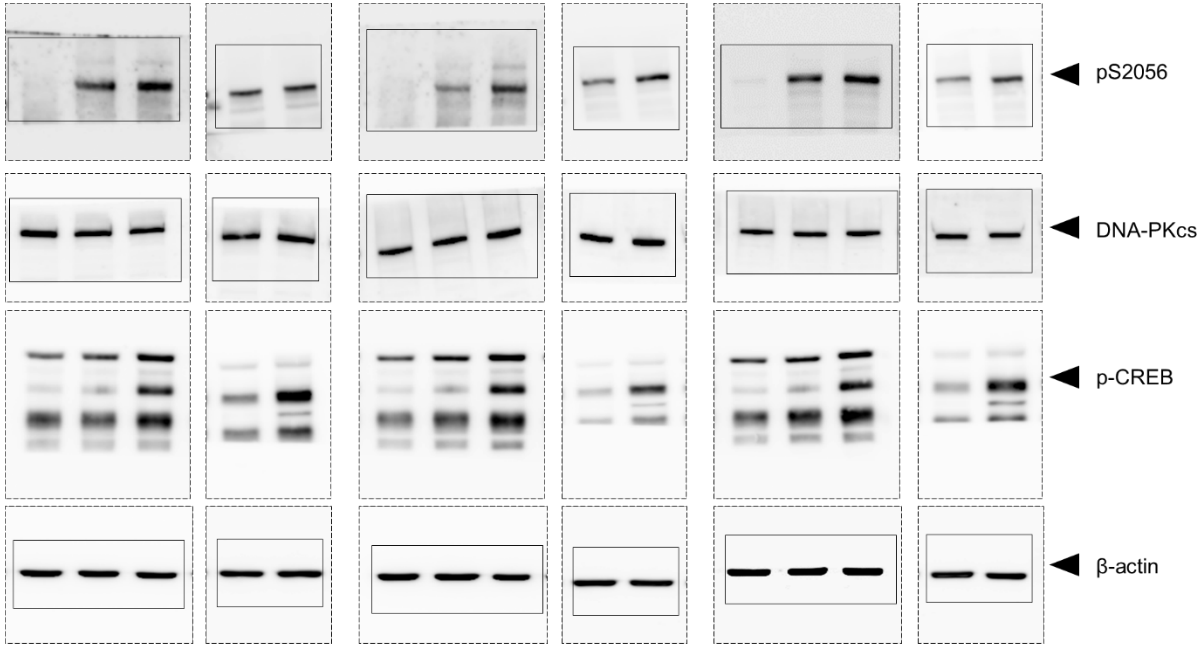

Figure 7a

Blots presented in Fig.

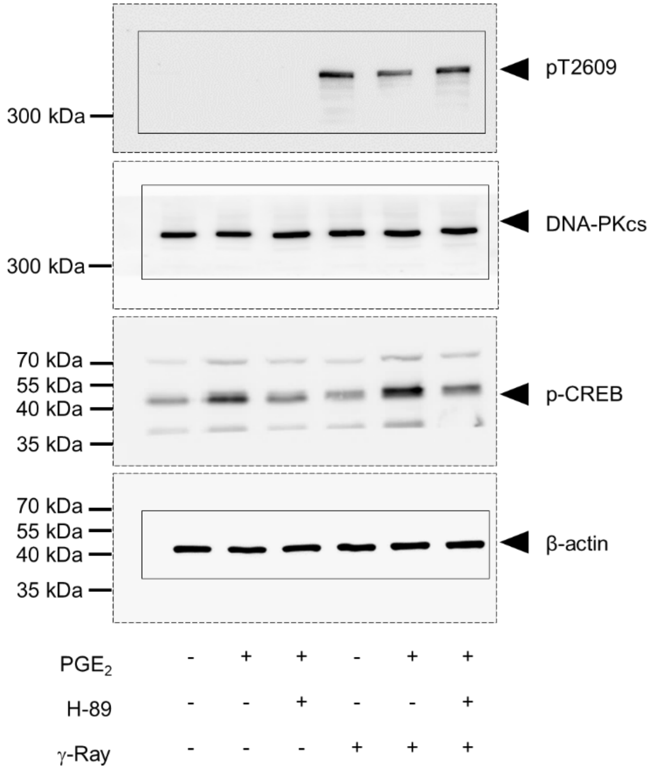

Figure 7a

Replicate blots

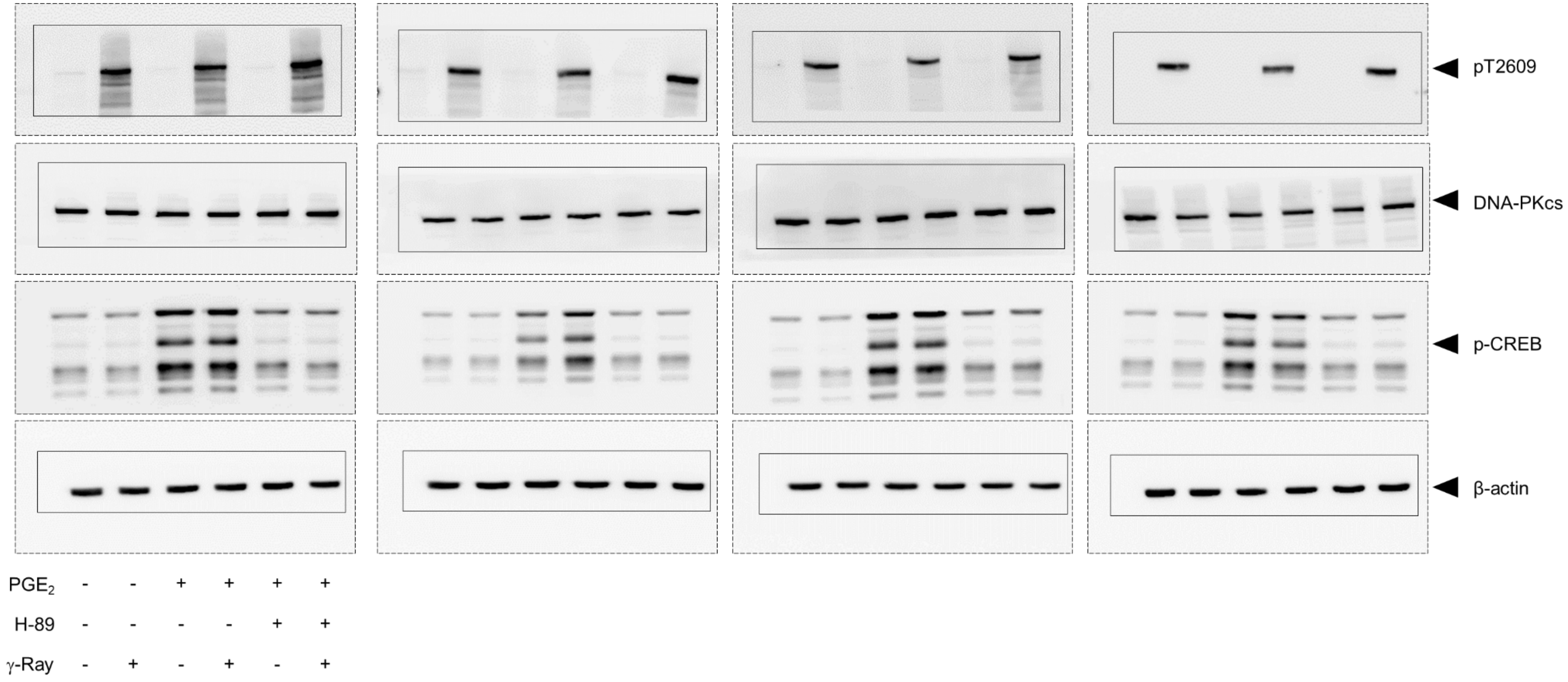

Figure 7b

Blots presented in Fig.

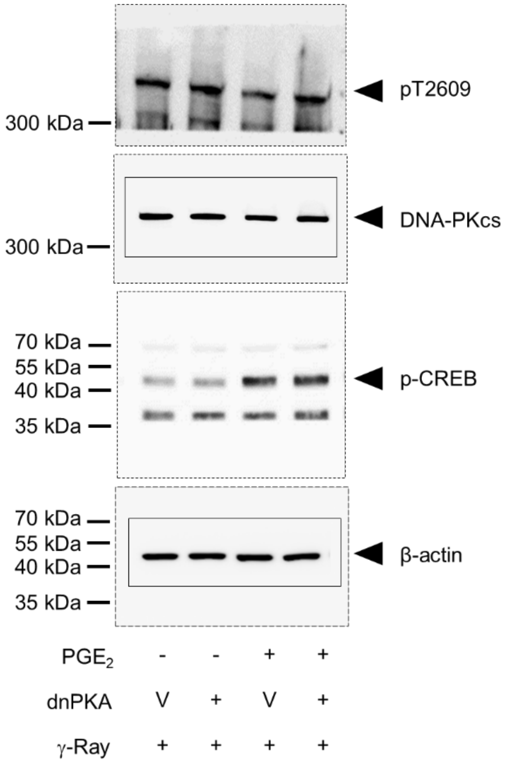

Figure 7b

Replicate blots

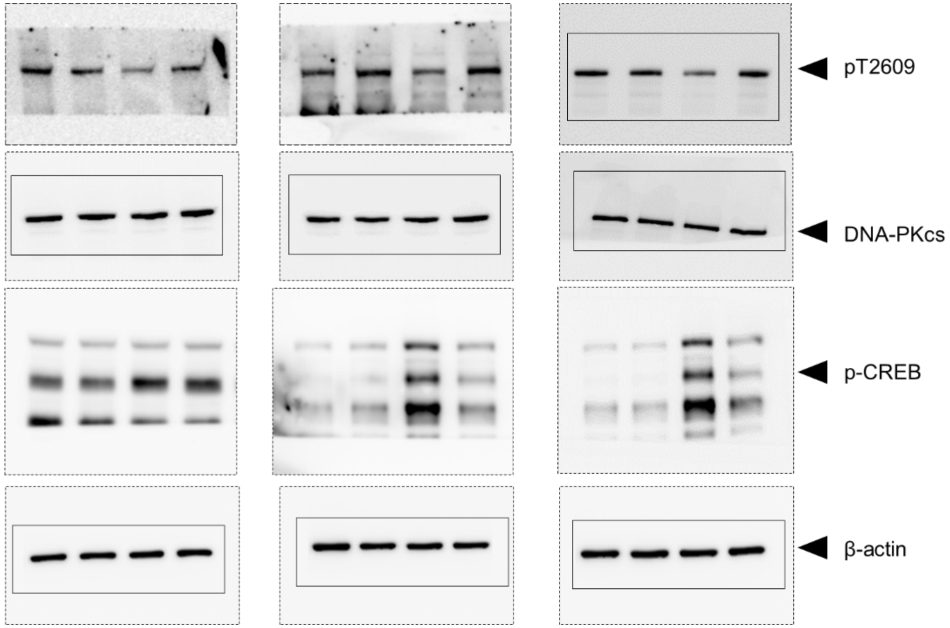

Figure 7c

Blots presented in Fig.

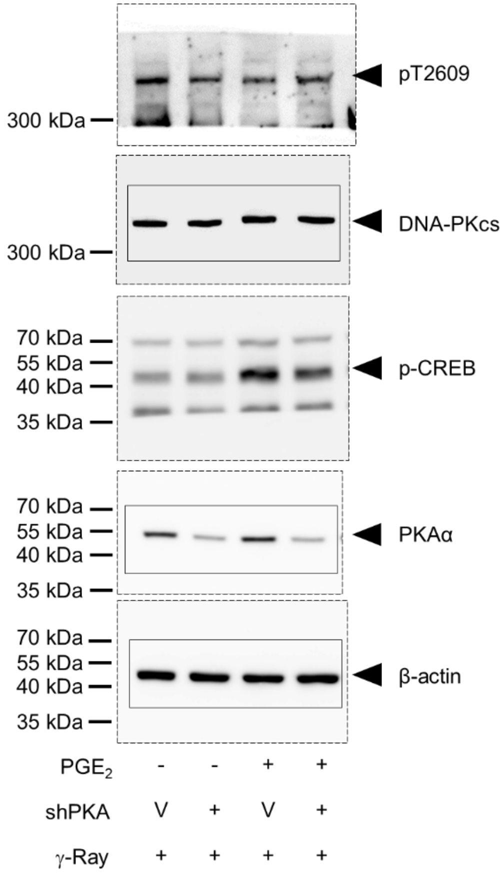

Figure 7c

Replicate blots

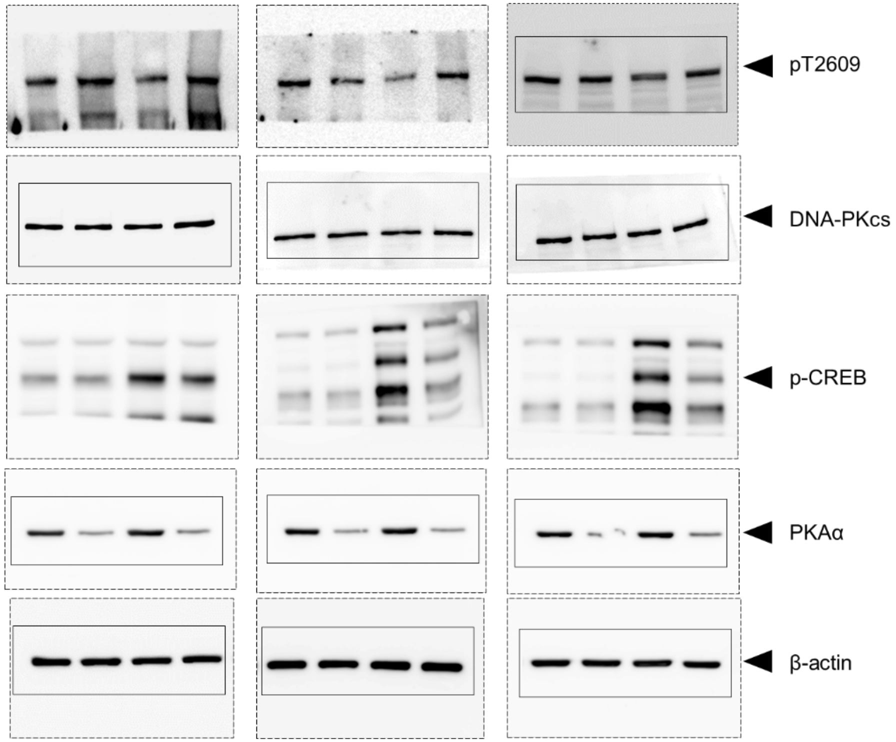

Figure 7d

Blots presented in Fig.

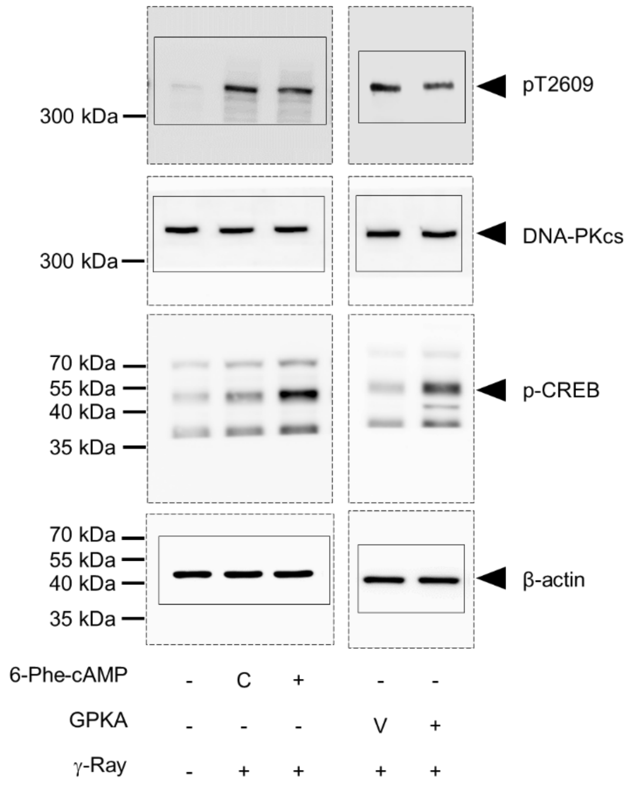

Figure 7d

Replicate blots

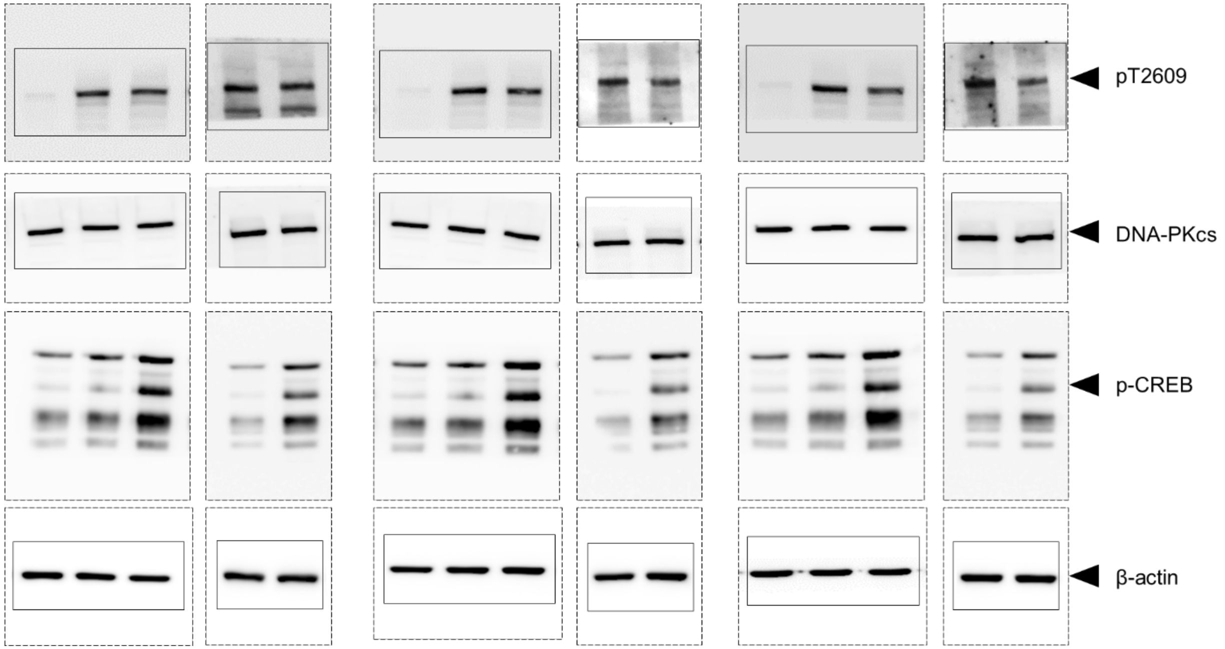

Figure 8a

Blots presented in Fig.

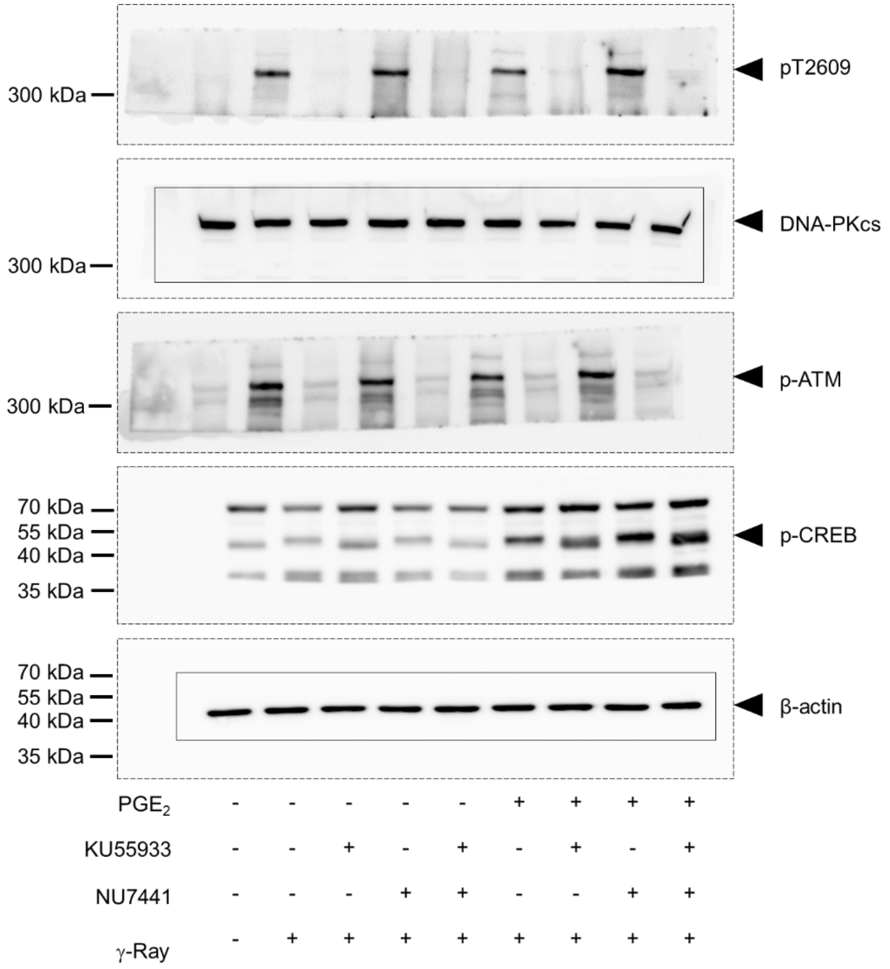

Figure 8a

Replicate blots

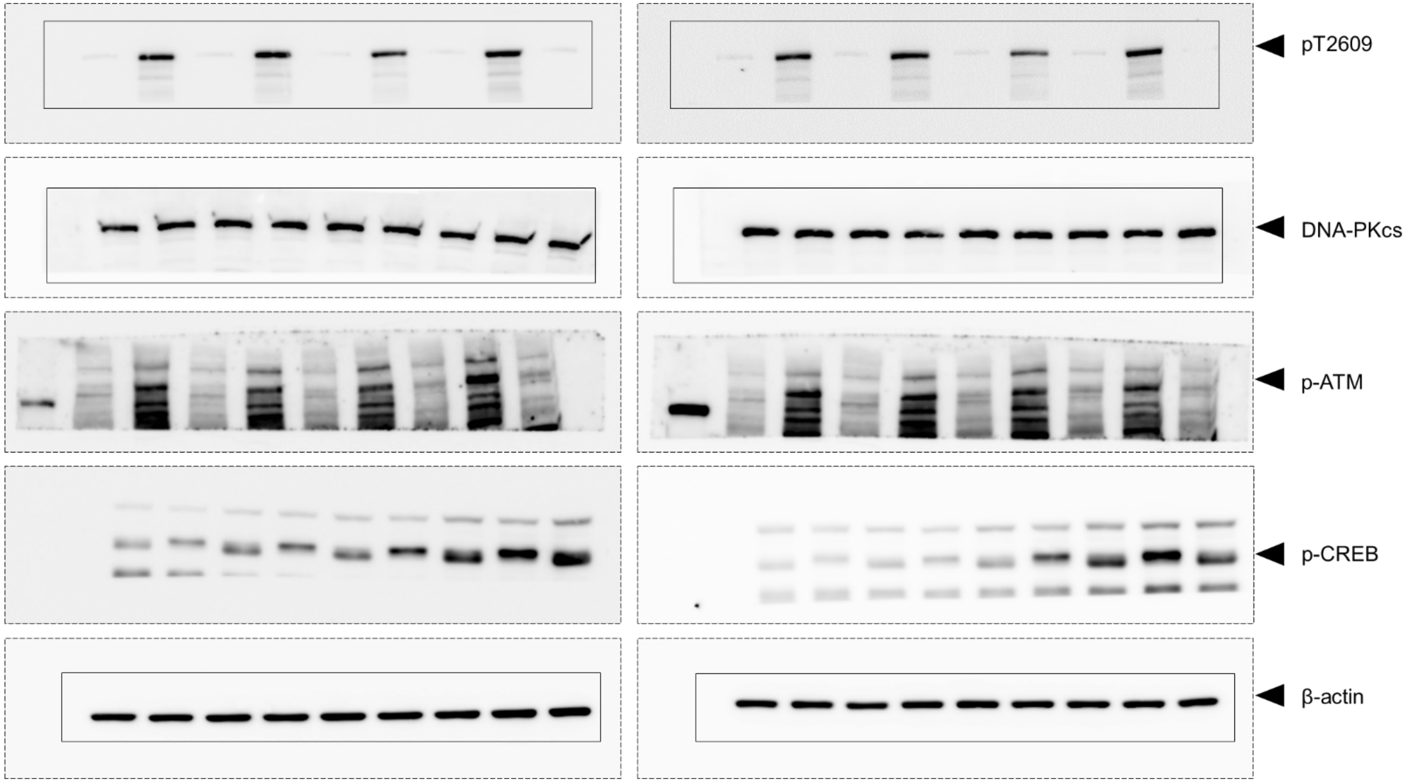

Figure 8b

Blots presented in Fig.

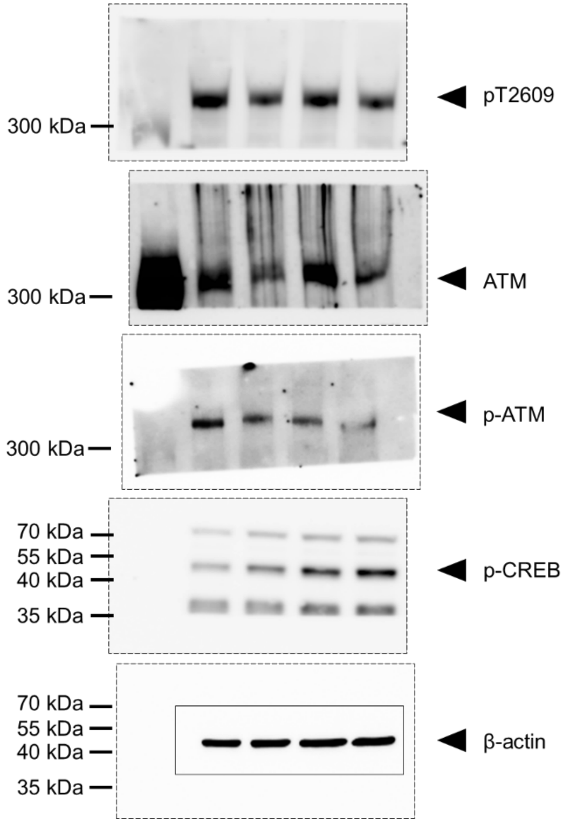

Figure 8b

Replicate blots

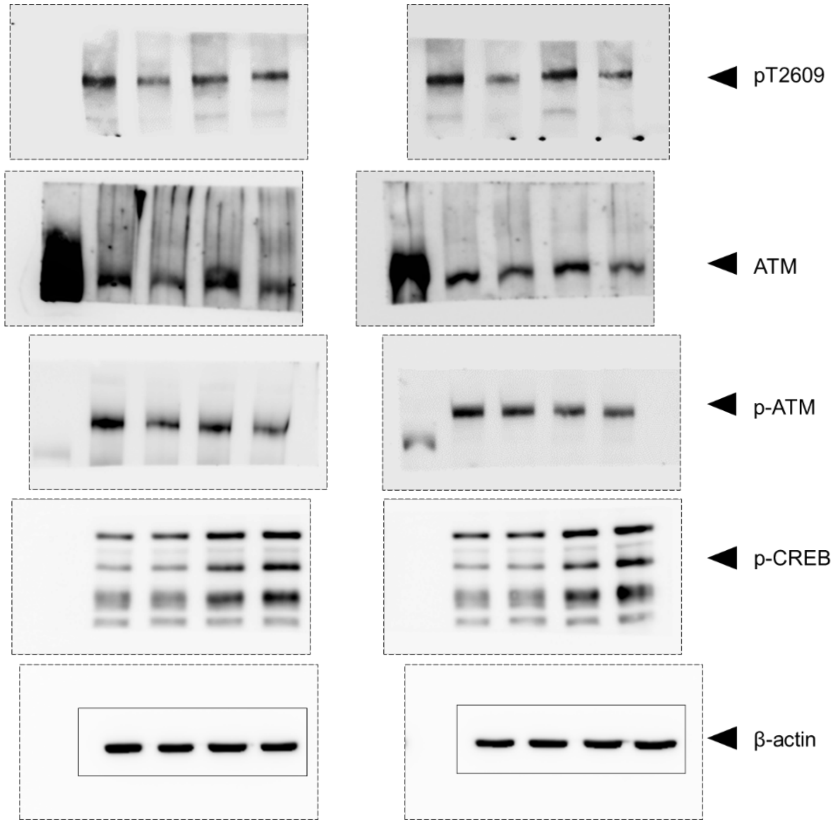

Figure 8c

Blots presented in Fig.

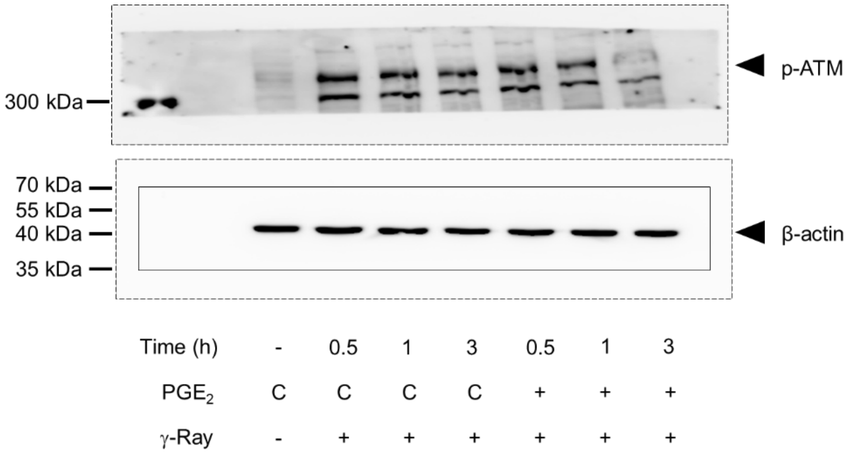

Figure 8c

Replicate blots

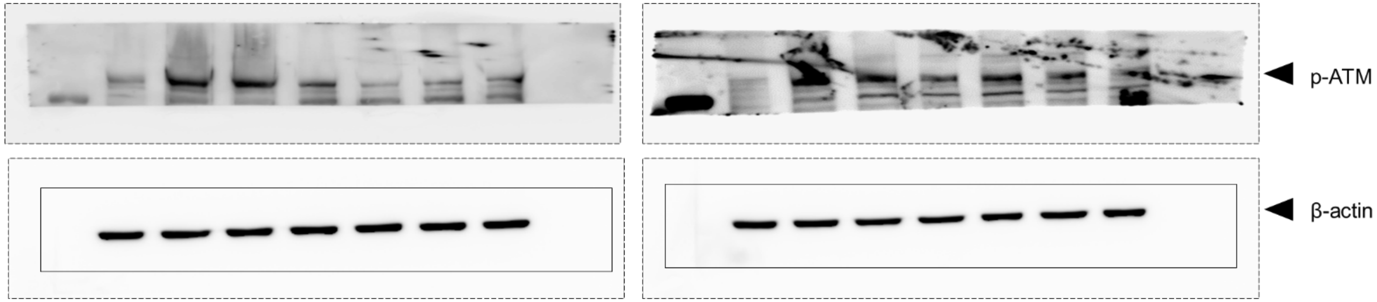

Figure 8d

Blots presented in Fig.

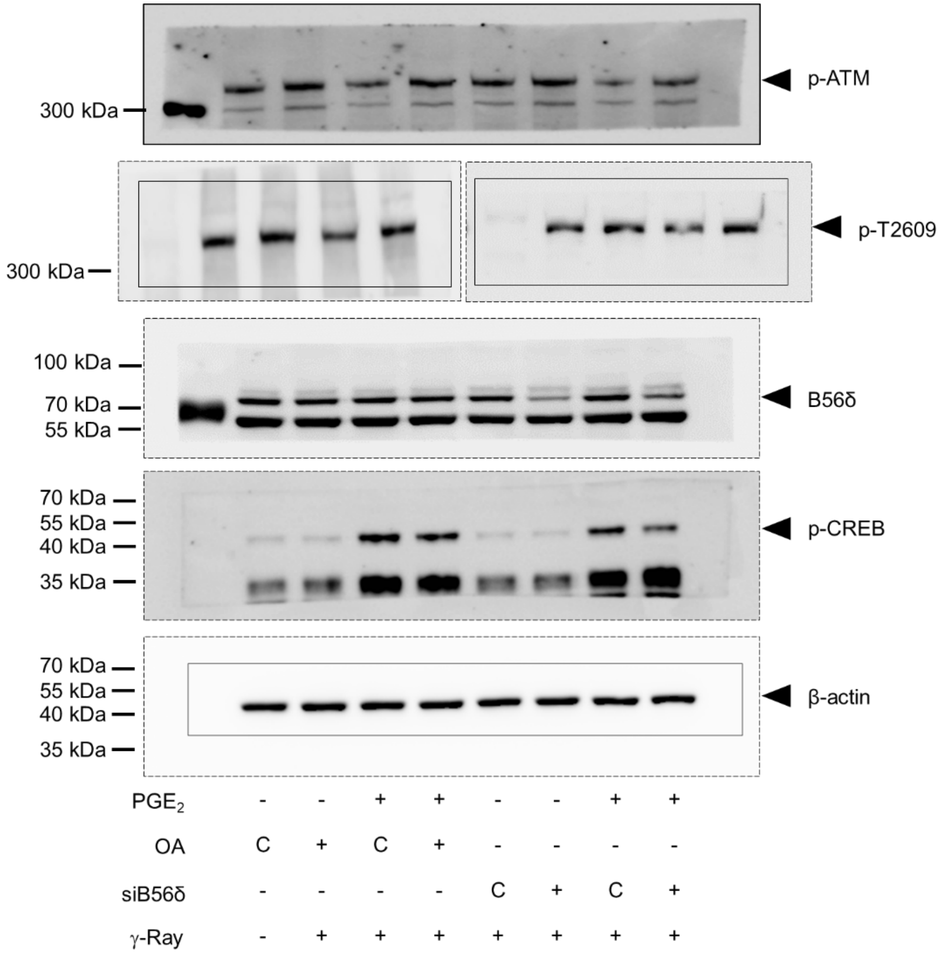

Figure 8d

Replicate blots

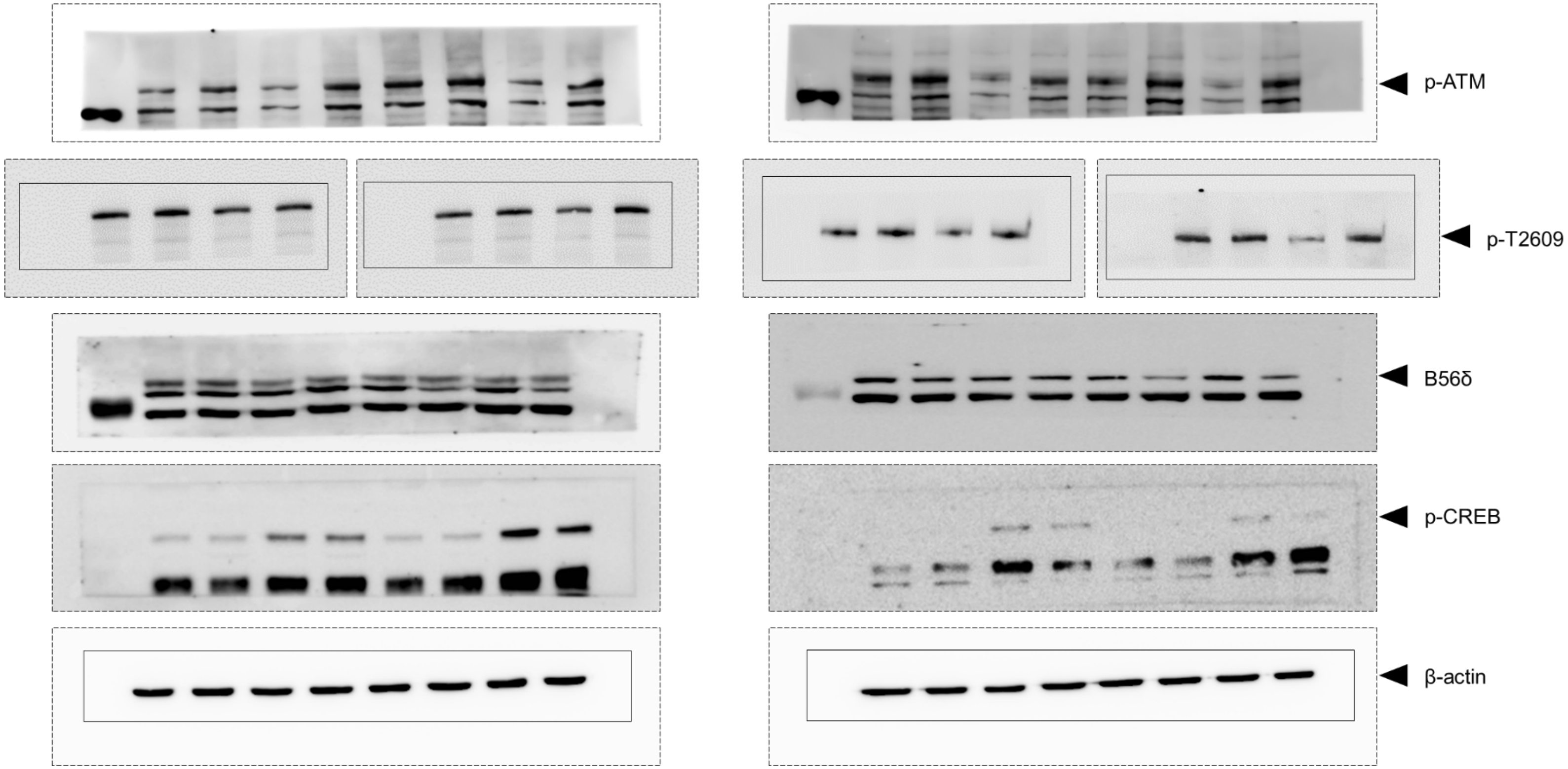

Figure 8e

Blots presented in Fig.

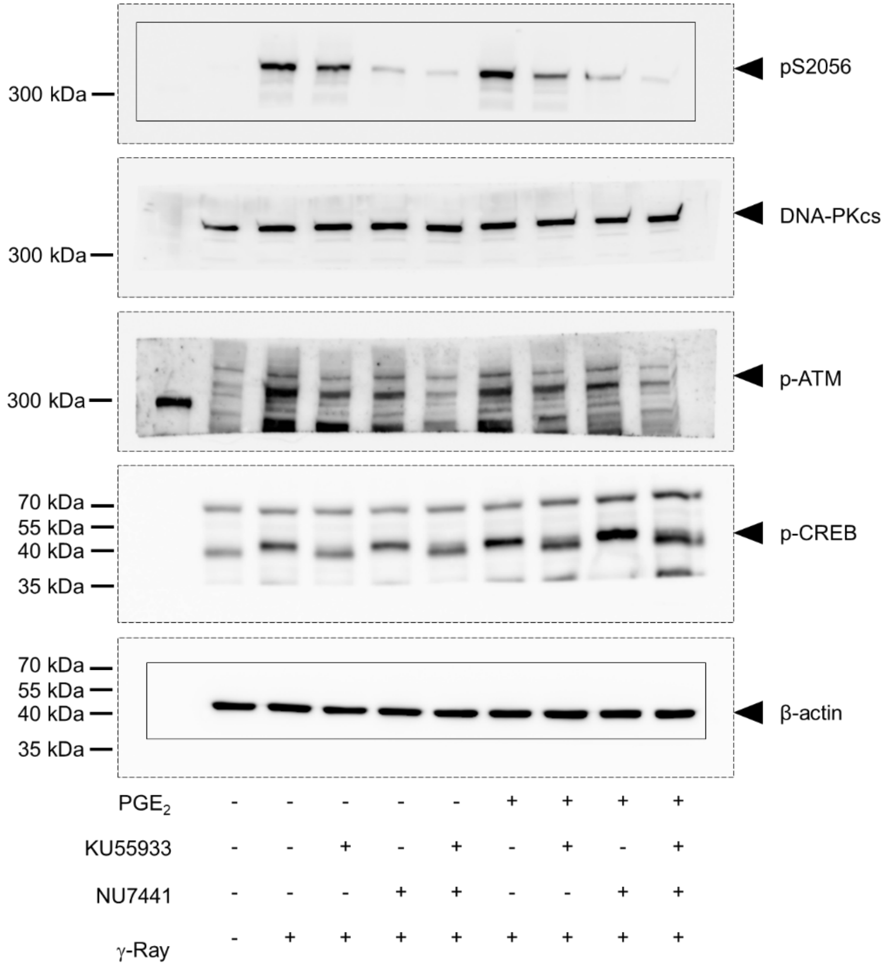

Figure 8e

Replicate blots

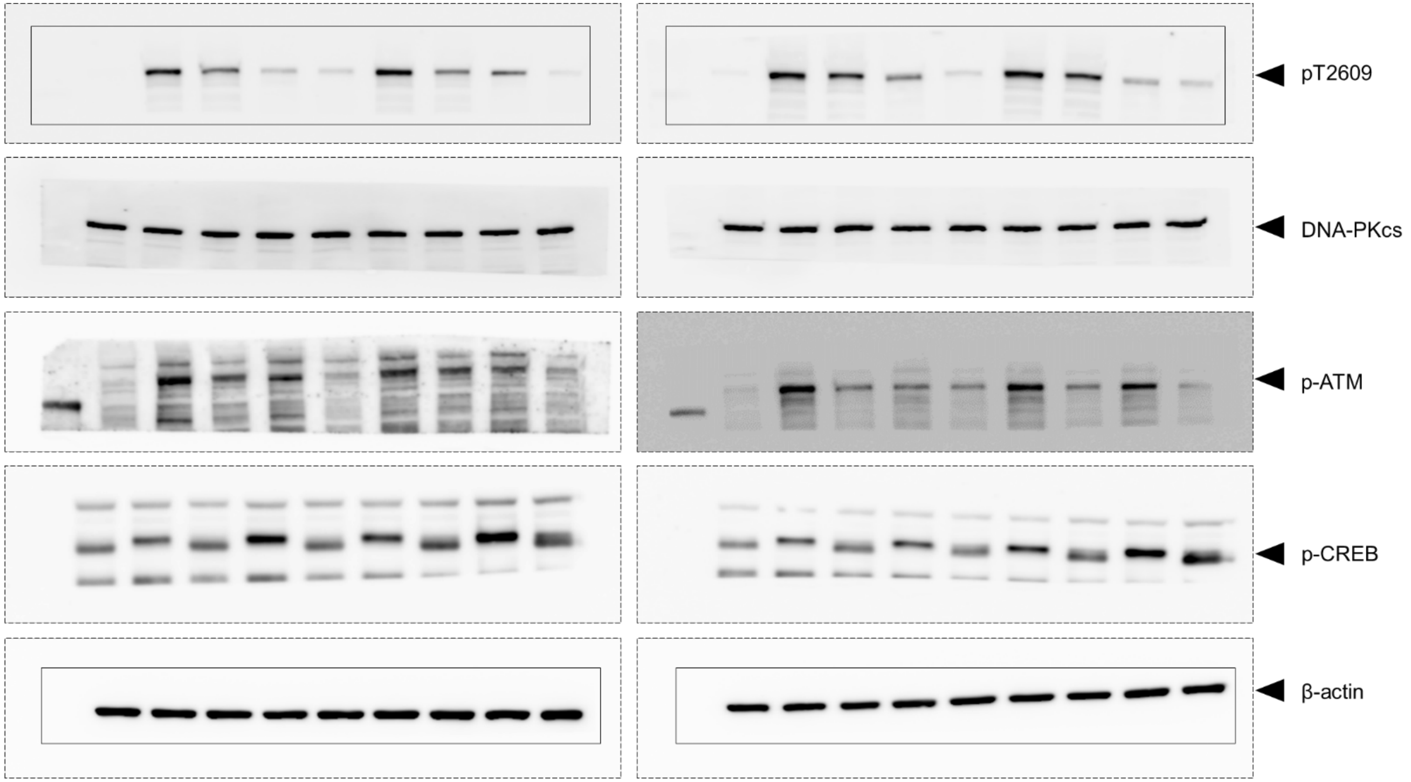

Supplementary Figure S6

Blots presented in Fig.

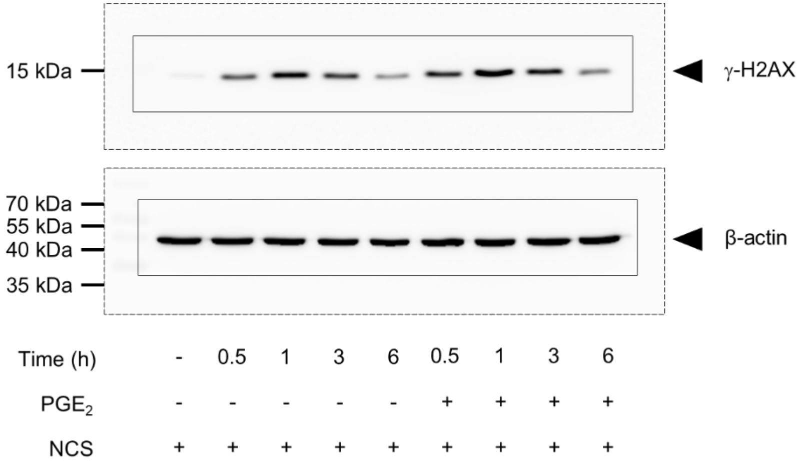

Supplementary Figure S6

Replicate blots

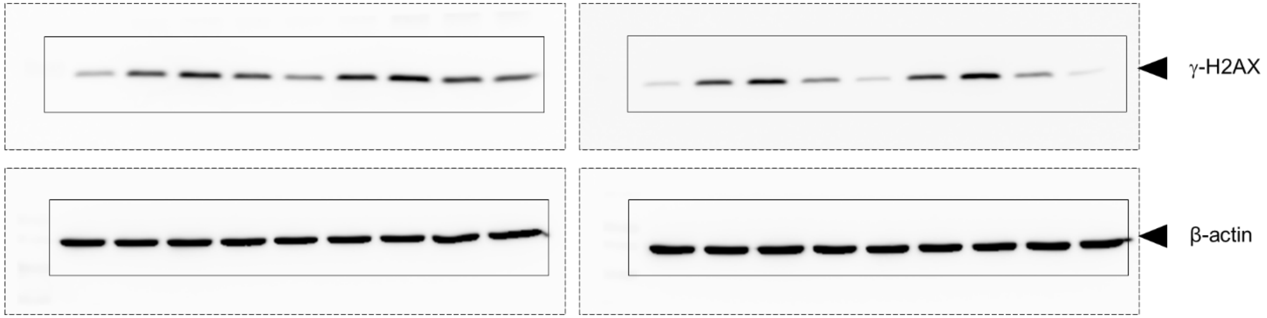

Supplementary Figure S7

Blots presented in Fig.

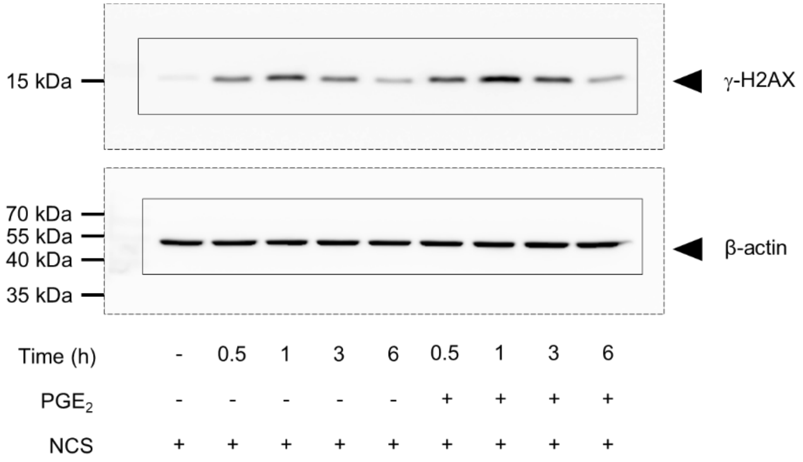

Supplementary Figure S7

Replicate blots

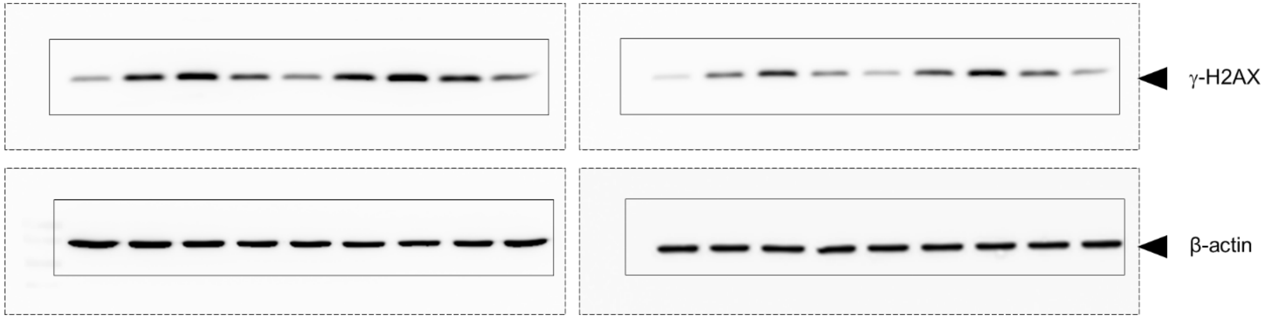

Supplementary Figure S13

Blots presented in Fig.

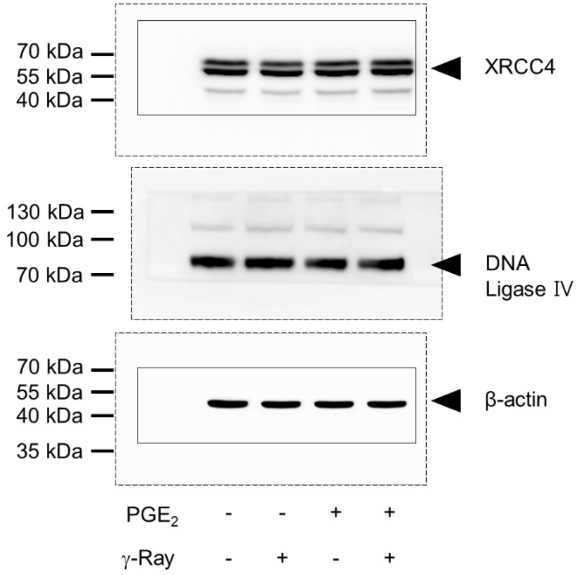

Supplementary Figure S13

Replicate blots

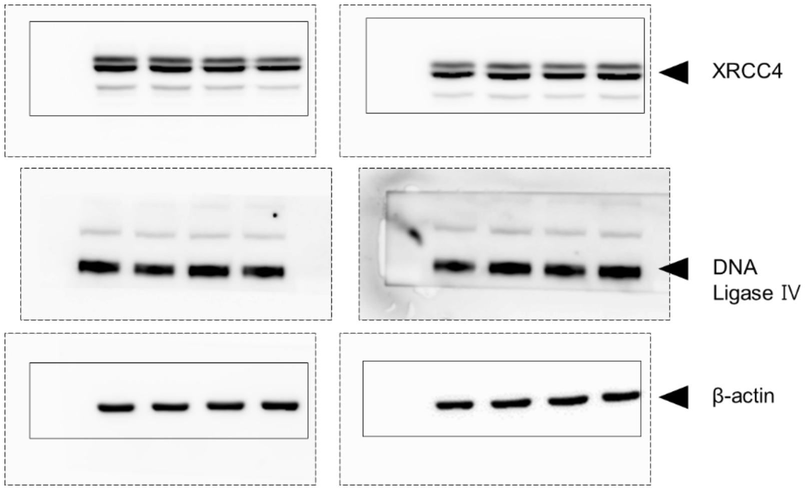

Supplementary Figure S14

Blots presented in Fig.

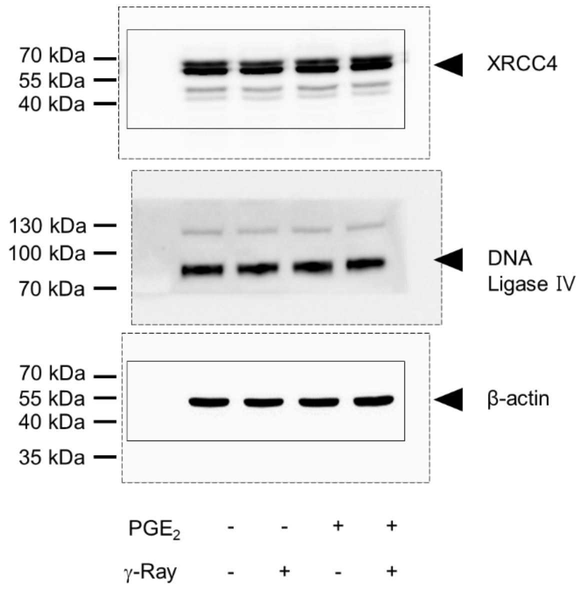

Supplementary Figure S14

Replicate blots

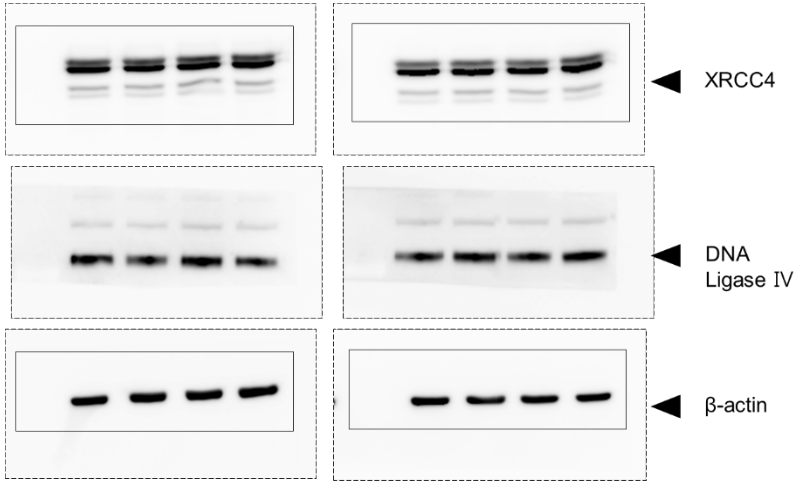

Supplementary Figure S21

Blots presented in Fig.

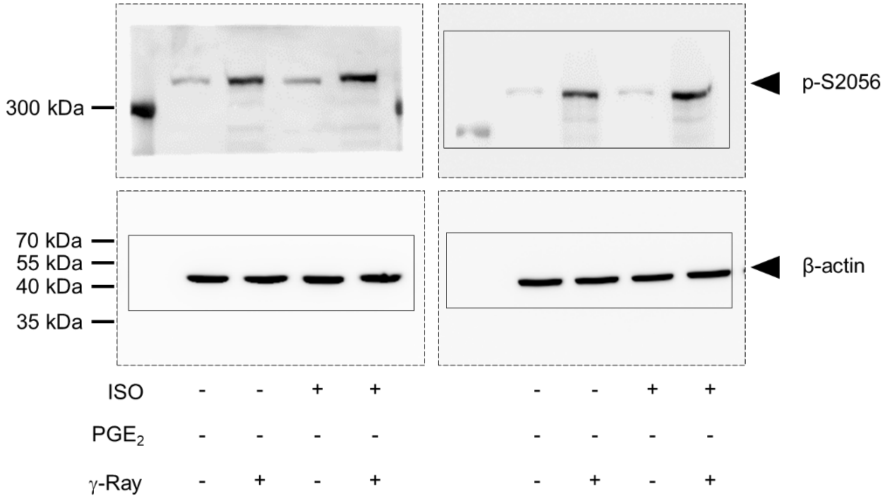

Supplementary Figure S21

Replicate blots

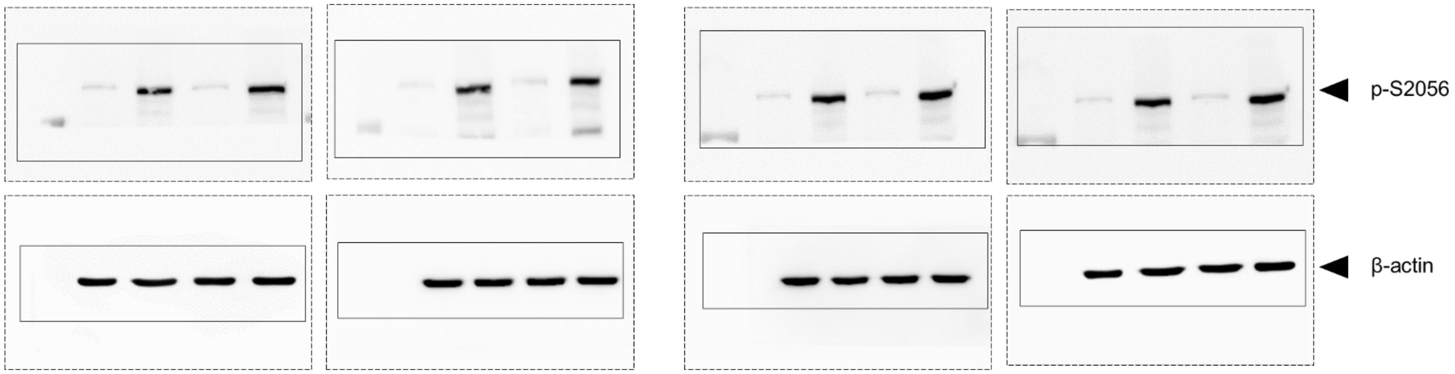

Supplementary Figure S24

Blots presented in Fig.

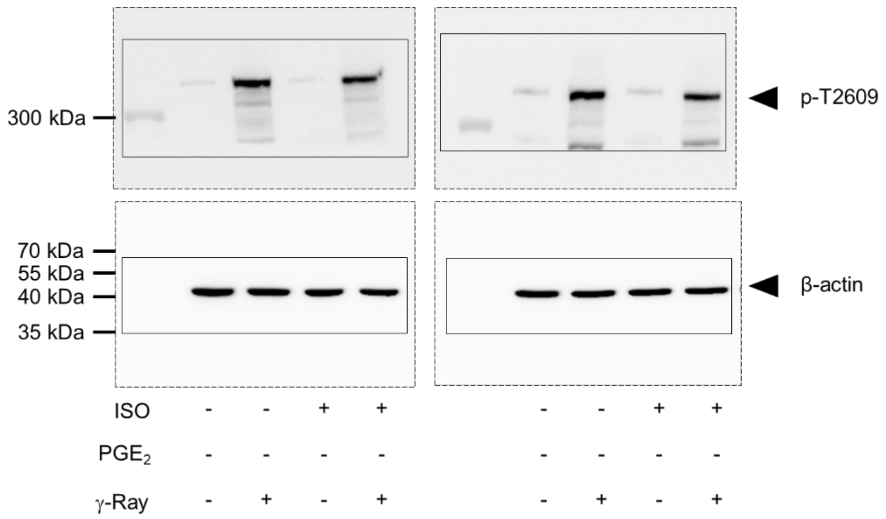

Supplementary Figure S24

Replicate blots

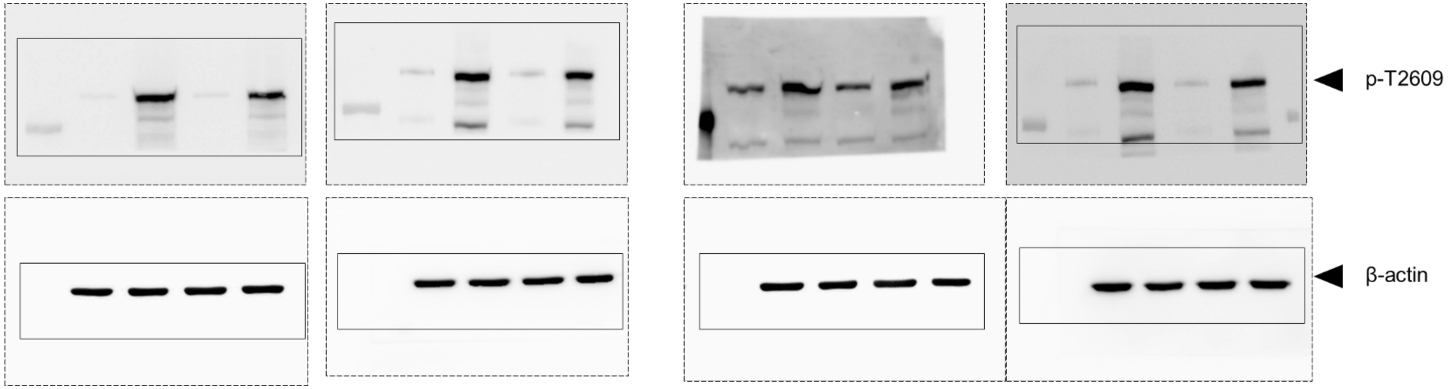

Supplementary Figure S25

Blots used for densitometric analysis

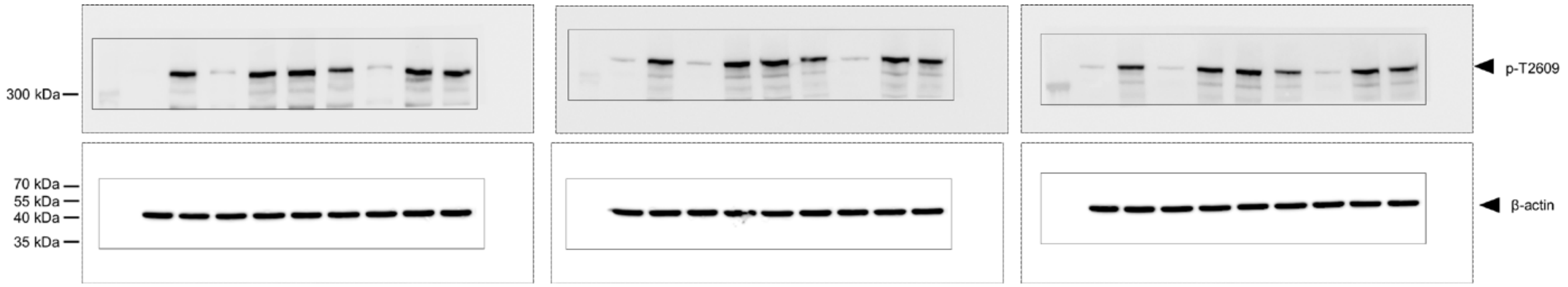

Supplement: Supplementary file 1 — Supplementary information. [file 41598_2020_71522_MOESM1_ESM.pdf]
